# A long-term mechanistic computational model of physiological factors driving the onset of type 2 diabetes in an individual

**DOI:** 10.1371/journal.pone.0192472

**Published:** 2018-02-14

**Authors:** Joydeep Sarkar, Gaurav Dwivedi, Qian Chen, Iris E. Sheu, Mark Paich, Colleen M. Chelini, Paul M. D'Alessandro, Samuel P. Burns

**Affiliations:** PricewaterhouseCoopers LLP, New York, New York, United States of America; UCLA, UNITED STATES

## Abstract

A computational model of the physiological mechanisms driving an individual's health towards onset of type 2 diabetes (T2D) is described, calibrated and validated using data from the Diabetes Prevention Program (DPP). The objective of this model is to quantify the factors that can be used for prevention of T2D. The model is energy and mass balanced and continuously simulates trajectories of variables including body weight components, fasting plasma glucose, insulin, and glycosylated hemoglobin among others on the time-scale of years. Modeled mechanisms include dynamic representations of intracellular insulin resistance, pancreatic beta-cell insulin production, oxidation of macronutrients, ketogenesis, effects of inflammation and reactive oxygen species, and conversion between stored and activated metabolic species, with body-weight connected to mass and energy balance. The model was calibrated to 331 placebo and 315 lifestyle-intervention DPP subjects, and one year forecasts of all individuals were generated. Predicted population mean errors were less than or of the same magnitude as clinical measurement error; mean forecast errors for weight and HbA1c were ~5%, supporting predictive capabilities of the model. Validation of lifestyle-intervention prediction is demonstrated by synthetically imposing diet and physical activity changes on DPP placebo subjects. Using subject level parameters, comparisons were made between exogenous and endogenous characteristics of subjects who progressed toward T2D (HbA1c > 6.5) over the course of the DPP study to those who did not. The comparison revealed significant differences in diets and pancreatic sensitivity to hyperglycemia but not in propensity to develop insulin resistance. A computational experiment was performed to explore relative contributions of exogenous versus endogenous factors between these groups. Translational uses to applications in public health and personalized healthcare are discussed.

## Introduction

Managing the care of people with diabetes is an enormous burden on the US healthcare economy, costing $176 billion per year in direct medical expenses according to estimates from 2012 [1]. The Centers for Disease Control and Prevention (CDC) estimate that in the US diabetes affects nearly 29 million individuals (~9% of US population) [2]. Diabetes significantly increases the risk of several co-morbidities including heart attack and stroke, retinopathy, risk of amputation, nephropathy, neuropathy, hearing loss, and depression among others [3–9]. In addition, an estimated 86 million people have prediabetes, which puts individuals at increased risk of type 2 diabetes (T2D), heart disease, and stroke [2]. While the prevalence of diabetes is stabilizing in the US, the global cost of diabetes is expected to increase by more than 50% by the year 2035 with developing countries being the largest contributors [10]. New approaches are required to curb the increasing global prevalence of T2D and improve care for patients with diabetes to control disease progression.

Many studies have evaluated the effects of interventions in high-risk patients [11–14]. A critical study was the Diabetes Prevention Program (DPP), which examined whether lifestyle adjustments and pharmacological interventions could prevent or delay the onset of T2D in prediabetic subjects over a four-year period [15]. The study found lifestyle interventions could reduce diabetes onset in prediabetic subjects by up to 50% over 4 years compared to a placebo group. Similar studies targeting prediabetic populations also found lifestyle interventions delay or even prevent the onset of diabetes [16–20]. Even with care taken to ensure subject adherence to interventions, such as in the DPP study [21], a significant portion of study subjects failed to avoid becoming diabetic. This emphasizes the need to improve upon simple, uniform interventions with ones specifically tailored to minimize the risk of diabetes onset in individual and patient cohorts. Despite recognition of the value of individualizing interventions to maximize long-term risk reduction, there are few methods to design interventions that take into account the subject’s current health state and clinical history. In the present work we have taken a significant step in bridging this gap by using mathematical modeling and computational simulations to study the dynamics of T2D onset and its multifactorial regulation by inherent individual characteristics and their interactions with lifestyle.

Several isolated computational models of diabetes-related biological systems and processes such as macronutrient metabolism, macronutrient energy balance, insulin response, and insulin resistance have been developed previously. Hall’s model of macronutrient energy balance effectively captures changes in body weight components by simulating various metabolic fluxes and was validated to predict body weight changes in response to caloric restriction [22]. Similar models have been developed independently that take energy balance into account to predict the dynamics of body weight [23,24]. A limitation of these models is that while they do phenomenologically model nutrient fluxes, they are not designed to fully account for mass balance, meaning that they do not contain equations that account for the conservation of mass of the macronutrients in the system. They also do not include the dynamics of glucose regulation by insulin or the pathophysiology of the development of insulin resistance or pancreatic decompensation as observed in prediabetic and diabetic subjects. Others have proposed integrative models to study population level dynamics of obesity and diabetes in the context of factors such as clinical management, environmental risks, and cultural norms etc., which are outside the realm of pathophysiology of diabetes [25,26]. Fallah-Fini et al. used Hall’s individual model to construct a population level representation of BMI distribution and the shifts in the distribution over time [27]. At the other end of the spectrum are models focused on dynamics at the cellular and molecular levels, such as those focusing on the dynamics of plasma glucose and its feedback effect on plasma insulin. For example, the so called minimal model of glucose dynamics was designed to capture the restoration of plasma glucose following an intravenous glucose tolerance test (IVGTT) [28]. A model developed by de Winter et al. simulates the feedback between glucose and insulin and also incorporates the dynamics of glycosylated hemoglobin (HbA1c) [29]. The model of De Gaetano et al. represents long term dynamics of glucose-insulin feedback and HbA1c along with regulation of beta cell mass and a phenomenological representation of insulin resistance [30]. More detailed models of insulin mediated cell signaling have been developed that represent the development of insulin resistance in various cell types as a function of inhibition of insulin signaling by multiple factors [31–33]. While these models are extremely useful for understanding diabetes related phenomena such as body weight change, glucose-insulin dynamics, and insulin resistance, they are limited by analyzing these aspects in isolation. Because the onset and progression of diabetes is a systemic process driven by complex interactions of all the phenomena listed above, it is necessary to study diabetes in an integrative way. Scaling up these models from representations of individual cellular or organ level processes to a whole-body model of diabetes is extremely valuable in understanding and predicting diabetes onset in response to various lifestyles and medications.

**Fig 1:**
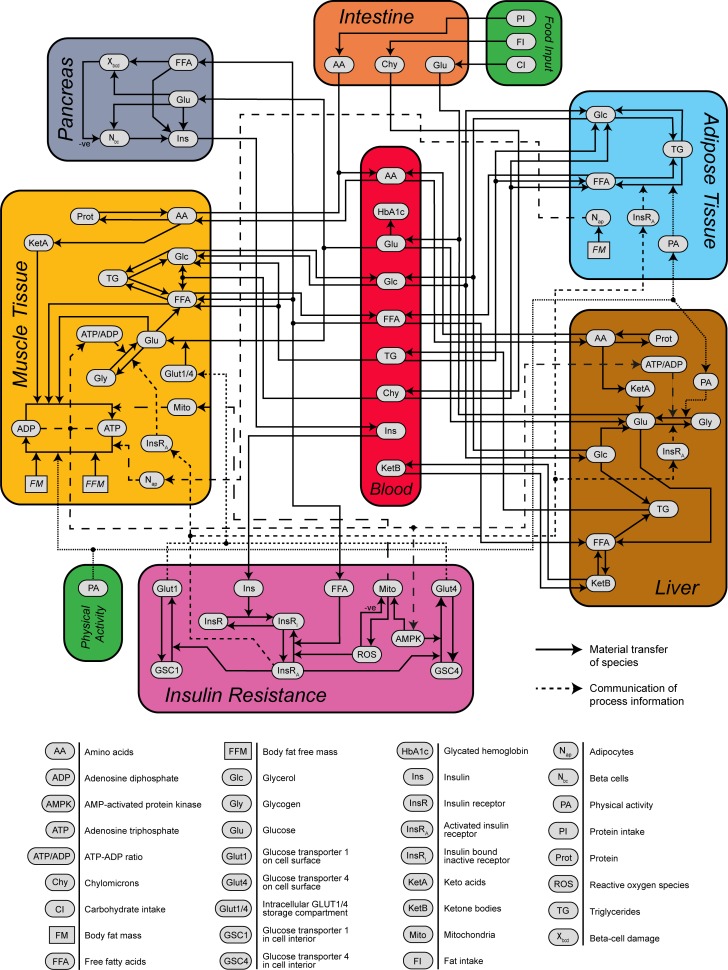
Schematic diagram of model components. Model compartments, internal component processes, flow of chemical species and metabolic information, and definitions of model variables.

The present work describes a holistic model of diabetes onset and progression that brings together the relevant physiological systems including mass balance of major macronutrients (carbohydrate, fat and protein), energy balance, regulation of insulin secretion, molecular mechanisms of development of insulin resistance, and the role of ROS and inflammation in diabetes progression. Our model is designed with a top-down structure comprised of multiple components that together represent the major biological processes leading to the development of pre-diabetes and onset of diabetes in humans. The model is divided into six components containing processes that span three scales: cellular (insulin resistance), distinct organs/tissues (adipose tissue, liver, muscle tissue, and pancreas), and whole-body (blood). Metabolic fluxes within each component, transport and other interactions between components are shown in Fig 1.

We chose to model the specific processes shown in Fig 1 because they form the structure required to correctly reproduce macronutrient metabolism as it pertains to long-term individual subject-level changes in body weight, development of insulin resistance, insulin decompensation, and irreversible progression to diabetes. It could be argued that a more parsimonious model is possible, but we have chosen this level of detail as it more readily enables investigation of the causal origins of observed model behavior, maintains a more specific connection to literature, and allows for future development to expand the capabilities of the model. Previous mathematical models of whole-body human metabolism have focused on energy balance in the body to predict the dynamics of body weight change [22–24]. In contrast to such models, the model presented here primarily focuses on mass balance of the three major macronutrients in the body, i.e., carbohydrates, protein and fat. The model also balances the energy of the entire system with the assumption that at a given time, energy consumption by essential processes (such as cell growth, repair etc.) is proportional to fat and fat free mass and together with physical activity, accounts for total energy needs of the body. Alteration in resting metabolic rate in response to weight change is included in model. This combination of mass and energy balance allows the model to simulate the dynamics of weight components (fat and fat free mass) along with various molecular markers of diabetes progression (Fig 1).

The resulting model is a multi-scale system of ordinary differential equations that spans cellular, tissue and whole-body scales and represents the dynamics of body weight, blood biomarkers of diabetes progression, the long term effects of lifestyle and is able to capture the development of pre-diabetes and onset of diabetes. We describe the design of the model, calibration of the model to individual subjects from the DPP placebo arm, apply the model to predict outcomes of the intervention arm, and perform retrospective, out of sample forecasts for individuals over a one-year period. The model is also applied to understand endogenous biological and exogenous lifestyle reasons for observed differences in HbA1c changes between individuals. A full description of the model equations and parameters, initial conditions, and sensitivity analysis are presented in supporting information.

## Model structure

### General mathematical model structure

Mathematically, the model is comprised of a system of ordinary differential equations. The general form of the differential equation is:

$$\frac{dC_{p}^{i}}{dt}=\frac{\sum_{j}\left(J_{p}^{j\to i}-J_{p}^{i\to j}\right)}{V^{i}}+\sum_{q}\left(R_{q,p}^{i}-R_{p,q}^{i}\right)$$

$$C_{p}^{i}(t=0)=C_{p}^{i}{|}_{t=0}$$

where $C_{p}^{i}$ is the concentration of species *p* in component *i*, *V*^*i*^ is the volume of component *i*, the mass fluxes between components *i* and *j* are represented as $J_{p}^{j\to i}$ and $J_{p}^{i\to j}$ and the conversions between different molecular species *p*, *q* in the component *i* are represented as $R_{q,p}^{i}$ and $R_{p,q}^{i}$. Detailed diagrams of the components show in Fig 1 are described with fluxes and their mathematical representations in S1, S2, S3, S4, S5 and S6 Figs and S1, S2, S3, S4, S5, S6 and S7 Tables). The conversion rates ($R_{p,q}^{i}$) are generally represented using one of three kinetic equations as appropriate:

1. Law of mass action with a single parameter and reaction rate proportional to substrate concentration with one parameter.
2. Michaelis-Menten equation with two parameters and reaction rate increasing hyperbolically before reaching saturation as the substrate increase.
3. Hill equation with three parameters, also saturable but with a sigmoidal increase in rate with substrate concentration.

The form of the equation to use for a particular reaction is either based on existing knowledge of how the reaction occurs or is chosen to fit the model to expected behavior. When no prior information was available about the reaction kinetics, law of mass action was assumed as the simplest default choice. The complexity was progressively increased to Michaelis-Menten and Hill equations if the simpler rate equations failed to reproduce the expected system behavior. When a reaction rate is known to be further modified by another molecular species (activation or inhibition), the functional form chosen as described above is modified appropriately to represent the corresponding increase or decrease in the rate.

### Blood

The blood component connects the other 5 components as shown in Fig 1 and provides the serum concentrations of many molecules (Fig 1, S1 Fig and S2 Table) that can then be compared to standard clinical measurements. The process of glycosylation of hemoglobin is incorporated in the blood component (flux v28 in S1 Fig and S2 Table), The representation of glycosylation enforces a threshold on the maximum percentage of hemoglobin that can be glycosylated, which produces an upper bound on the concentration of glycosylated hemoglobin (HbA1c). This term is a modification of the equation used by De Gaetano et al. [30]. The blood component interfaces with the terms related to the ingestion of food and delivery of macronutrients to the blood supply through the intestine. The processes of ingestion, digestion and absorption of food have been abstracted into direct delivery of the relevant forms of the macronutrients to the blood component. Specifically; carbohydrate consumption is represented as flux of glucose into blood, fat consumption is represented as flux of chylomicrons into blood, protein consumption is represented as flux of amino acids into blood. For long-term simulations, food intake is modeled as a continuous input of mass of each macronutrient consumed per unit time. This is a simplifying assumption we made to enable numerical integration of the model over long simulated time periods. For shorter bolus ingestion experiments, a simple first-order process represents the dynamics of the appearance of the macronutrients into blood upon ingestion.

### Muscle

The muscle component is an abstraction of all major tissues in the body other than pancreas, liver and adipose tissue, which have been distinguished in the model as their own separate components. The muscle component is where consumed macronutrients are used for ATP generation and ATP is expended for bodily functions, and physical activity. Metabolism of glucose and fat in the adipose tissue has also been lumped into the energy metabolism in the muscle component. The muscle component incorporates the transport of all 3 major macronutrients from blood e.g. uptake of free fatty acids (FFA) from chylomicrons and triglycerides etc. (Fig 1, S2 Fig). The component further represents the metabolic processes for the inter-conversion between different species, e.g. de novo lipogenesis (DNL), glycogenesis, glycogenolysis, lipolysis, proteolysis, and protein synthesis. The component incorporates the oxidation of macronutrients, phosphorylation of adenosine diphosphate (ADP) to adenosine triphosphate (ATP), and the hydrolysis of ATP to release energy (S1 Fig and S3 Table). The model assumes the concentration of molecular species in the interstitial space (ISF) and inside the cell is in rapid equilibrium. This assumption simplifies the model by allowing us to treat the muscle compartment as a single, well-mixed compartment. Hence concentrations in the muscle component can be taken to mean both intracellular and ISF concentrations unless specified otherwise. Details of specific processes within the muscle component are described below.

## Insulin-dependent and independent glucose transport

The transport of glucose into the muscle component is driven by glucose gradient, and the permeability of glucose is defined as a non-linear function of the concentrations of GLUT1 and GLUT4 transporters on the cell surface (v1 in S1 Fig and S3 Table). The GLUT1/GLUT4 cell surface concentrations are inputs from the insulin resistance (ISR) component. As described in the ISR component, GLUT1 acts as the constitutive transporter of glucose. Its trafficking is relatively weakly regulated by insulin and it acts as the insulin-independent glucose transporter. The cellular trafficking of GLUT4 is strongly insulin dependent and it acts as the insulin-dependent glucose transporter of glucose. Glucose transport into cells is assumed to increase linearly with GLUT1 and in a sigmoidal manner with GLUT4, based on previously reported sigmoidal response of GLUT4 to increasing insulin concentration [34]. As described in the section “General mathematical model structure”, the simplest possible model was chosen for GLUT1, and a more complex model was used for GLUT4 in view of the supporting experimental data.

Continuous cycling of GLUT1 and GLUT4 ensures there is always some concentration of glucose transporter present on cell membrane, irrespective of insulin levels (Yang 1993). By including this mechanism, the model accounts for insulin-independent glucose disposal. Furthermore, since glucose transport is dependent on glucose concentration (e.g., v_1_^MUS^ in S3 Table), increase in glucose concentration above baseline level upregulates glucose uptake by cells independently of available insulin, thus satisfying part of cellular glucose requirement and slowing down endogenous glucose production. The notion of glucose effectiveness, which is defined as the ability of glucose to enhance its own uptake and inhibit its production [35,36], is accounted for by this mechanism.

## De novo lipogenesis

While the liver is the main site for de novo lipogenesis (DNL), in conditions of excess glucose inside the muscle component, there is conversion to fat in this component as well. The conversion of glucose to fat is modeled as a Hill function such that significant lipogenesis only happens in conditions of significant excess of glucose over steady-state conditions (v8 in S2 Fig and S3 Table).

## Glycogenesis and glycogenolysis

The model represents the dynamic equilibrium between glucose and glycogen in the muscle component. In the model, glycogen is made from 4 glucose molecules and this stoichiometry is maintained throughout the model. Any excess glucose is converted to glycogen until a maximum glycogen threshold is reached. As the simplest possible model, the rate of glycogenesis was assumed proportional to the concentration of glucose in the tissue, insulin sensitivity (IS, an input from the ISR component) and the remaining availability for glycogen storage. The glycogenolysis process is proportional to glycogen concentration but is further controlled by the energy state of the muscle cells measured as the ATP/ADP ratio (AAR; terms v7 and v10 in S2 Fig and S3 Table).

## Fatty acid uptake

Serum triglycerides (TGs) and chylomicrons serve as the primary sources of fat to the muscle component. Lipoprotein lipase (not explicitly modeled) hydrolyses circulating TGs and chylomicrons (v13 in S2 Fig and S3 Table) into free fatty acids (FFAs) and glycerol in the muscle component. Glycerol transport back into blood is represented as a first order mass action (v4). There is bi-directional transport of FFAs between blood and muscle component (v2, v3).

FFA, glycerol and TG exist in a dynamic equilibrium (v16, v17) where three FFA molecules combine with one glycerol molecule to create one TG molecule, and this stoichiometry is preserved throughout the model. In reality it is glycerol-3-phosphate (gly-3-P) that is used in esterification of FFAs. The generation of gly-3-P is known to occur by several pathways including direct phosphorylation of glycerol by glycerol kinase, from glucose via glycolysis, or from pyruvate through glyceroneogenesis [37]. Glycerol released by hydrolysis of TG can be utilized for gluconeogenesis, and then released as gly-3-P during glycolysis, finally cycling back to form TG. Instead of explicitly modeling the steps of this cycle and glyceroneogenesis through intermediates of other metabolic pathways, we assumed that there is a fixed pool of glycerol available systemically that cycles between bound (as TG) and free forms.

## Amino acid uptake and protein metabolism

The amino acid fluxes in and out of the muscle component are represented as first order mass action processes (v5, v6). Inside the muscle component amino acid and protein concentrations are in dynamic equilibrium with a stoichiometric ratio of 500 (v16, v17). Amino acids are converted into ketoacids (v18) for use in energy metabolism (S2 Fig and S3 Table). The contribution of amino acids to energy production is smaller than that of carbohydrates and fats. The main purpose served by amino acids is to maintain the body’s nitrogen balance. Since we have not simulated starvation, the amino acid metabolism component is designed to maintain a steady nitrogen balance and excess protein is excreted [38].

## Macronutrient oxidation and ATP hydrolysis

The muscle component is the main site of macronutrient oxidation and ATP hydrolysis. Glucose, fat, and ketoacids fuel the ATP synthesis process in the model. The model only represents aerobic oxidation of the fuels as a single step reaction. The rate of ATP generation from all three types of fuels is proportional to the concentration of ADP and mitochondrial function (input from ISR component). The rate of oxidation of ketoacids is directly proportional to the concentration of ketoacids in the components while the rates of oxidation of glucose and FFA are represented as saturating processes (terms v19, v9 and v13 in S2 Fig and S3 Table). Since systemic protein levels are maintained at an approximately steady level in the model (as described in “Amino acid update and metabolism”), a simple mass action term was sufficient to capture their nearly constant contribution to energy production under non-starvation conditions. Glucose and FFA are the main contributors to ATP generation regulated by a series of enzymes. The complex process of oxidative phosphorylation was simplified to a single mathematical expression representing the saturable nature of enzyme catalyzed processes. The three fuels lead to the generation of different amounts of ATP and this stoichiometry is also incorporated into the equations. The stoichiometry is tuned such that the respiratory quotient is 0.85 at steady state and ketoacids contribute approximately 20% to ATP synthesis.

The hydrolysis of ATP to ADP is proportional to available concentration of ATP in the muscle component; however, several additional mechanisms regulate the rate of hydrolysis. The rate of ATP hydrolysis is assumed to increase with differential change in fat mass (FM) and fat-free mass (FFM) as compared to baseline. Use of differential change instead of the absolute mass is a recognition of the fact that the same amount of weight change in two individuals with different baseline weights may lead to different levels of perturbation in energy balance. The model differentiates between energy expenditure for regular body function defined as basal metabolic rate (BMR) and physical activity, so changes in energy expenditure due to changes in physical activity are also incorporated into the ATP hydrolysis equation. It has been shown previously that decrease in metabolic rate following weight loss cannot be fully accounted for by changes in FM and FFM [39–41]. To account for this adaptation in metabolic rate, the model incorporates regulation of metabolic rate through mechanisms independent of FM and FFM. There is evidence the central nervous system, through the action of leptin and other factors, is an important regulator of basal metabolic rate [42]. We abstracted the effects of leptin and other molecular level causes of BMR adaptation into a single simulated mechanism whereby leptin, whose secretion is regulated by the average concentration or loading of adipocytes with triglycerides (input from adipose component), modifies the basal metabolic rate (BMR) through a Hill function such that the BMR decreases as leptin levels go up. It has also been suggested the gut microbiome plays a role in glucose homeostasis [43], but there does not exist an established consistent literature that can be used to guide a mechanistic representation of this process.

### Liver

Liver is the third major component involved in macronutrient metabolism. Many of the processes incorporated in the muscle component are also present in the liver component (Fig 1, S3 Fig and S4 Table). The aspects that are different between liver and muscle are described in the following sections.

## GLUT1/GLUT4 mediated glucose transport

The mechanism of glucose uptake by the liver component (v1, v2 in S3 Fig and S4 Table) follows a similar mechanism as that in the muscle component. However, unlike the muscle where there is no net outflow of glucose from the muscle component into blood, there is secretion of glucose from the liver into blood. Hence, the influx and efflux terms are separated. The influx of glucose is represented as a function of GLUT1/GLUT4 and serum glucose. The efflux term (v2 in S3 Fig and v_2_^LVR^ in S4 Table) is separately represented and depends on liver glucose concentration.

## De novo lipogenesis

The liver is the primary site for de novo lipogensis (DNL) in the body. The expression for DNL (v12, S3 Fig and S4 Table) in liver is the same as that in the muscle component.

## Glycogenesis and glycogenolysis

Glycogen-glucose dynamics in the liver component are represented the same way as in the muscle component. During rigorous physical activity liver glycogen is preferentially used as a source of glucose [44]. It has been reported that the suspected mechanism for this enhanced glycogenolysis is increase in levels of catecholamines like epinephrine [45,46]. Hence, the form of the function for change in glycogenolysis follows data for levels of epinephrine with increasing intensity of exercise [47].

## Gluconeogenesis from amino acids and glycerol

The liver component is the site of gluconeogenesis. The model incorporates gluconeogenesis from glycerol as well as from a glucogenic portion of amino acids. Gluconeogenesis from ketoacids is assumed to be proportional to the concentration of ketoacids in the liver (v17, v21 in S3 Fig and S4 Table). Gluconeogenesis from glycerol is inhibited by increased insulin sensitivity (input from the ISR component).

Lactate produced by anaerobic glycolysis is an important source of gluconeogenesis through the Cori cycle. We have ignored anaerobic metabolism in our model and did not include lactate in our model since it was not our goal to simulate intense muscular activity resulting in cellular oxygen deprivation.

## Fatty acid metabolism

Unlike in the muscle, in the liver, there is no uptake of FFA from TGs and chylomicrons. There is uptake of FFAs from blood into liver that follows a 1^st^ order process (v3 in S3 Fig and S4 Table). New FFAs that are synthesized through DNL and FFA absorbed from blood are combined with glycerol to form TG that is transported out (v14, v4). Lipolysis of TG in the liver is ignored.

## Ketogenesis

The model also incorporates a simplified representation of the ketogenesis (v15 in S3 Fig and S4 Table) that increases significantly when there is excess FFA in the liver component. Some ketone bodies are reconverted to FFA (v16) while the rest are transported to blood over a gradient (v7, v8).

## Adipose

The adipose component represents the site of both visceral and subcutaneous fat tissue. The adipose component incorporates uptake of fat from blood, and the dynamic equilibrium between lipolysis and esterification of FFA. The fatty acid metabolism in adipose tissue is modeled similarly to the muscle component (Fig 1, S4 Fig), with difference being that adipose tissue takes up more FFA from circulating TG and chylomicrons than muscle. The increase in lipolysis by physical activity [48,49] is captured in the model. It has also been shown that the increase of lipolysis saturates with increased intensity of physical activity [50]; therefore, a saturating function is used to connect lipolysis with increasing intensity of physical activity. Finally, regulation of lipolysis by insulin is also included in the model such that increased insulin sensitivity (input from ISR component) decreases the rate of lipolysis [51] (v4 in S4 Fig and S5 Table).

## Pancreas

The proposed pancreas model is a modification of that presented by De Gaetano et al. [30]. The model incorporates the long-term dynamics of beta cell mass, beta cell function and insulin production as distinct elements (Fig 1 and S5 Fig). While the fasting plasma insulin is predominantly dictated by the dynamics of the beta cell number, the increased response of insulin secretion to increased serum glucose is predominantly driven by beta cell function. Proliferation of beta cells is represented as a Hill function dependent on blood glucose concentration (v1 in S6 Table). The choice of the Hill function implies that beta cell growth, and hence subsequent insulin production, is low for very low glucose concentrations and increases rapidly toward a saturation point when glucose concentration crosses a certain threshold. Beta cell apoptosis is a function of chronic inflammation (v2). Beta cell function, a value bounded between 0 and 1, decreases at a rate proportional to remaining beta cell function and ROS concentrations. The beta cell function recovers by a first order mass action process. Beta cell function was introduced to take into account the lack of insulin producing capacity in the remaining beta cells in diabetic subjects. The rate of insulin production is proportional to the beta cell mass, beta cell functional capacity, and also increases in a saturating manner (through independent Hill functions) with serum glucose and FFA concentrations. Insulin is removed from blood by a simple first order mass action process.

Hormones counter-regulatory to insulin, in particular glucagon which is secreted by the alpha cells of the pancreas, were not explicitly modeled. Since the secretion of insulin and glucagon is reciprocally regulated in the maintenance of glucose homeostasis with paracrine and endocrine effects of insulin determining glucagon levels [52], we reasoned that the dynamics of insulin contain sufficient information to estimate the effect of counter-regulatory hormones and incretins. We used this as the basis to simplify the model and only include insulin as an explicit hormonal regulator of glucose homeostasis. As effects of glucagon and other counter-regulatory hormones of insulin are to counteract the action of insulin, the parameters related to insulin action were adjusted to achieve the net effect of the opposing mechanisms.

## Insulin resistance

The insulin resistance (ISR) component (Fig 1, S6 Fig and S7 Table), represents processes that modulate the following: a) the response of cells to insulin, simulating deficiency in insulin action as insulin resistance increases; b) short and long-term regulation of mitochondrial function; and c) the effects of energy depletion on AMPK activity. Binding of insulin to the transmembrane insulin receptors leads to phosphorylation and activation of downstream signaling resulting in translocation of glucose transporters GLUT1 and GLUT4 to the membrane [53,54]. Reactive oxygen species (ROS), FFA and pro-inflammatory cytokines (e.g. IL-6) have been shown to inhibit insulin signaling and contribute to insulin resistance [55–60]. Since the dynamics of insulin receptor phosphorylation and dephosphorylation are significantly faster than the dynamics of metabolic fluxes simulated in the model (such as glycogenesis, lipolysis, insulin secretion etc.), a quasi-steady state approximation was used to convert the differential equations representing the dynamics of insulin receptor activation into algebraic equations. Insulin sensitivity is expressed as the fraction of insulin receptors that are in the phosphorylated (active) state (S6 Fig and S7 Table).

The concentrations of GLUT1 and GLUT4 on the cell surface regulate the glucose transport into cells. The model represents the dynamics of GLUT1 and GLUT4 concentrations on the cell surface as well as the intracellular concentrations. Trafficking of GLUT1 and GLUT4 to the cell surface is controlled by phosphorylated insulin receptors and the membrane bound forms are constitutively endocytosed. While the trafficking of GLUT4 is primarily insulin dependent, that of GLUT1 is weakly regulated by insulin. In particular, insulin driven exocytosis of GLUT4 is much stronger than that of GLUT1 (exocytosis rate constant of GLUT4, k_gsc4,glut4_ins_, is four times that of GLUT1, k_gsc1,glut1_ins_, as shown in S8 Table). Consequently, GLUT1 acts as the constitutive transporter regulating insulin-independent glucose uptake and is assumed to represent other types of constitutively active glucose transporters in various tissue types. GLUT4, on the other hand, is strongly regulated by insulin and controls insulin-dependent transport of glucose into cells. GLUT4 transport to the cell surface is additionally known to be upregulated by AMPK activity and is represented in the model [61,62]. GLUT1 and GLUT4 in intracellular storage components (GSC1 and GSC4) are synthesized at a constant rate and degraded by a first order mass action process.

Mitochondrial function (half-life of 3 days [63]) controls macronutrient oxidation, thereby controlling the rate of generation of ATP. In this model, it is assumed mitochondrial concentration represents mitochondrial function. It is further assumed leakage of mitochondrial ROS from the electron transport chain is the major source of systemic ROS. Cumulative effects of ROS over the process of aging are represented by slow accumulation of instantaneous ROS (S7 Table) leading to degradation of mitochondrial function. This structure allows the model to reproduce the reduced mitochondrial function as observed in aging studies [64]. Due to this age-dependent reduction in mitochondrial concentration, macronutrient oxidation in the model decreases over time, causing changes in weight and body-fat content with age for a constant diet and activity level.

ATP deficiency (or AMP excess) leads to upregulation of AMPK activity, which in turn enhances mitochondrial function [62,65]. In the model, concentration of adenosine monophosphate (AMP) is assumed to be proportional to that of ADP. ADP (hence AMP) increases AMPK activity through a Hill function in the model and mitochondrial synthesis is assumed to be directly proportional to AMPK activity (S7 Table).

### Body weight

Body weight is a direct function of the total body glycogen, protein, fat mass, fat-free mass, and hydration, making body weight tightly connected to mass and energy balance in the model. The calculations of body-weight and its components (FM and FFM) are adapted from the model presented by Hall [22]. The following equations were used for computing body weight components:

$$M_{gly}=(C_{gly}^{LVR}\times V^{LVR}+C_{gly}^{MUS}\times V^{MUS})\times {MG}_{gly}$$

$$M_{pro}=(C_{pro}^{LVR}\times V^{LVR}+C_{pro}^{MUS}\times V^{MUS})\times {MG}_{pro}$$

$$M_{FM}=(C_{tg}^{LVR}\times V^{LVR}+C_{tg}^{MUS}\times V^{MUS}+C_{tg}^{ADI}\times V^{ADI})\times {MG}_{tg}$$

$$M_{FFM}=\frac{\chi_{FM}\times M_{FM}+M_{gly}+M_{pro}+M_{bone}+M_{ecp}}{1-\chi_{FFM}}$$

$$BW=M_{FM}+M_{FFM}$$

In the above equations, *M*_*i*_ represents the mass of the species *i*, $C_{i}^{j}$ is concentration of *i* in component *j*, *V*^*j*^ is the volume of component *j*, *χ*_*i*_ represents the hydration coefficient of *i*, *MG*_*i*_ is molecular mass of species *i*, and *BW* is the total body-weight. These equations show body-weight is a direct function of the changes in amounts of the relevant molecular species; thus, body-weight is tightly connected to mass and energy balance in the model.

### Physical activity

The model incorporates the effects of physical activities at multiples places. A fraction of the total energy expenditure represented by ATP hydrolysis is attributed to physical activity which include any specific exercise routines. Changes in lifestyle of an individual, which result in changes in physical activity, lead to proportional changes in the fraction of ATP hydrolysis attributed to physical activity. Physical activity, especially high intensity exercises, have been shown to lead to rapid release of glucose from the liver through the process of glycogenolysis [47]. This effect is known to be mediated through increased release of epinephrine. Though the model does not currently incorporate epinephrine, the increased glycogenolysis leading to reduced liver glycogen is represented in the model. Physical activity in the model has been represented in the model to lead to increased lipolysis. It has been reported that the increase in lipolysis is a function of fraction of VO2 max of an individual [66,67] and that functional representation is incorporated in the model.

## Materials and methods

### Data processing

The publicly released Diabetes Prevention Program (DPP) data set from the National Institute of Diabetes and Digestive and Kidney Diseases (NIDDK) [15] was used to calibrate and validate the model. We examined individual subject data collected from participants in the placebo and lifestyle-intervention trial arms of the DPP study. Subjects with at least three years of data were selected to have sufficient data to study the time-course of their health. The variables extracted for the analysis are listed under “Variable Name” column of Table 1. Study subjects were grouped according to their intervention arm assignment. Individuals who did not meet the required number of data points per variable (Table 1), had height < 158 cm, BMI < 27, opposing trends in HbA1c versus glucose/insulin measurements, and/or were pregnant were excluded from the analysis. The remaining subjects’ data were cleaned to remove duplicates, missing rows, extreme outliers (1 subject with weight gain > 75%; 2 subjects with outlying HbA1c). A total of 331 subjects from the placebo arm and 315 from the lifestyle intervention arm were used for our analysis.

**Table 1:** DPP data requirements.

Variable Name	Data Requirements
Subject ID	1 value
Gender (male, female)	1 value
Age (years)	1 value
Initial BMI (kg/m^2^)	1 value
Height (m)	1 value
Study arm (placebo, lifestyle-intervention)	1 value
Weight (kg)	≥ 7 measurements
HbA1c (%)	≥ 5 measurements
Fasting Insulin (μU/mL)	≥ 4 measurements
Fasting Plasma Glucose (mg/dL)	≥ 7 measurements
Daily Diet (kCal)	≥ 2 measurements

#### Numerical solution of model equations

The model consists of a series of first order ordinary differential equations (ODEs) and algebraic equations coded in Python. The ODE system was numerically integrated forward in time using the LSODA routine in the scipy.integrate submodule of the SciPy Python library [68,69]. LSODA dynamically accounts for stiffness in the equations over the course of the simulation. The numerical simulation of an individual is started at age 20 in an assumed healthy state with a fixed set of initial conditions and integrated forward to current time. The model is fit so that when the numerical integration reaches the current time, the outputs of model match the individual's specific data. A detailed list of the initial conditions at age 20 is provided in S11 Table. Interventions or changes in lifestyle are implemented by stopping the integration at the time of the change of lifestyle, changing model parameters and/or inputs (e.g., reduced diet, increase physical activity) to introduce the intervention into the simulation, then restarting the model at that time and running forward to the end of simulation. The model is run for an individual, and populations are represented by pooling the collection of individual model simulations to generate population information.

#### Model calibration technique

The parameters of the model were estimated using the differential evolution optimization algorithm [70] to minimize a weighted least squares objective function. The specific form of the objective function, Ф, given the set of model parameters, *θ*, was:

$$\Phi (\theta )=\sum_{i}\sum_{j}w_{ij}{\;\{y_{ij}(\theta )-y_{ij}^{*}\}}^{2}$$

where y^*^_ij_ represents measured DPP data, *y*_*ij*_ (*θ*) represents model outputs for parameter set *θ*, and *w*_*ij*_ are weights specific to the data point. The index *i* indicates the variable being fit, and the index *j* indicates the times at which *y*_***i***_ was measured. To determine whether the match of the model output to the data was adequate, measurement errors and natural variability in the measured variables were used to assess the fit. Population size was set at 7 times the number of parameters to be optimized and convergence was generally observed in less than 200 iterations. The parameter search was considered to have converged numerically when the best score did not change by more than a pre-set threshold, or the coefficient of variation for any of the parameters in the population was lower than 5%. The weights *w*_*ij*_ were determined empirically on some test cases and were adjusted to obtain the best qualitative fit as determined by visual comparison of the model output and training data. The weights determine the contribution of each variable or data point to the goodness of fit score (objective function). Variables known to be more reliably measurable (e.g., body weight), were assigned higher weights than those less so (e.g., fasting plasma glucose). Additionally, in situations where the optimization algorithm fit a small number of data points very well while generating poor fits for a large number of other data points, the weights of the points were adjusted to ensure a better overall fit.

### Baseline model calibration

Before simulating individual subjects, the model was calibrated to data from multiple studies to generate a canonical set of 109 parameters representing the “typical” individual. A variety of data ranging from intracellular cell signaling data to clinical study data were used for this purpose. The calibration was accomplished in broadly four stages: 1) obtain parameters directly available in the literature and set corresponding model parameters to those values; 2) calibrate parameters of intracellular pathways (Table 2); 3) calibrate parameters related to metabolic response to interventions in healthy individuals (Table 3); 4) calibrate to interventions in overweight/obese individuals in the context of the development of pre-diabetes, onset of diabetes and response to lifestyle interventions (Table 4). The complete list of parameters that were fit along with the specific studies used are provided in S8, S9, S10 and S12 Tables. The parameters obtained after these four stages of model calibration were used to define the “baseline model”, which represents the typical individual and produces reasonable responses to dietary and physical activity interventions. The baseline model served as the starting point for individual model calibration to individual subjects in the DPP study.

**Table 2:** Intracellular calibration processes and studies.

Intracellular studies	References
Insulin receptor phosphorylation, dephosphorylation, GLUT1/4 translocation and response to insulin	[31,58,71–73]
Change in de novo lipogensis in response to increased availability of glucose	[74,75]
Intracellular glucose metabolism	[66,76]
AMPK activation by AMP; AMPK mediated processes	[77–79]
Insulin resistance due to FFA, ROS, inflammation; ROS accumulation	[34,57,58,80–83]

**Table 3:** Healthy metabolic calibration processes and studies.

Healthy metabolic studies	References
Carbohydrate, fat, protein, energy homeostasis and overfeeding	[38,84–98]
Short-term and longer duration starvation studies	[99,100]
Physical activity of differential intensities and durations	[66,101–106]
Intravenous lipid infusion studies	[58,85–87]
Glucose bolus ingestion studies	[107]
Low/high fat, low/high carbohydrate diet studies	[74,75,108,109]
Insulinic/glycemic clamp studies	[110]

**Table 4:** Diabetes related calibration processes and studies.

Diabetes related studies	References
DPP study averages for both placebo and lifestyle intervention arms	[15]
Study with large change in lifestyles	[15,111]
Study with dynamics of pancreatic decompensation	[112,113]
Study accounting for changes in metabolic rate with changes in FM and FFM	[100,111,114,115]

#### Individual model calibration

To fit the model to individual subjects in the DPP study, parameter values of the baseline model calibration were used as a starting point (see Methods: Baseline model calibration and Model calibration technique). The initial condition of the model corresponds to a lean individual of age 20. Most initial conditions were obtained from known normal levels of serum biomarkers, normal levels of storage of glycogen, and fat in different tissues and the rest were computed by assuming an equilibrium condition at the initial state. A subset of parameters was further refined to customize the baseline model to individual data from the first 3 years of the DPP study. The following data were used for individual calibration: age, height and gender of each subject were provided as inputs to the model; time courses of weight, *BW* (kg), HbA1c, $C_{hba1c}^{BLD}$ (%), fasting serum glucose, $C_{glu}^{BLD}$ (mM), and fasting serum insulin, $C_{ins}^{BLD}$ (mM) were used to calibrate the model to individual subjects. Initial analysis indicated using self-reported diets to calibrate the model resulted in poor fits. Previous studies have shown that self-reported caloric intake data can be inaccurate [116,117]. To account for the possibility of diet misreporting, we introduced two additional parameters that account for misreporting of fat and carbohydrates.

The large number of model parameters available to be fit to an individual could result in parameter degeneracy, where arbitrary sets of parameters that could be tuned to fit the time course data. To deal with this, we sought to find the smallest set of physiologically relevant parameters that would allow us to obtain reasonable model fits. We started by calibrating six parameters to individuals (four related to diet requirements, and one each related to glucose and HbA1c) and leaving the rest of the parameters fixed at the values in the baseline model, but found we could not satisfactorily explain all the observed data points. We sequentially expanded the list of calibrated parameters to a set of twelve parameters, related to the measured data, which could be varied on an individual subject basis and enabled the model to be fit. The names of the parameters and their biological meanings are provided in Table 5. The basal requirement of macronutrients to maintain constant body weight was assumed to be different for each individual. This assumption is justified by our knowledge that different individuals have different dietary requirements due to individual variability. Since no information was available from the study about the basal macronutrient requirements of study subjects, the basal carbohydrate and fat requirements per day (*CI*_0_ and *FI*_0_) were calibrated for each individual. Additionally, ratios of actual intake to baseline intake (*CI*/*CI*_0_, *FI*/*FI*_0_) in the period between 20 years of age to the beginning of study and the ratio during the study (*CI*_2_/*CI*_0_, *FI*_2_/*FI*_0_), which accounts for potential misreporting, were also calibrated individually. It was observed that subjects at similar levels of weight had different levels of serum glucose and the rates of increase in serum glucose were different between individuals. To reproduce the variability, two parameters related to insulin resistance, *α*_*dep*_*ffa*_ and *k*_*dep*_*ffa*_, were included in the estimation process. We observed in the data that individuals with similar fasting glucose levels can have different HbA1c levels. To capture this inter-individual variability two parameters related to the rate of synthesis of HbA1c, $Cmax_{hba1c}^{BLD}$ and $C_{hba1c}^{BLD}(t=0)$, were included. It is likely that the observed variability in HbA1c is due to differences in postprandial glucose excursion across individuals, and not due to differences in maximal concentration of HbA1c. However, meal-to-meal data and corresponding glycemic excursions were not available to us and we had to choose $Cmax_{hba1c}^{BLD}$ as the best available proxy. Baseline values of fasting plasma glucose and insulin were independent of each other across subjects. During the study, while the serum glucose of most subjects increased, the serum insulin of some subjects increased while decreasing in others. To capture this individual variability, two parameters related to the dynamics of pancreatic insulin production, *KM*_*s*,*ins_glu*_ and *α*_*bc*,*s_ros*_, were optimized for each subject. All other model parameters fit from baseline calibration were assumed to remain constant across all individuals analyzed and are listed in the S8, S9, S10 and S11 Tables. Adjusting only these 12 parameters was sufficient to generate acceptable fits for individuals in the analysis.

**Table 5:** Model parameters fit to individual subjects.

Parameters fit to individual DPP subjects	Parameter abbreviation	Placebo Population mean (SD)	Biological Range	Reference
Basal carbohydrate requirement to maintain steady body weight	*CI*_0_	130.6 (48.0) gm	50 – 800g	[118]
Basal fat requirement to maintain steady body weight	*FI*_0_	49.1 (19.4) gm	10 – 300g	
Relative increase over basal carbohydrate intake prior to study	*CI/CI*_0_	1.50 (0.27)	1.0–3.0	Estimated
Relative increase over basal fat intake prior to study	*FI/FI*_0_	1.32 (0.35)	1.0–3.0	
Relative increase in carbohydrate intake during the study	*CI*_2_*/CI*_0_	1.41 (0.33)	0.5–2.0	
Relative increase in fat intake during the study	*FI*_2_*/FI*_0_	1.31 (0.42)	0.5–2.0	
Maximal serum HbA1c concentration	$Cmax_{hba1c}^{BLD}$	41.3 (32.7)	10–100	[119]
Initial HbA1c concentration	$C_{hba1c}^{BLD}(t=0)$	4.57 (0.41)	4–8	
Maximal inhibitory effect of FFA on insulin signaling	*α*_*dep_ffa*_	15.1 (8.40)	1.0–30.0	Estimated
FFA concentration for half maximal inhibition of insulin signaling	*k*_*dep_ffa*_	18.5 (15.6)	2.0–48.0	
Strength of pancreatic beta cell damage due to glucotoxicity, lipotoxicity and inflammation	*α*_*bc*,*s_ros*_	8.73 (5.10)	1.0–15.0	
Fasting plasma glucose required for half maximal insulin production rate	*KM*_*s*,*ins_glu*_	13.1 (5.05)	2.0–20.0	

Parameters were fitted using differential evolution algorithm (see Methods: Model calibration technique) to minimize the objective function

$$\Phi (\theta )=\sum_{i}\sum_{j}w_{ij}{\;\{y_{ij}(\theta )-y_{ij}^{*}\}}^{2}$$

where *y*_*i*,*j*_ are the study data, $y_{i,j}^{*}$ is the corresponding model simulation of variable *j*, *i* indicates the time points at which data were recorded over the first 3 years of the study, *N* is the number of data points corresponding to variable *j*, and ${\bar{y}}_{j}$ is the mean of the study data recorded for variable *j*. The normalized root mean squared error,

$$NRMSE=\sum_{j}\frac{1}{\bar{y_{j}}}\sqrt{\frac{\sum_{i=1}^{N}{\left(y_{ij}-y_{ij}^{*}\right)}^{2}}{N}}$$

was used to determine the error in the resulting fit.

#### Initial conditions

The exact initial conditions of all species for any subject at a particular point in time are not possible to determine, as such, we assumed all simulated individuals were lean at age 20, eating a diet equal to their metabolic need, and at quasi-steady state at that time point. A similar assumption was used by De Gaetano et al. [30] to initialize their pancreatic model. Using this assumption, several parameters and initial conditions of states are solved for based on known parameters and initial conditions. A list of the initial conditions used are listed in S11 Table.

#### Nearest neighbor estimation

To determine the nearest neighbor, attributes (age, gender, height, baseline weight, baseline HbA1c) of each placebo and in-sample lifestyle-intervention subject were normalized using a z-score. This resulted in *δ*_*i*_, the z-scored lifestyle-intervention subject attributes, and $\delta_{i}^{*}$ the z-scored placebo subject attributes. Using the normalized attributes of the two populations, the Euclidian distances between lifestyle-intervention and placebo subjects, $d=\sqrt{\sum_{i}w_{i}{\left(\delta_{i}-\delta_{i}^{*}\right)}^{2}}$, were measured, where *w*_*i*_ were empirically derived weights for the different attributes. The nearest neighbor of the placebo subject was defined as the in-sample lifestyle-intervention subject with the minimum value of *d*.

## Results

### Dynamics of the baseline T2D model

**Fig 2:**
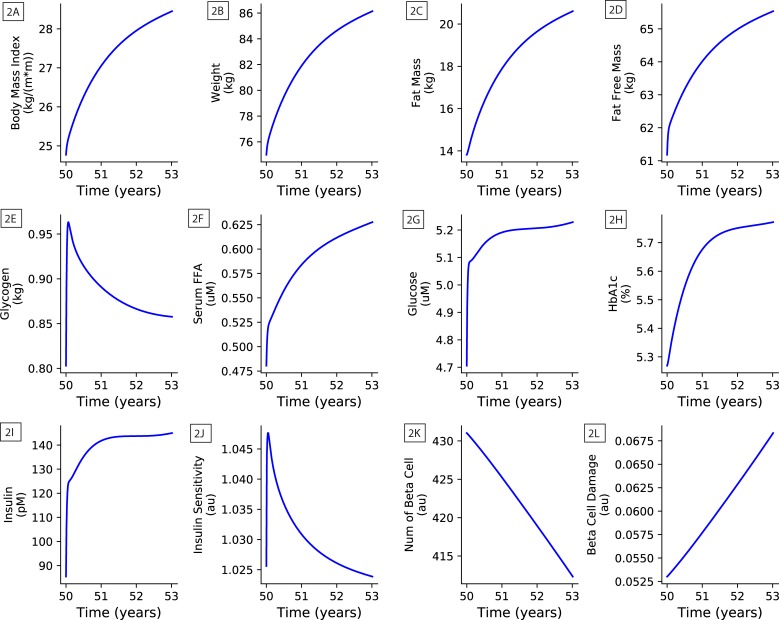
Baseline 3 year model simulation under increased diet intervention. **2A**: Body mass index (BMI); **2B**: Weight; **2C**: Fat mass; **2D**: Fat free mass; **2E**: Glycogen; **2F**: Serum free fatty acid (FFA); **2G**: Glucose; **2H**: HbA1c; **2I**: Insulin; **2J**: Insulin sensitivity; **2K**: Number of beta cells; **2L**: Beta cell damage.

To illustrate the dynamics of some representative variables of the model, the baseline model, not calibrated to any of the individual DPP subjects, was run for three years under the conditions of an increased diet intervention. The baseline diet, prior to the start of the simulation, was 161g/day of carbohydrates, 39g/day of fat, and 75g/day of protein (approximately 1300 kCal/day with caloric ratios of 50% carbohydrates, 30% fats, and 20 protein). The baseline model was simulated from age 20 to age 50 with the baseline diet as described in Methods: Numerical solution of model equations. At age 50 a step-function diet change was introduced that switched to a diet with increased carbohydrates by 20% (194g/day), increased fat by 10% (43g/day), and held protein at baseline levels (75g/day). The baseline model was then simulated for three years to age 53. The results of the baseline simulation are plotted in Fig 2.

In addition to weight (Fig 2B), HbA1c (Fig 2H), glucose (Fig 2G), and insulin (Fig 2I), which are examined in detail in this study, the plots also include time courses of fat mass (Fig 2C), fat free mass (Fig 2D), glycogen (Fig 2E), serum free fatty acids, FFA, (Fig 2F), body mass index, BMI, (Fig 2A), insulin sensitivity (Fig 2J), number of beta cells (Fig 2K), and beta cell damage (Fig 2L) over the course of the three year simulation.

With the increased diet there is general response of increased biomarker levels expressed in the rising curves of BMI, weight, fat mass, and fat free mass. There is an initial spike in insulin sensitivity (IS) due to the increased insulin, that is quickly followed by a decline in IS as serum FFA starts increasing and inhibits insulin signaling. Within the glucose/HbA1c dynamics there is movement from healthy values into a pre-diabetic state towards the end of the simulation. In combination with the damage caused over time by ROS levels, increased deviation of glucose and FFA from their baseline levels increases gluco- and lipotoxicity to the pancreatic beta cells causing their numbers and activity to decrease.

### Training of T2D model to DPP placebo trial arm

**Fig 3:**
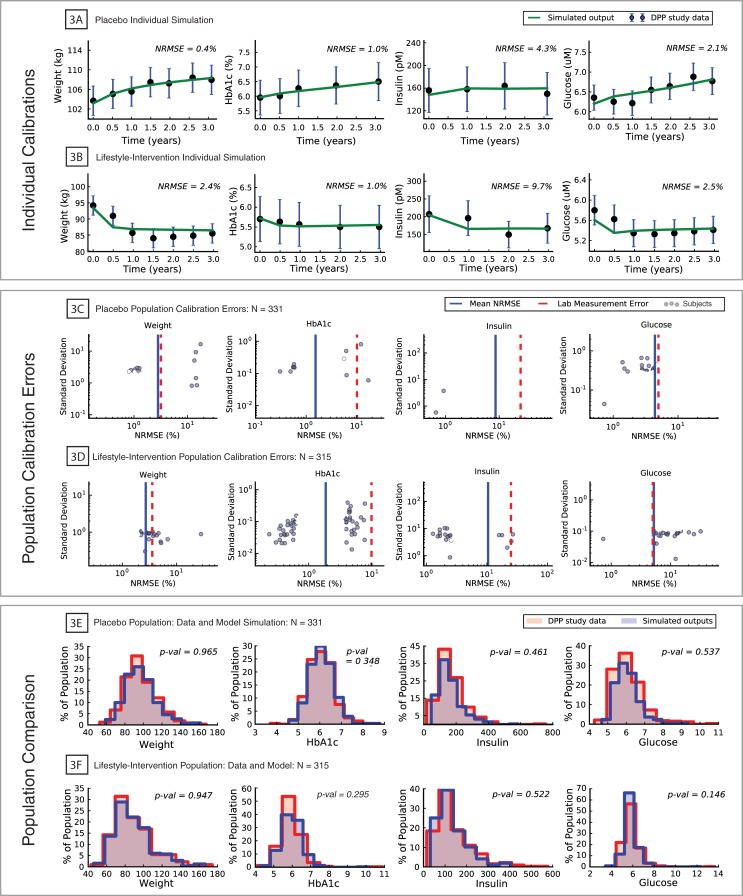
Results of model calibration to DPP placebo and lifestyle-intervention subjects. **3A**: Example of model calibration to individual placebo subject (data: black dots, standard measurement error: blue bars, model simulation: green line); **3B**: Example of model calibration to individual lifestyle-intervention subject (data: black dots, standard measurement error: blue bars, model simulation: green line); **3C**: Scatterplot of placebo population calibration errors (individual subjects: dots, population mean error: blue line, standard measurement error: red dash line); **3D**: Scatterplot of lifestyle-intervention population calibration errors (individual subjects: dots, population mean error: blue line, standard measurement error: red dash line); **3E**: Simulated placebo population (blue) compared to placebo arm population (pink) at year three of the DPP study; **3F**: Simulated lifestyle-intervention population (blue) compared to lifestyle-intervention arm population (pink) at year three of the DPP study.

The T2D model was calibrated separately to 331 study subjects as described in Methods: Individual model calibration. To compare the results of the calibration to the data, each subject was simulated from age 20 through to the completion of the third year of the study according to the Numerical solution model equations section in Methods. The results of the placebo arm calibration are plotted in Fig 3A, 3C and 3E.

An example individual placebo subject model simulation is plotted in Fig 3A, including the corresponding subject data, error bars representing standard clinical measurement error [120–123], and normalized root mean square error, NRMSE (see Methods: Individual model calibration). The model fit well to the data with NRMSE errors ranging from 0.4–4.3%, which were well in agreement within the clinical measurement errors. In general, of the four biomarkers presented, glucose and insulin had larger errors than weight and HbA1c. Variability of fasting glucose and insulin measurement are higher because the duration of fasting and amount of physical activity just prior to sample collection is not standardized in the clinical setting. Whereas. the temporal consistency of weight and HbA1c is much higher leading more stable measurements. Scatter plots of errors in each of the four biomarkers for the full placebo calibration population are shown in Fig 3C. In all four scatter plots, the mean population NRMSE of each biomarker was less than the corresponding clinical measurement error, indicating, at the population level, the model’s ability to reliably simulate the time courses of the biomarkers; quartiles of model errors of all biomarkers are listed in Table 6. For all four biomarkers the model error was weakly correlated with measurement error (Fig 3C). Distributions of the four biomarkers across all 331 placebo subject simulations after simulating the 3 years of the study were compared to the corresponding population data (Fig 3E). A 2-sample Kolmogorov-Smirnoff (K-S) test failed to reject, for all four biomarkers, the null hypothesis that the simulated placebo population was different from the DPP placebo population. Comparing the T2D prevalence at year three of the DPP study (defined as HbA1c ≥ 6.5%), the simulated placebo population had 80 T2D subjects (prevalence rate = 24.2%), and the DPP study had 73 T2D subjects (prevalence rate = 22.1%).

**Table 6:** Population calibration and forecast NRMSE errors.

DPP study arm	Biomarker	Calibration mean (NRMSE %)	Calibration 25^th^ percentile	Calibration 75^th^ percentile	Forecast mean (NRMSE %)	Forecast 25^th^ percentile	Forecast 75^th^ percentile
Placebo	Weight	2.80	1.40	3.34	4.38	1.44	5.94
	HbA1c	1.53	0.81	1.81	4.34	1.64	6.01
	Insulin	8.57	4.62	10.8	33.7	10.7	35.8
	Plasma glucose	4.41	2.32	5.53	6.82	2.32	10.3
Lifestyle-intervention	Weight	2.70	1.37	3.34	5.45	1.81	6.96
	HbA1c	1.87	0.97	2.31	3.86	1.40	5.24
	Insulin	10.5	5.46	13.3	36.8	9.39	45.9
	Plasma glucose	5.20	2.69	5.86	7.60	3.24	9.94

### Training of the T2D model to the DPP lifestyle-intervention arm

The T2D model was also calibrated to individual subject data from the lifestyle-intervention arm of the DPP study [124] using the same subject characteristic and biomarker time course data as in the placebo calibration (Table 1). As with the placebo group, the dietary intake of each subject in the lifestyle group was estimated by model calibration. The reported physical activity for the lifestyle subjects was found to have high intra-subject variability during the study period. To represent the additional physical activity of the lifestyle subjects in the model and reduce the variability in the reported activity, a linear regression was fit to each subject's reported physical activity over the course of the 3-year period and was used as a continuously varying representation of physical activity in the model. Only lifestyle subjects who met the data requirements (Method: Data processing) and whose physical activity regression fits had an *R*^2^ > 0.85 were selected (462 subjects) for calibration/prediction. An additional number of the lifestyle subjects (147 subjects) were excluded from the analysis that had an initial decrease in weight and HbA1c but eventual rise that, within the construction of the model, were physiologically inconsistent with the reported constant diet during the study.

Results of the lifestyle-intervention arm calibration are presented in Fig 3B, 3D and 3F. An example lifestyle subject (Fig 3B) lost approximately 10% of their starting weight and had reductions in their other three biomarkers with NRMSEs in the range of 1.0–9.5%. Scatter plots of the errors for each individual lifestyle subject are shown in Fig 3D. For the lifestyle-intervention arm, the mean errors were either less than or nearly equal to the standard measurement errors [120–123] for each of the four biomarkers (Fig 3D); quartiles of biomarker errors are listed in Table 6. Similar to the placebo errors, the lifestyle arm errors show a trend towards increase in error as the SD of the data increased (Fig 3D). As with the placebo population, K-S tests of model simulations versus the data from the four biomarkers failed to reject the null hypothesis that the model generated biomarker distributions of lifestyle subject were different from DPP distributions (Fig 3F).

### Sensitivity analysis of the T2D model

Sensitivities of weight, HbA1c, glucose, and insulin, output by the model, to the model parameters were estimated for each placebo subject (S1 Text), the median values across all subjects were chosen as a point estimate of the sensitivity and are plotted S7 Fig. It was found HbA1c is sensitive to changes in $Cmax_{hba1c}^{BLD}$ [S = 6.58E-2] whereas serum glucose is rather insensitive to it [S = 2.18E-4]. There was a strong sensitivity of weight to changes in fat intake during the study [S = 5.97E-2] consistent with the coaching the DPP subjects received to reduce fat intake during the study. The sensitivity of insulin to carbohydrate intake before the study [S = 4.19E-2] highlights the need to account for subject history in addition to their current state. Also there was a strong sensitivity of glucose to the half-maximal concentration of glucose for insulin synthesis [S = 1.33E-1] while insulin was not as sensitive to the same parameter [S = 2.32E-2]. In prediabetic subjects serum glucose begins to rise due to increase of insulin resistance and, as described in S7 Table, it is expected that serum glucose would be sensitive to changes in α_*dep_ffa*_ which directly increases insulin resistance. Yet it is observed from our analysis that serum glucose is more sensitive to the *k*_*dep_ffa*_ rather than α_*dep_ffa*_.

Confidence in parameter estimates was tested by computing the standard deviation and coefficients of variation (S1 Text and S12 Table). The median CV for each of the parameters across all the placebo subjects is given in S12 Table. All the parameters had median CVs below 20% which provides significant confidence on the estimation process. Further, based on the covariance matrix, the correlation matrix was also computed for each subject and the median correlation matrix was analyzed. No significant cross-correlations was observed between the parameters estimated [-0.13, 0.08].

### Prediction of lifestyle-intervention

**Fig 4:**
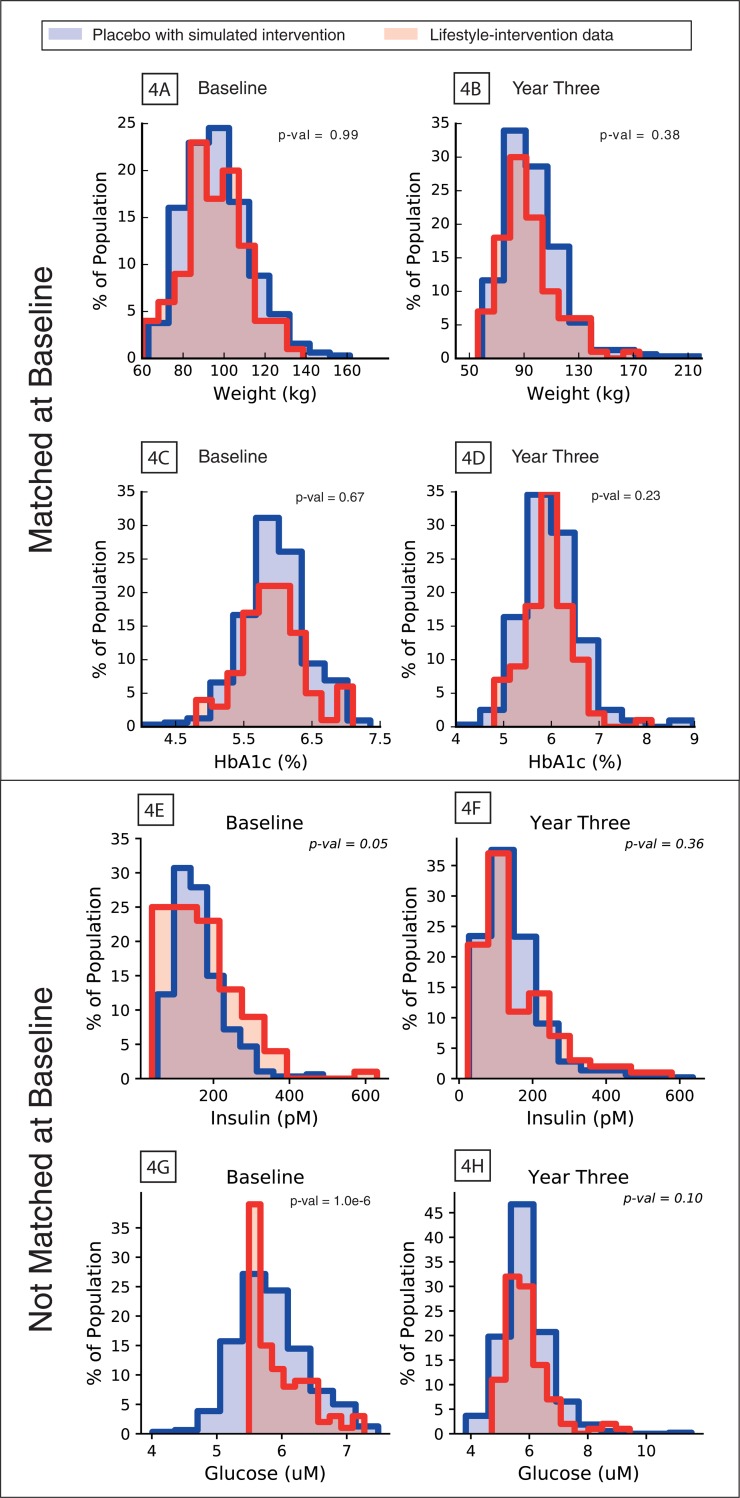
Comparison of simulated placebo intervention and observed lifestyle-intervention groups. **4A**: Simulated placebo intervention (blue) and lifestyle-intervention (pink) populations weight at DPP study baseline; **4B**: Simulated placebo intervention (blue) and lifestyle-intervention (pink) populations weight at DPP study year three; **4C**: Simulated placebo intervention (blue) and lifestyle-intervention (pink) populations HbA1c at DPP study baseline; **4D**: Simulated placebo intervention (blue) and lifestyle-intervention (pink) populations HbA1c at DPP study year three.

One goal of this study was to predict the effect of lifestyle interventions on pre-diabetic subjects based on the time course of weight and HbA1c measurements. The DPP lifestyle-intervention arm was not designed with a specific uniform intervention for all subjects, but was broadly defined as aiming to achieve a 7% weight reduction through diet change and moderate activity, such as brisk walking for at least 150mins per week. As a result, every subject in the study had a unique intervention. It was observed, at the start of the study, the baseline distributions of age, weight and HbA1c of placebo subjects when compared to those of subjects in the lifestyle-intervention arm were significantly different (K-S test: p<0.05). This necessitated a sub-sample of subjects (n = 200) from the lifestyle-intervention subjects be chosen whose population baseline measurements were statistically similar to those of the placebo subjects. The subsample was chosen such that the resulting population’s baseline distributions of weight and HbA1c and were not significantly different between the placebo and lifestyle groups (Fig 4A and 4C; K-S test: p>0.05). To test the simulated placebo interventions, the sub-sampled lifestyle group was randomly divided into equal size (100 each) in-sample and out-of-sample groups. To validate the model's ability to predict the effects of lifestyle interventions, each placebo subject was assigned a unique lifestyle-intervention based on their nearest neighbor from the in-sample lifestyle-intervention group (as described in Methods: Nearest neighbor estimation). The change in physical activity (intercept and slope) and change in intake of carbohydrate and fat, estimated from model calibration, of the lifestyle nearest neighbor was assigned to the placebo subject. Each placebo subject was then simulated for the three years of the study using the assigned lifestyle intervention and the aggregate population of model outputs were compared to the data from the out-of-sample lifestyle-intervention group.

The simulated placebo population with interventions and the out-of-sample lifestyle-intervention were compared at year three (Fig 4B and 4D). The p-values indicate the K-S tests failed to find a statistical difference between these two groups in their distributions of weight and HbA1c at year three. This indicates the model was able to predict the effect of the lifestyle-intervention arm by applying the intervention to the placebo arm. This is valuable because it demonstrates the model's capability of being used to computationally explore the effect of different interventions on a specified population.

We also included the population distributions of glucose and insulin in Fig 4. The population was not matched on glucose and insulin at baseline because of higher variability in these measurements as discussed in section Result: Training of T2D model to DPP placebo trial arm. To be eligible to participate in the DPP study the subject's glucose had to greater than 5.3 mmol/L, this generated the hard cutoff seen in the baseline glucose population. In running the model for the lifestyle-intervention prediction glucose and insulin are generated as outputs and their dynamics are of interest in assessing the prediction. Even though the populations' glucose and insulin did not match well at baseline, at year three they found to not be significantly different (three year p-val: glucose = 0.10, insulin = 0.36). This indicates that the internal homeostasis or physiological consistency of the model insured that the population glucose and insulin matched even though they were not explicitly accounted for at baseline.

#### Forecasting of individual subjects

**Fig 5:**
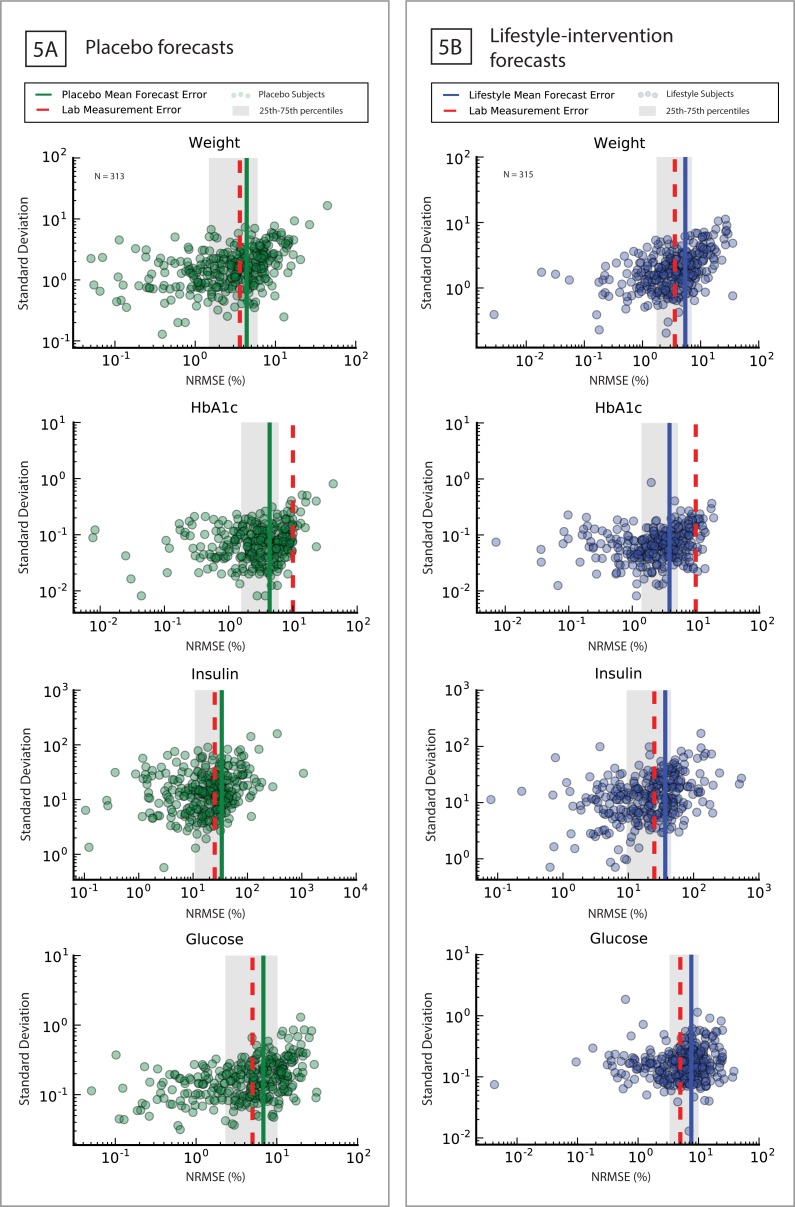
One year forecasts of placebo and lifestyle-intervention arm subjects. **5A**: Forecast of placebo year three clinical data (individual errors: green dots, mean population error: green line, standard measurement error (red dashed line); **5B**: Forecast of lifestyle-intervention year three clinical data (individual errors: green dots, mean population error: green line, standard measurement error (red dashed line).

Having shown the T2D model can be calibrated to both placebo and lifestyle subjects, we investigated the model's ability to do one year forecasts of the four biomarkers outputs. The forecasts were constructed by withholding the last year of the three years of data used in the Results: Placebo calibration. Each subject in the placebo and lifestyle-intervention arms were fitted with only the first two years of data, with fitted models containing no information about the data from the third year of the study. Having calibrated the model to the first two years, trajectories of biomarkers in the third year were predicted by simulating the intervention during year three (Fig 5).

The mean forecasting errors for this group were 4.4% weight, 4.3% HbA1c, 33.7% insulin, 6.8% plasma glucose (Fig 5A); population mean errors, and 25^th^ and 75^th^ percentiles for the biomarkers are listed in Table 6. All forecasting errors for the placebo group were within the same order of magnitude as the measurement error, with the HbA1c mean forecast error being less than standard measurement error. The mean forecasting errors for the lifestyle-intervention group were 5.5% weight, 3.9% HbA1c, 36.8% insulin, 7.6% plasma glucose (Fig 5B). The mean forecast errors for the lifestyle arm were nearly identical to those of the placebo group. This similarity demonstrates the T2D model's flexibility in predicting, with similar accuracy, people with their historical diet and physical activity and those that abruptly shifted to a new lifestyle that forced a change in their health trajectory.

#### Role of endogenous and exogenous factors in diabetes progression

**Fig 6:**
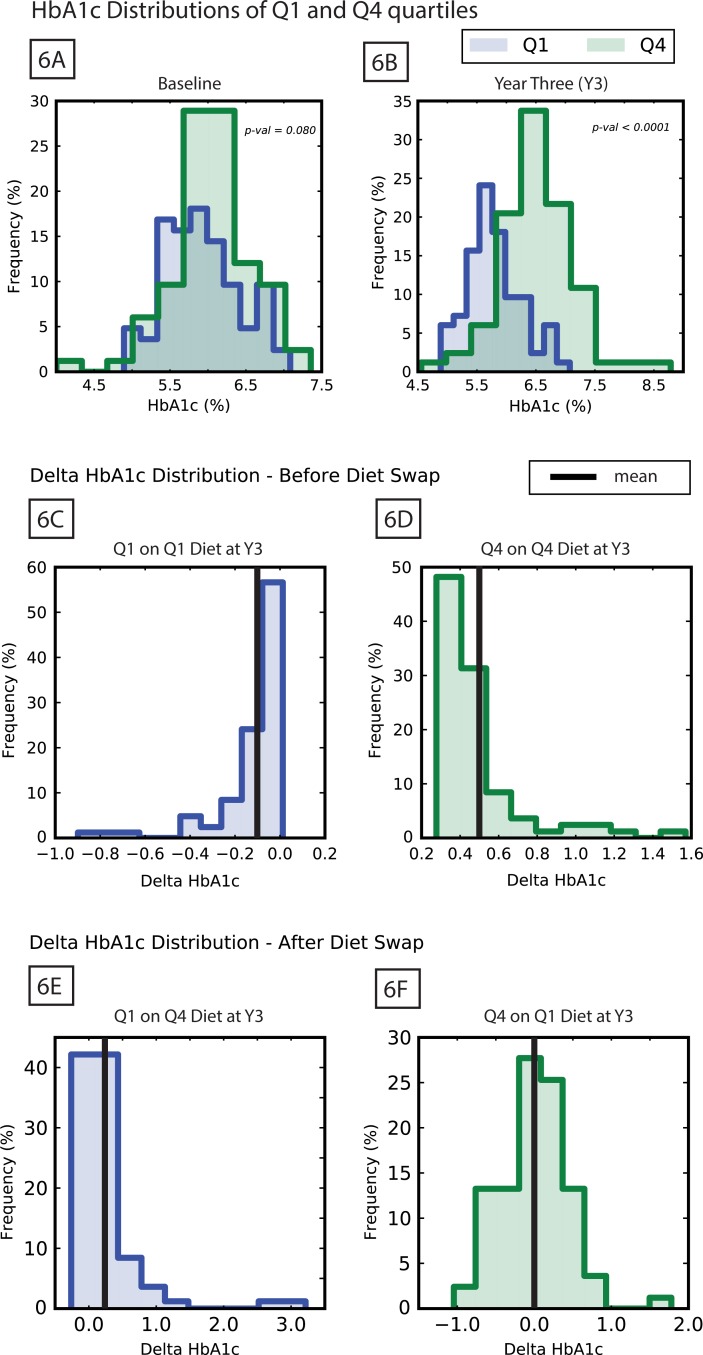
Results of the computational experiment on DPP placebo first (Q1) and fourth (Q4) quartiles of change in HbA1c over first three years. **6A**: Distributions of HbA1c for Q1 (blue) and Q4 (green) at DPP baseline study start; **6B**: Distributions of HbA1c for Q1 (blue) and Q4 (green) at year three of the DPP study; **6C**: Distribution of change in HbA1c of Q1 subjects data at year three of the DPP study; **6D**: Distribution of change in HbA1c of Q4 subjects data at year three of the DPP study; **6E**: Distribution of change in HbA1c of Q1 subjects simulated with the mean Q4 diet at year three of the DPP study; **6F**: Distribution of change in HbA1c of Q4 subjects simulated with the mean Q1 diet at year three of the DPP study.

All subjects in the placebo arm were similar at baseline by design of the original DPP study [124]. By the end of the three years examined here, the values of HbA1c for individuals in this group had dispersed. We ranked the subjects by their change in HbA1c values over the three years of the study and compared the individuals in the first quartile (Q1), whose HbA1c remained steady or decreased, to those in the fourth quartile (Q4), whose HbA1c rose the most, indicating progression/onset of diabetes (Fig 6A and 6B). K-S tests of the HbA1c distributions of these two groups show at baseline the Q1 and Q4 populations were not significantly different (p-value = 0.08) but at year three they had diverged (p-value < 1e-4). The fitting of the T2D model to the individual subjects in both the Q1 and Q4 populations (Results: Placebo Calibration) resulted in a collection of parameters, for each individual, that describes the subjects' exogenous and endogenous characteristics (Methods: Individual model calibration). We used this collection of parameters to investigate the differences between the subjects in the Q1 and Q4 quartiles. To compare these groups, we independently tested the distributions of fitted parameters in Q1 and Q4 for each of the 12 model parameters using a K-S test (Table 7).

**Table 7:** Mean values of optimized parameters in groups Q1 and Q4.

Parameter abbreviation	Parameter type	Q1 parameter values	Q2 parameter values	K-S test p-values
*CI*_0_	exogenous	111.2	122.1	0.239
*FI*_0_	exogenous	42.65	38.43	0.433
*CI*/*CI*_0_	exogenous	1.512	1.444	0.0214
*FI*/*FI*_0_	exogenous	1.166	1.337	5.14e-5
*CI*_2_/*CI*_0_	exogenous	1.273	1.498	1.14e-5
*FI*_2_/*FI*_0_	exogenous	1.683	1.050	1.76e-12
$Cmax_{hba1c}^{BLD}$	endogenous	27.43	39.11	1.44e-3
$C_{hba1c}^{BLD}$	endogenous	4.613	4.503	0.816
*α*_*dep_ffa*_	endogenous	15.17	12.96	0.556
*k*_*dep_ffa*_	endogenous	10.95	18.18	0.326
*α*_*bc*,*s_ros*_	endogenous	5.821	12.68	2.28e-6
*KM*_*s*,*ins_glu*_	endogenous	13.83	9.829	0.0214

The parameters found to be significantly different between the Q1 and Q4 groups were the carbohydrate and fat consumption before (*CI*/*CI*_0_ and *FI*/*FI*_0_) and during (*CI*_2_/*CI*_0_ and *FI*_2_/*FI*_0_) the study, two parameters related to the dynamics of the pancreas in response to chronic hyperglycemia (*α*_*bc*,*s_ros*_ and *KM*_*s*,*ins_glu*_), and $Cmax_{hba1c}^{BLD}$, the maximal serum HbA1c concentration. It is unsurprising and reassuring that within the structure of the model the diets of these two groups were different as this is accepted as a major driver of the progression of pre-diabetes into diabetes. The basal metabolisms (*CI*_0_ and *FI*_0_) between Q1 and Q4 were not found to be different, indicating these groups had similar baseline metabolic demands making the differences in diets a direct exogenous driver. Of the endogenous parameters that were significantly different, the difference in maximal serum HbA1c concentration indicates the relationship between plasma glucose and HbA1c were different between Q1 and Q4. The two pancreatic parameters indicate Q1 and Q4 had different sensitivities to reduction in capacity of insulin production in response to chronic hyperglycemia, while the parameters related to insulin resistance were not different.

To measure the relative contributions of exogenous diet versus the endogenous reduction of insulin production to observed changes in HbA1c, we conducted a computational experiment on the Q1 and Q4 groups. To examine the influence of the endogenous factors, the mean Q1 diet was fed to the Q4 subjects, and the mean Q4 diet was fed to the Q1 subjects and simulated for the 3 years of the simulated DPP experiment using the placebo arm calibrations of the Q1 and Q4 subjects. The results of this computational experiment are shown in Fig 6C–6F. In the Q1 group, all subjects’ HbA1c values either decreased or remained the same with a mean delta HbA1c of -0.118 (Fig 6C). In the Q4 group, all subjects' HbA1c increased with a mean delta HbA1c of 0.470 (Fig 6D). Feeding the Q4 diet to Q1 subjects caused nearly all Q1 subjects' HbA1c to rise over 3 years with a mean delta HbA1c of 0.264 (Fig 6E). Feeding the Q1 diet to Q4 subjects caused some Q4 subjects' HbA1c to drop over the 3 years while some still increased (Fig 6F); the mean delta HbA1c for this group was 0.002. The change in HbA1c from Q1 on the Q1 diet (Fig 6C) to Q1 on the Q4 diet (Fig 6E) was 0.382, and the change in HbA1c from Q4 on the Q1 diet (Fig 6D) to Q4 on the Q4 diet (Fig 6F) was 0.468.

The larger increase in HbA1c in the Q4 group in response to the diet switch implies that people in Q4 have endogenous characteristics that increased their propensity towards higher HbA1c levels as compared to subgroup Q1. If shifts in HbA1c were purely driven by exogenous factors (i.e., diet), we would expect the difference in HbA1c on the switched diets to be almost identical for Q1 and Q4. However, the shift for Q4 (0.468) is ~23% higher than that for Q1 (0.382), implying that endogenous traits of Q4 act together with exogenous factors resulting in an increase that is higher than what would be expected based only on diet change. It is worth noting that while endogenous traits affected HbA1c levels, their contribution was much smaller than that of diet change. This is in line with the current view that lifestyle has a greater role to play in diabetes progression than genetic factors [125].

## Discussion

In this study we have described, calibrated and validated a model of the physiological mechanisms of prediabetes development and diabetes onset in an individual. Our model simulates the dynamics of diabetes progression by mechanistically modeling the complex interactions between the following diabetes-related metabolic processes: the dynamics of pancreatic insulin production by beta-cells under normoglycemia and chronic hyperglycemia; pancreatic decompensation by cumulative damage to pancreatic beta cells due to glucotoxicity, and lipotoxicity; development of insulin resistance as a consequence of inhibition of insulin signaling by FFA and ROS; expression of insulin resistance through inhibition of translocation of GLUT1 and GLUT4 receptors in response to plasma insulin; energy production by oxidation of glucose, FFA, and amino acids; glycogenesis; glycogenolysis; lipolysis; de novo lipogenesis; proteolysis; protein synthesis; ketogenesis; gluconeogenesis; dynamics of ATP production and conversion to ADP as function of metabolism and energy expenditure; and hemoglobin glycosylation. Critically, the model is fit at the individual level and requires only common clinical measurements which allows for its translational use in clinical and public health applications.

We have demonstrated the model's capabilities to simulate and retrospectively forecast longitudinal data collected from pre-diabetic subjects in the placebo and lifestyle-intervention arms of the Diabetes Prevention Program (DPP). The calibration of the model to the two arms of the DPP study consisted of fitting separate instances of the model to three years of longitudinal data to each of 646 study individuals. The population average error of the simulated biomarkers of the fitted models over three years, compared to the DPP data, was less than standard measurement error for weight, HbA1c, fasting insulin, and nearly equal to measurement error for fasting plasma glucose for both the placebo and lifestyle-intervention arms. We found the distributions of the four simulated biomarkers at year three of the study were not significantly different than the corresponding distributions of DPP data. Further, the forecasting error of the model was tested by withholding the third year of study data, fitting model to only the first two years of data, and predicting the third year data. The resulting one year forecast errors were less than or the same order of magnitude as measurement error with population average errors less than 10% for weight, HbA1c, and glucose for both the placebo and lifestyle-intervention arm subjects.

In addition to capturing population level distributions, the model does well at reproducing the non-linear relationships between the dynamic responses of biomarkers at the individual subject level. For example, the model was able to capture the increase in weight, HbA1c and plasma glucose levels even while insulin levels remained relatively constant (Fig 3A). This suggests that the pancreas' ability to generate insulin had likely been compromised due to pancreatic beta-cell damage. This is a dominant feature of T2D progression and the model was able to recreate this for the particular subject simulated in Fig 3A. In contrast, the subject simulated in Fig 3B shows a reduction in multiple biomarkers including body weight and HbA1c, which the model reproduced correctly. This example shows the model's ability to represent a pre-diabetic subject's movement back towards a healthier state. This is significant because it demonstrates the model does not deterministically force subjects to progress to a diabetic state but is also capable of representing healthy metabolisms.

While the model fit well to most individuals, there was a subset of individual dynamics which the model failed to capture (Fig 3C and 3D). This can be attributed to assumptions made in the model as well as quality of inputs available to inform the model. As described in the Model Structure section, we made several simplifying assumptions in the model, e.g., we have ignored the counter-regulatory hormones of insulin, which could partly be the reason for the model’s failure to fit to a large number of insulin data points within measurement error. Additionally, the data available to inform the model about the subjects’ lifestyle were also quite limited. For example, we had to assume each subject was on an identical diet throughout the study period of 3 years, which in many cases is not a reasonable assumption. This could have contributed to the model’s failure to capture some trends in body weight and other variables. It is encouraging that despite the severe limitations of precise inputs to the model, a large majority of data points were appropriately reproduced and predicted by the model. We also limited the number of parameters that were varied on an individual basis to 12, as described in Methods: Individual model calibration. It is possible that using more parameters to calibrate to individual data would have improved the fits.

Having established the model's skill at simulating the dynamics of the placebo and lifestyle-intervention arm DPP subjects, we investigated its ability to predict the effects of a lifestyle intervention on placebo subjects. The diet and exercise schedules of a random subsample of lifestyle-intervention subjects was computationally applied to placebo subjects with similar biomarkers at the start of the study and simulated over three years of the study. A comparison of the population of placebo subjects that had undergone a computational lifestyle-intervention to the out-of-sample lifestyle-intervention subjects’ data were not found to be significantly different over the four simulated biomarkers.

Finally, we conducted a computational experiment by examining the placebo subjects whose HbA1c increased the most (Q4) and those who’s changed the least (Q1) over the three years of the study. We found these two groups had significantly different diets both before and during the study but did not have different baseline metabolisms. By analyzing parameters fit to these subjects, we found they had significant differences in the values of some parameters; for instance, the pancreatic insulin production characteristics differed significantly between the groups even while insulin resistance progression sensitivities were similar. This indicates there could be endogenous differences, possibly genetic, between people who progressed toward becoming diabetic in comparison to similar subjects who did not, in the dynamics of their insulin production but not in their insulin sensitivity. This is consistent with genome-wide association studies (GWAS) that have found genetic drivers of diabetes that effect insulin production but not insulin resistance [125]. To test the contribution of these exogenous and endogenous factors to the observed Q4 group's increase in HbA1c, we conducted a computational experiment that switched the average diets of these two groups and simulated them for the three years of the DPP study. The results indicated that endogenous factors led to a 23% increase in Q4 group's HbA1c over the Q1 group during the three years of the DPP study data analyzed here.

While our model builds significantly on previous models, there are several limitations that need to be addressed in future iterations. The metabolic fluxes of nutrients are abstracted at the organ level, so cellular level regulation of fluxes through various anabolic and catabolic pathways are not simulated for the sake of parsimony. This limits the model’s ability to represent phenomena such as nutrient selection and regulation by subcellular enzymatic processes with mechanistic accuracy. The adaptation of energy metabolism to exercise by reducing the perturbation of homeostasis in response to similar levels of physical activity is not represented in the model [126,127]. The modification of protein synthesis and breakdown rates by resistance training [128] are not represented in the model. Additionally, sarcopenia, the loss of muscle mass as a consequence of aging is also not simulated in the model. These protein-related processes are important because of the important role played by skeletal muscle mass in resting energy expenditure [129]. Our model does not explicitly represent glucagon and the net effect of the balance between glucagon and insulin is abstracted into insulin dynamics. Furthermore, the multiple steps involved in the synthesis of the insulin peptide are abstracted into a single step. Of the various molecular mechanisms thought to be involved in development of insulin resistance, we chose to include the effects of FFA, ROS mediated inhibition of insulin signaling, and degradation of mitochondrial function [53,130–132]. The role of other mechanisms such as adipokines, dynamic regulation of phosphatase and kinase activity were not represented [133]. All of these assumptions and approximations are likely to impact the outcomes of the simulations and could at least in part be the reason for the inability of the model to fit to or predict the data of some individuals in our analysis.

The model described here may also be useful in broader public health scenarios for studying populations at risk for developing diabetes. The model could be used to computationally test proposed lifestyle interventions or, in an optimization framework, search for an optimal intervention that would maximize a population's change in health given the limitations of interventions under consideration. These translational uses of the model were intended during its development and are possible because the data requirements are commonly tracked clinical biomarkers. Because the model represents health trajectories at the individual level, it could potentially be used by pre-diabetic subjects to synthesize their clinical measurements with activity and dietary data they collect through wearable and mobile devices to generate a more holistic view of how their lifestyle guides their risk of diabetes at a personalized level. In this scenario, the model could be fit to the individual subject as new clinical and wearable data are collected. When used in this manner, a subject could conduct computational experiments on themselves to see how their health may change in the future to aid and motivate making decisions about implementing changes in their lifestyle to improve their risk for diabetes. Future development of the model include expansion of the physiological scope to enable the study of comorbidities associated with diabetes, as described by metabolic syndrome, and the mechanistic modeling of the action of pharmaceutical drugs focused on metabolism to aid in clinical trial design and better understanding of the interaction between lifestyle and drug efficacy.

## Supporting information

S1 Fig Schematic diagram of blood component. (PDF)

S2 Fig Schematic diagram of muscle component. (PDF)

S3 Fig Schematic diagram of liver component. (PDF)

S4 Fig Schematic diagram of adipose component. (PDF)

S5 Fig Schematic diagram of pancreas component. (PDF)

S6 Fig Schematic diagram of insulin resistance component. (PDF)

S7 Fig Sensitivity of fitted model parameters to individual DPP subject clinical data. (PDF)

S8 Fig Fitness landscape of model parameters. (PDF)

S1 Table Model variables and notations. (PDF)

S2 Table Differential equations, expressions and variables of the blood compartment. (PDF)

S3 Table Differential equations, expressions and variables of the muscle compartment. (PDF)

S4 Table Differential equations, expressions and variables of the liver compartment. (PDF)

S5 Table Differential equations, expressions and variables of the adipose compartment. (PDF)

S6 Table Differential equations, expressions and variables of the pancreas compartment. (PDF)

S7 Table Differential equations, expressions and variables of the insulin resistance compartment. (PDF)

S8 Table Model parameters estimated using data from intracellular studies. (PDF)

S9 Table Model parameters estimated using data from healthy metabolic studies. (PDF)

S10 Table Model parameters estimated from diabetes related studies. (PDF)

S11 Table Initial conditions at age 20. (PDF)

S12 Table Median coefficient of variation of estimated parameters. (PDF)

S13 Table Correlation matrix of individually fit model parameters. (PDF)

S1 Text Model sensitivity analysis. (DOCX)
