# Supplementary figures and images for: A long-term mechanistic computational model of physiological factors driving the onset of type 2 diabetes in an individual
Source: PLoS One. 2018 Feb 14;13(2):e0192472. doi: 10.1371/journal.pone.0192472 (PMC5812629; doi:10.1371/journal.pone.0192472)

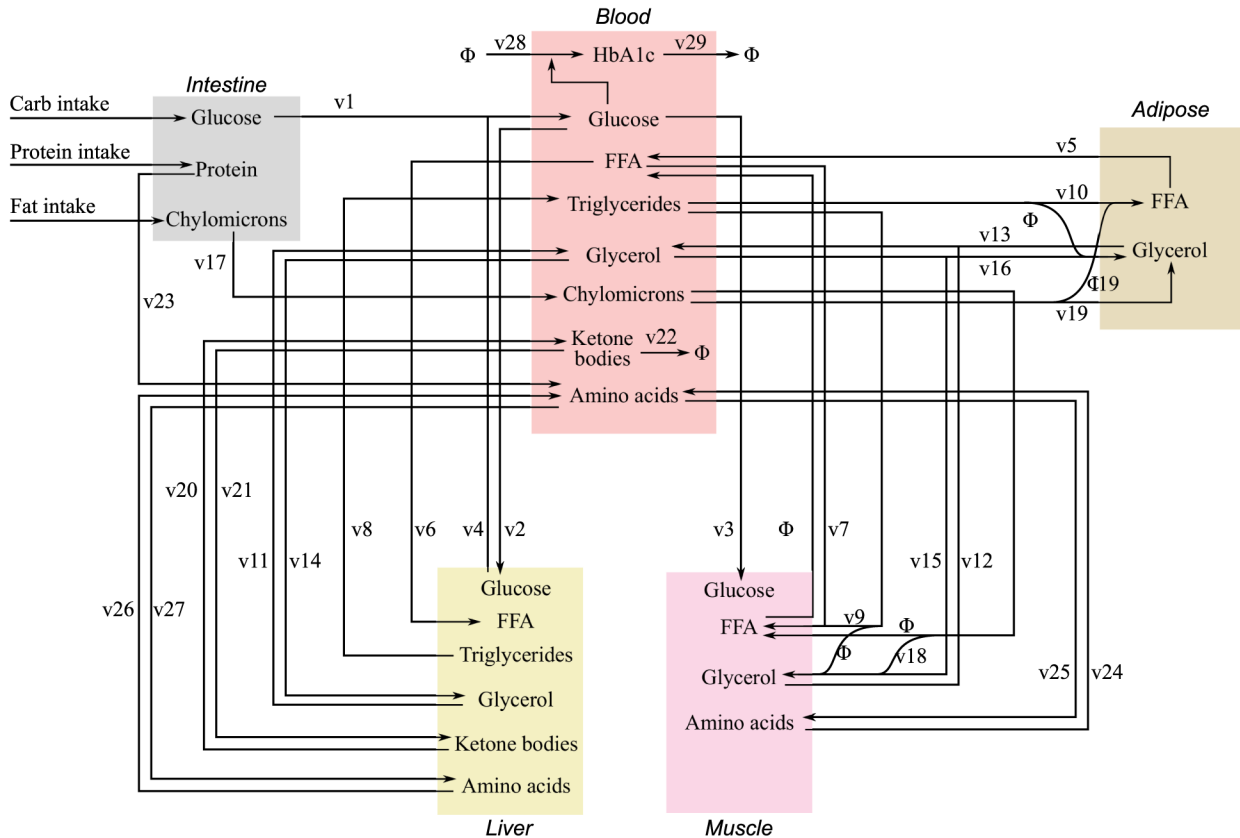

Supplement: S1 Fig — (PDF) [file pone.0192472.s001.pdf]

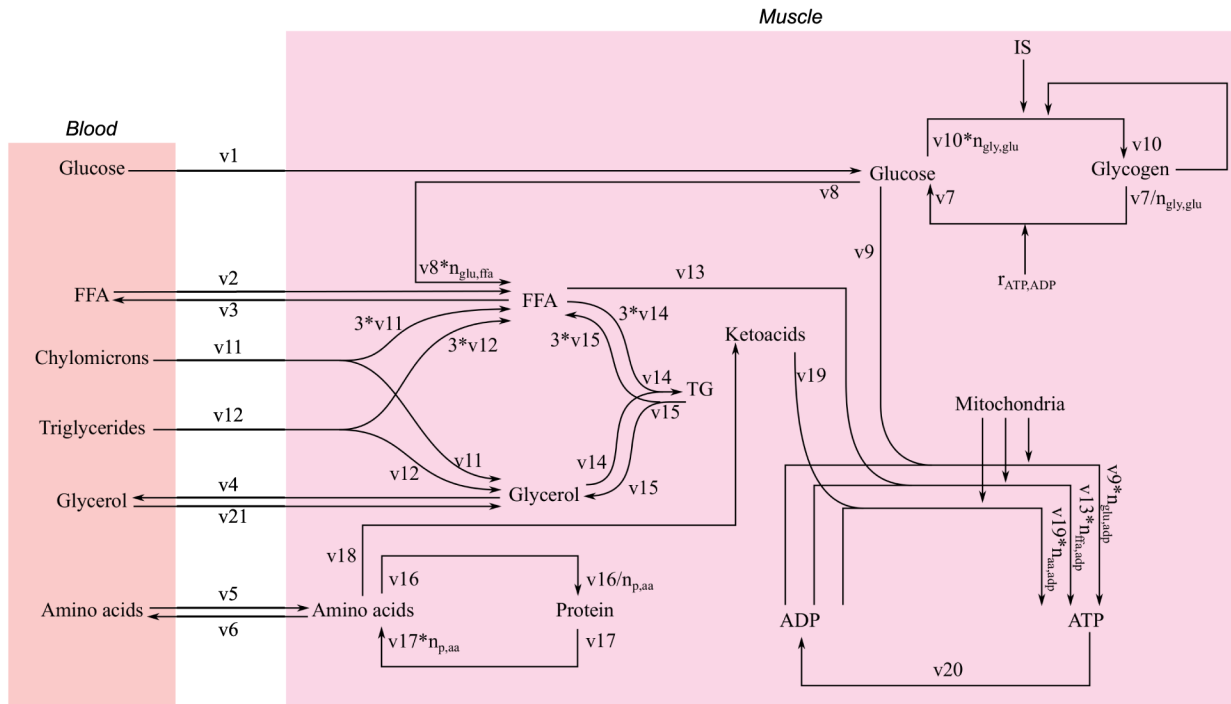

Supplement: S2 Fig — (PDF) [file pone.0192472.s002.pdf]

# Liver

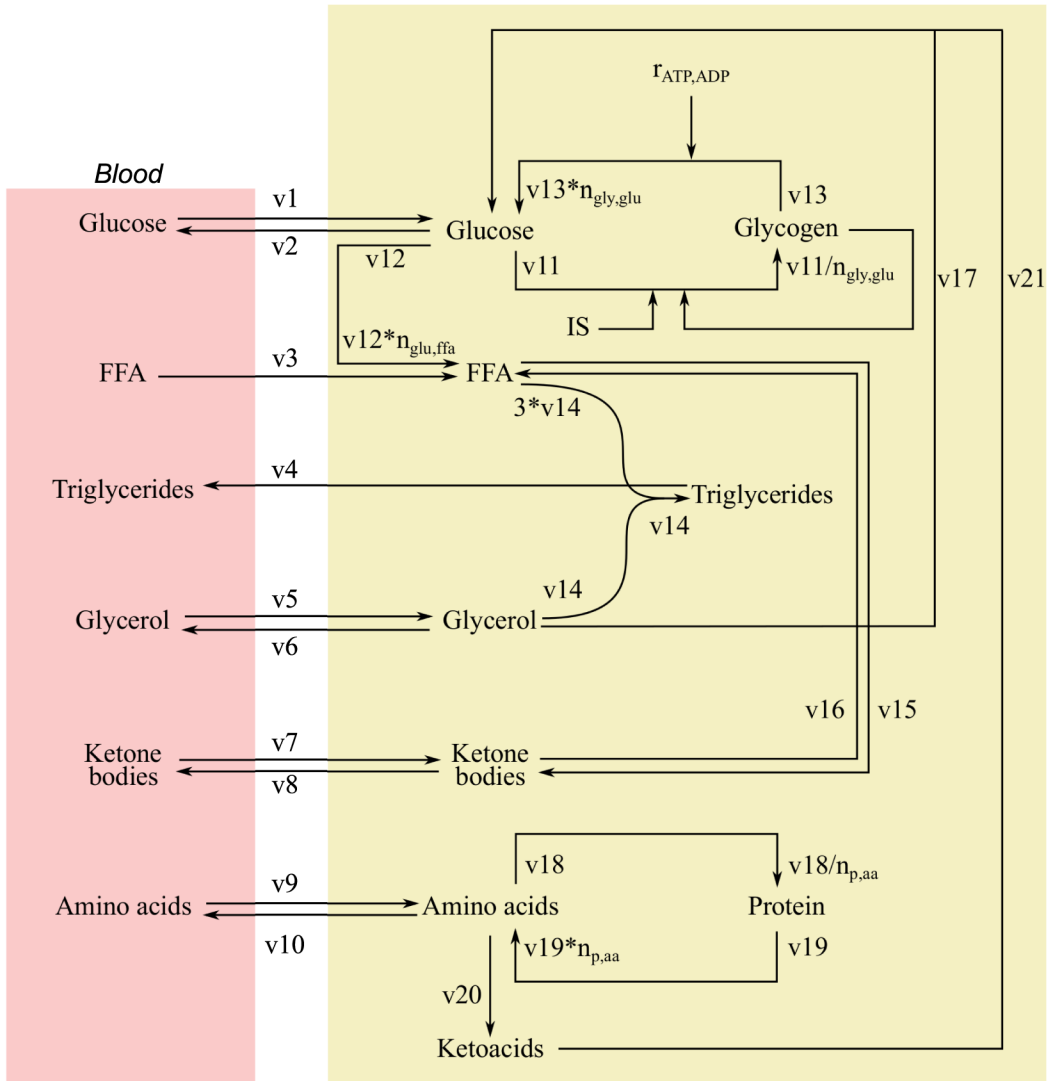

Supplement: S3 Fig — (PDF) [file pone.0192472.s003.pdf]

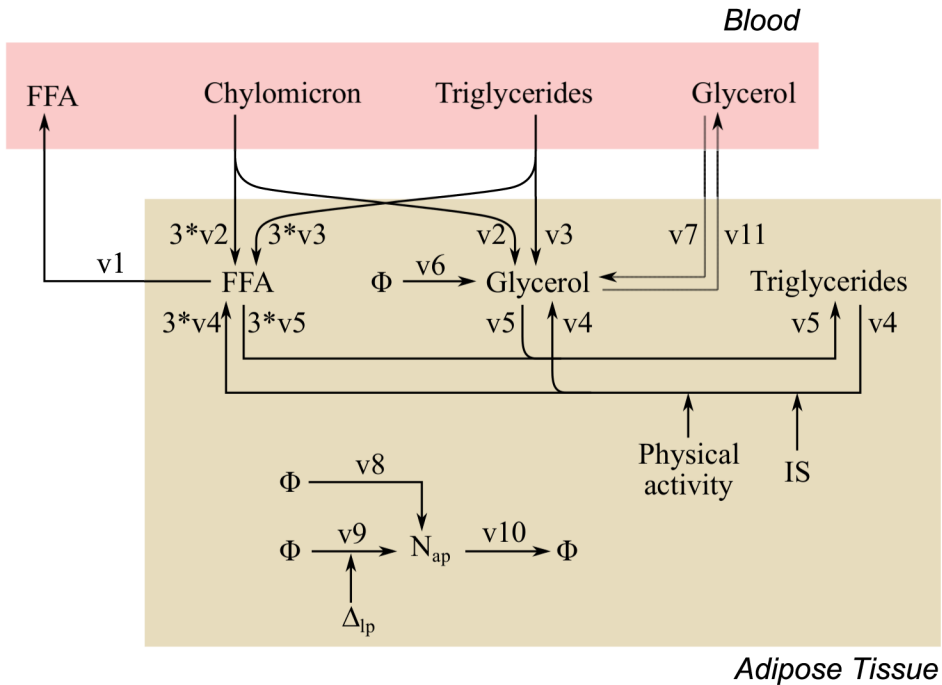

Supplement: S4 Fig — (PDF) [file pone.0192472.s004.pdf]

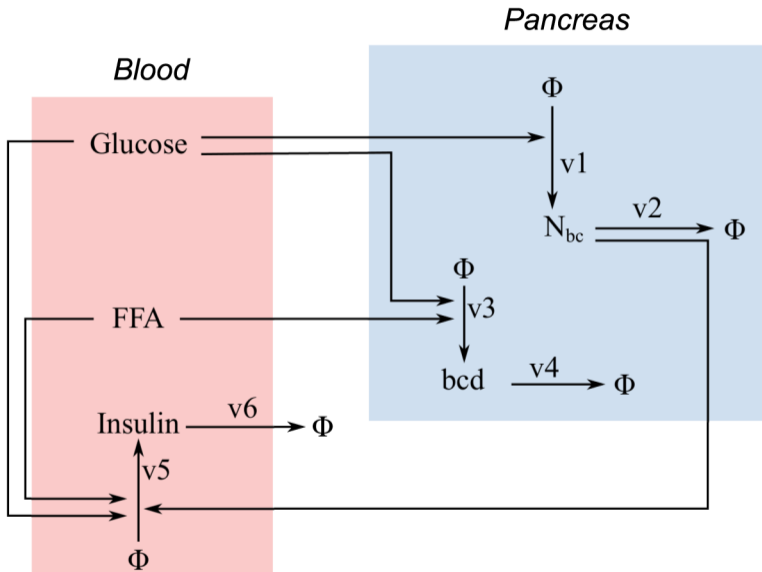

Supplement: S5 Fig — (PDF) [file pone.0192472.s005.pdf]

## Insulin Resistance

**A**

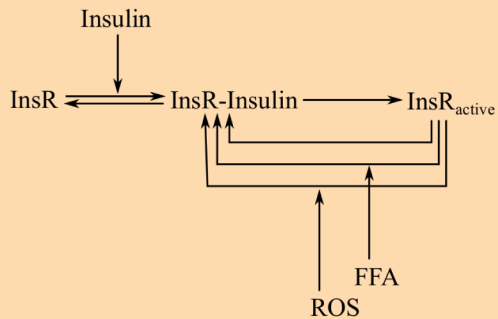

**B**

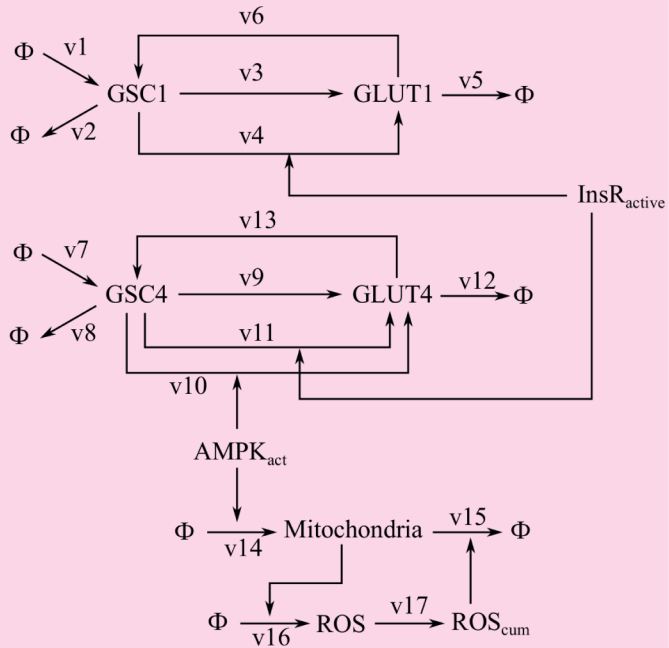

Supplement: S6 Fig — (PDF) [file pone.0192472.s006.pdf]

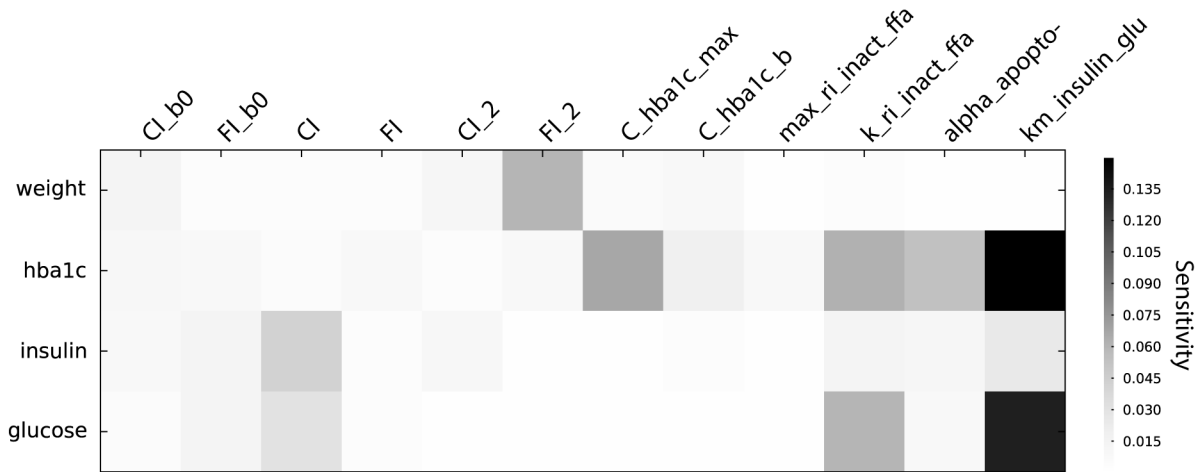

Supplement: S7 Fig — (PDF) [file pone.0192472.s007.pdf]

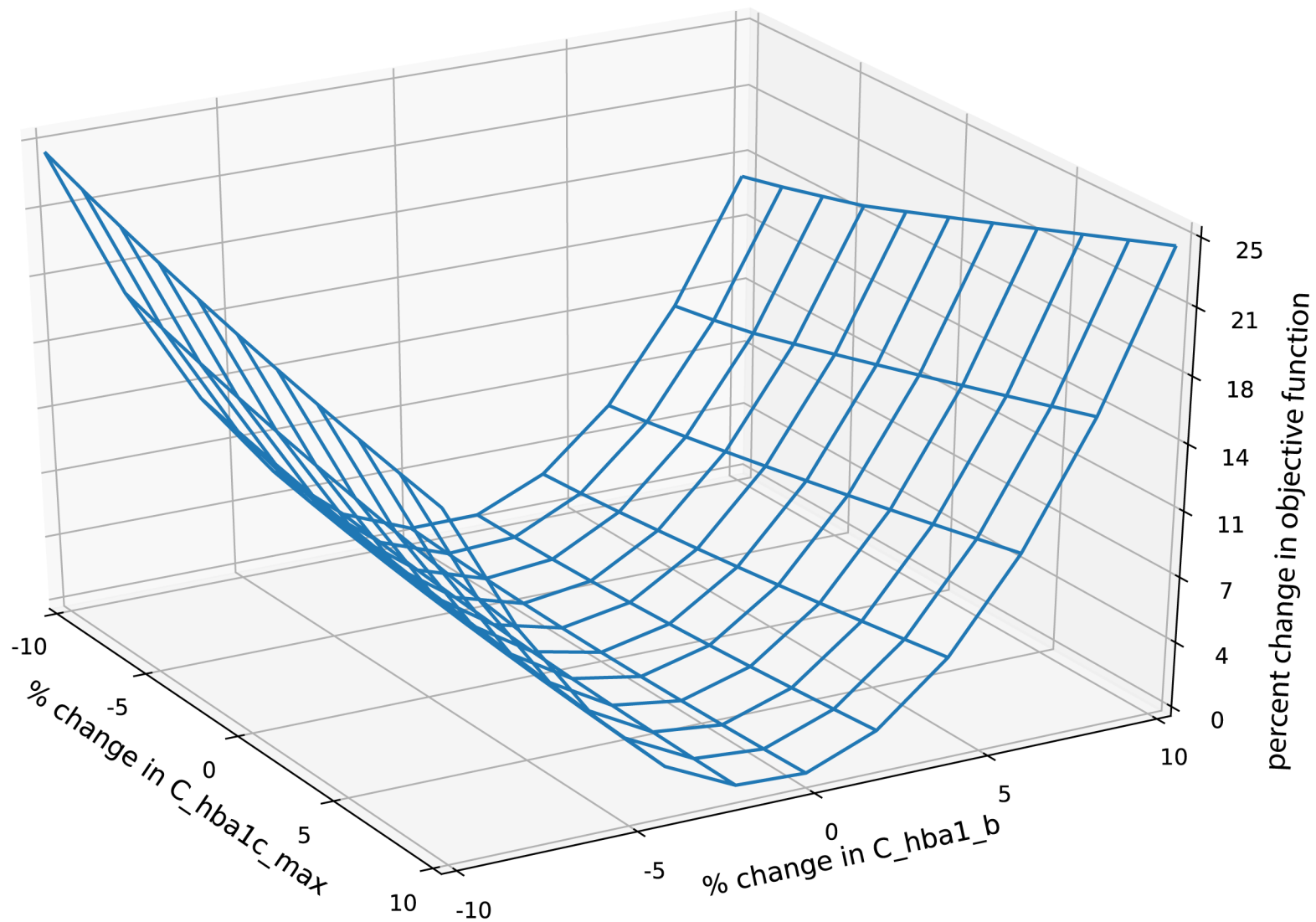

Supplement: S8 Fig — (PDF) [file pone.0192472.s008.pdf]
