# Supplementary material for: A long-term mechanistic computational model of physiological factors driving the onset of type 2 diabetes in an individual
Source: PLoS One. 2018 Feb 14;13(2):e0192472. doi: 10.1371/journal.pone.0192472 (PMC5812629; doi:10.1371/journal.pone.0192472)
Supplement: S1 Table — (PDF) [file pone.0192472.s009.pdf]

S1 Table. Model variables and notations.

S1.1 Table. General notation description.

Word/Phrase	Abbreviation
Steady state	_ss
Adipose	ADI
Adenosine monophosphate	AMP
AMPK activity	AMPK
Beta cell	bc
Damage to beta cell capacity	bcd
Blood	BLD
Chylomicrons	chy
Extracellular Fluids	ECF
Free fatty acid	ffa
Glycerol	glc
Glucose	glu
Glycogen	gly
Inflammation	inf
Intestine	INT
Ketone Bodies	keto
Lipoprotein Lipase	LPA
Liver	LVR
Muscle	MUS
Pancreas	PAN
Protein	pro
Reactive oxygen species	ROS
Triglycerides	tg
Adipocytes	ap
Kidney	KDY
Mitochondria	mito
Sarcopenia	sarc
Ketoacids	ketoa
Insulin Resistance	ISR
Lipolysis	lipo
Phosphatase	ppx
Lipid	lp
Eosinophil cationic protein	ecp

Dephosphorylation of insulin receptors	dep

S1.2 Table. Description of terms used in differential equations

Notation	Description
C_p^i	Concentration of species p in component i
$Cmax_p^i$	Maximum Concentration of species p in component i
m_p	Mass weight of species p
M_p	Mass of species p
MW_p	Molecular weight of species p
V^i	Volume of component i
$J_p^{i,j}$	Flux of species p from component i to j
$J_{p\_q}^{i,j}$	Flux of species p mediated by species q from component i to j
$J_{p\_q+r}^{i,j}$	Flux of species p mediated by species q and r from component i to j
$J_{p,q+r}^{i,j}$	Flux of species q and r from component i to j as a result of reaction of p at the cell surface
$h_p^{i,j}$	Flux rate of species p from component i to j
$R_{p,q}^i$	Reaction of species p to q in component i
R_{p,q_r}^i	Reaction of species p to q mediated by r in component i
$k_{p,q}^i$	Reaction rate of species p to q in component i
$kmax_{p,q}$	Maximum reaction rate of species p to q
$KM_{p,q}$	Half-maximal concentration of species p for reaction
$\beta_{p,q}$	Hill coefficient of reaction of species p to q
$\rho_{p,q}$	Stoichiometric ratio from species p to q
s_p^i	Source form of species p in component i
τ_p	Decomposition time scale of species p
Δ_p	Accumulation of species p as compared to the steady state
χ_p	Hydration coefficient of species p
