# Supplementary material for: A long-term mechanistic computational model of physiological factors driving the onset of type 2 diabetes in an individual
Source: PLoS One. 2018 Feb 14;13(2):e0192472. doi: 10.1371/journal.pone.0192472 (PMC5812629; doi:10.1371/journal.pone.0192472)
Supplement: S2 Table — (PDF) [file pone.0192472.s010.pdf]

S2 Table. Differential equations, expressions, parameters and variables of the blood compartment.

S2.1 Table. Differential equations by species in blood component.

Species	Ordinary Differential Equation
Glucose	$\frac{dC_{glu}^{BLD}}{dt} = \frac{J_{glu}^{INT,BLD} + J_{glu}^{LVR,BLD} - J_{glu}^{BLD,LVR} - J_{glu}^{BLD,MUS}}{V^{BLD} + V^{ECF}}$
Free Fatty Acids	$\frac{dC_{ffa}^{BLD}}{dt} = \frac{J_{ffa}^{ADI,BLD} + J_{ffa}^{MUS,BLD} - J_{ffa}^{BLD,LVR} - J_{ffa}^{BLD,MUS}}{V^{BLD} + V^{ECF}}$
Triglycerides	$\frac{dC_{tg}^{BLD}}{dt} = \frac{J_{tg}^{LVR,BLD} - J_{tg,ffa+glc}^{BLD,MUS} - J_{tg,ffa+glc}^{BLD,ADI}}{V^{BLD} + V^{ECF}}$
Glycerol	$\frac{dC_{glc}^{BLD}}{dt} = \frac{J_{glc}^{LVR,BLD} + J_{glc}^{MUS,BLD} + J_{glc}^{ADI,BLD} - J_{glc}^{BLD,LVR} - J_{glc}^{BLD,MUS} - J_{glc}^{BLD,ADI}}{V^{BLD} + V^{ECF}}$
Chylomicrons	$\frac{dC_{chy}^{BLD}}{dt} = \frac{J_{chy}^{INT,BLD} - J_{chy,ffa+glc}^{BLD,MUS} - J_{chy,ffa+glc}^{BLD,ADI}}{V^{BLD} + V^{ECF}}$
Ketone Bodies	$\frac{dC_{keto}^{BLD}}{dt} = \frac{J_{keto}^{LVR,BLD} - J_{keto}^{BLD,LVR} - J_{keto}^{BLD,KDY}}{V^{BLD} + V^{ECF}}$
Amino Acids	$\frac{dC_{aa}^{BLD}}{dt} = \frac{J_{aa}^{INT,BLD} + J_{aa}^{MUS,BLD} + J_{aa}^{LVR,BLD} - J_{aa}^{BLD,MUS} - J_{aa}^{BLD,LVR}}{V^{BLD}}$
HbA1c	$\frac{dC_{hba1c}^{BLD}}{dt} = R_{s+glu,hba1c}^{BLD} - R_{hba1c,s}^{BLD}$

S2.2 Table. Calculation of variables in differential equations in blood component.

S2.3	Equation	Ref. in Figure S1
$J_{glu}^{INT,BLD}$	CI	v_1^{BLD}
$J_{glu}^{BLD,LVR}$	$(h_{glu\_GLUT1}^{BLD,LVR} \times GLUT1 + h_{glu\_GLUT4}^{BLD,LVR} \times GT) \times C_{glu}^{BLD}$	v_2^{BLD}
$J_{glu}^{BLD,MUS}$	$(h_{glu\_GLUT1}^{BLD,MUS} \times GLUT1 + h_{glu\_GLUT4}^{BLD,MUS} \times GT) \times (C_{glu}^{BLD} - C_{glu}^{MUS})$	v_3^{BLD}
$J_{glu}^{LVR,BLD}$	$h_{glu}^{LVR,BLD} \times C_{glu}^{LVR}$	v_4^{BLD}
$J_{ffa}^{ADI,BLD}$	$h_{ffa}^{ADI,BLD} \times C_{ffa}^{ADI}$	v_5^{BLD}
$J_{ffa}^{BLD,LVR}$	$h_{ffa}^{BLD,LVR} \times C_{ffa}^{BLD}$	v_6^{BLD}
$J_{ffa}^{BLD,MUS}$	$(h_{ffa}^{BLD,MUS} + h_{ffa\_AMPK}^{BLD,MUS} + h_{ffa\_ins}^{BLD,MUS}) \times C_{ffa}^{BLD}$	v_7^{BLD}

S2.3	Equation	Ref. in Figure S1
$J_{tg}^{LVR,BLD}$	$h_{tg}^{LVR,BLD} \times C_{tg}^{LVR}$	v_{8}^{BLD}
$J_{tg,ffa+glc}^{BLD,MUS}$	$k_{tg,ffa+glc\_lpa}^{MUS} \times C_{tg}^{BLD} \times (1 + \alpha_{lipo\_LPA+AMPK})$	v_{9}^{BLD}
$J_{tg,ffa+glc}^{BLD,ADI}$	$k_{tg,ffa+glc\_LPA}^{ADI} \times C_{tg}^{BLD}$	v_{10}^{BLD}
$J_{glc}^{LVR,BLD}$	$h_{glc}^{LVR,BLD} \times C_{glc}^{LVR}$	v_{11}^{BLD}
$J_{glc}^{MUS,BLD}$	$h_{glc}^{MUS,BLD} \times C_{glc}^{MUS}$	v_{12}^{BLD}
$J_{glc}^{ADI,BLD}$	$h_{glc}^{ADI,BLD} \times C_{glc}^{ADI}$	v_{13}^{BLD}
$J_{glc}^{BLD,LVR}$	$h_{glc}^{BLD,LVR} \times C_{glc}^{BLD}$	v_{14}^{BLD}
$J_{glc}^{BLD,MUS}$	$h_{glc}^{BLD,MUS} \times C_{glc}^{BLD}$	v_{15}^{BLD}
$J_{glc}^{BLD,ADI}$	$h_{glc}^{BLD,ADI} \times C_{glc}^{BLD}$	v_{16}^{BLD}
$J_{chy}^{INT,BLD}$	FI	v_{17}^{BLD}
$J_{chy,ffa+glc}^{BLD,MUS}$	$k_{chy,ffa+glc\_lpa}^{MUS} \times C_{chy}^{BLD} \times (1 + \alpha_{lipo\_LPA+AMPK})$	v_{18}^{BLD}
$J_{chy,ffa+glc}^{BLD,ADI}$	$k_{chy,ffa+glc\_LPA}^{ADI} \times C_{chy}^{BLD}$	v_{19}^{BLD}
$J_{keto}^{LVR,BLD}$	$h_{keto}^{LVR,BLD} \times C_{keto}^{LVR}$	v_{20}^{BLD}
$J_{keto}^{BLD,LVR}$	$h_{keto}^{BLD,LVR} \times C_{keto}^{BLD}$	v_{21}^{BLD}
$J_{keto}^{BLD,KDY}$	$\alpha_{gfr} \times GFR \times C_{keto}^{BLD}$	v_{22}^{BLD}
$J_{aa}^{INT,BLD}$	$\rho_{pro,aa} \times PI$	v_{23}^{BLD}
$J_{aa}^{MUS,BLD}$	$h_{aa}^{MUS,BLD} \times C_{aa}^{MUS}$	v_{24}^{BLD}
$J_{aa}^{BLD,MUS}$	$h_{aa}^{BLD,MUS} \times C_{aa}^{BLD}$	v_{25}^{BLD}
$J_{aa}^{LVR,BLD}$	$h_{aa}^{LVR,BLD} \times C_{aa}^{LVR}$	v_{26}^{BLD}
$J_{aa}^{BLD,LVR}$	$h_{aa}^{BLD,LVR} \times C_{aa}^{BLD}$	v_{27}^{BLD}
$R_{s+glu,hba1c}^{BLD}$	$k_{s+glu,hba1c}^{BLD} \times C_{glu}^{BLD} \times \left(Cmax_{hba1c}^{BLD} - C_{hba1c}^{BLD}\right)$	v_{28}^{BLD}

S2.3	Equation	Ref. in Figure S1
$R_{hba1c,s}^{BLD}$	$C_{hba1c}^{BLD}/\tau_{hba1c}$	v_{29}^{BLD}
$J_{ffa}^{MUS,BLD}$	$\begin{cases} h_{ffa}^{MUS,BLD}\times\left(C_{ffa}^{MUS}-C_{ffa}^{ICF}\right), & if\ C_{ffa}^{MUS}>C_{ffa}^{ICF} \\ 0, & otherwise \end{cases}$	v_{30}^{BLD}

S2.3 Table. Additional variable calculations in blood component.

Variable	Equation
$\alpha_{lipo\_LPA+AMPK}$	$\begin{cases} \alpha_{lipo\_LPA+AMPK}\times\left(C_{AMP}^{MUS}-C_{ADP}^{MUS}\right), & if\ AMPK_{active}>AMPK_{active0}\ and\ PA>0 \\ 0, & otherwise \end{cases}$
$h_{ffa\_AMPK}^{BLD,MUS}$	$\begin{cases} h0_{ffa\_AMPK}^{BLD,MUS}\times\left(C_{AMP}^{MUS}-C_{ADP}^{MUS}\right), & if\ AMPK_{active}>AMPK_{active0}\ and\ PA>0 \\ 0, & otherwise \end{cases}$
$h_{ffa\_ins}^{BLD,MUS}$	$\begin{cases} h0_{ffa\_ins}^{BLD,MUS}\times(IS-IS\_SS), & if\ IS>IS\_SS \\ 0, & otherwise \end{cases}$
$h_{chy\_LPA+AMPK}^{BLD,MUS}$	$\begin{cases} h0_{chy\_LPA+AMPK}^{BLD,MUS}\times\left(C_{AMP}^{MUS}-C_{ADP}^{MUS}\right), & if\ AMPK_{active}>AMPK_{active0}\ and\ PA>0 \\ 0, & otherwise \end{cases}$
$AMPK_{active}$	$\dfrac{kmax_{AMP,AMPK}\times(C_{AMP}^{MUS})^2}{\left(KM_{AMP,AMPK}\right)^2+(C_{AMP}^{MUS})^2}$
$AMPK_{active0}$	$\dfrac{kmax_{AMP,AMPK}\times(C_{ADP}^{MUS})^2}{\left(KM_{AMP,AMPK}\right)^2+(C_{ADP}^{MUS})^2}$
GT	$\dfrac{\gamma_1}{1+\gamma_2*e^{-\gamma_3*(GLUT4-\gamma_4)}}$

S2.4 Table. Additional variable descriptions in blood component.

Variable	Description
$GLUT1$, $GLUT4$	Fractional amount representing GLUT1/GLUT4 concentration on the cell surface
CI	Carbohydrate (mMol/min)
FI	Fat intake (mMol/min)

Variable	Description
PI	Protein intake (mMol/min)
IS	Insulin sensitivity
PA	Excess energy expenditure per day from physical activity compared to that at the beginning of the study
GFR	Glomerular filtration rate
$\alpha_{lipo\_LPA+AMPK}$	Scaling factor of lipolysis mediated by LPA and AMPK
α_{gfr}	Scaling factor of glomerular filtration of keto bodies from blood to kidney
$\gamma_1, \gamma_2, \gamma_3, \gamma_4$	Constants used to represent the glucose transport mediated by GLUT4

S2.5 Table. Parameters related to the blood module.

Name	Value	Unit	Estimation Method
$C_{hba1c}^{BLD}(t=0)$	10 – 20	Percentage of Hb	Individual calibration
$Cmax_{hba1c}^{BLD}$	4 – 8	Percentage of Hb	
$h_{ffa\_AMPK}^{BLD,MUS}$	1.87×10^{-2}	$L\times min^{-1}$	Collectively estimated in baseline model
C_{ffa}^{ICF}	9.00×10^{-1}	μM	
$h_{aa}^{LVR,BLD}$	1.46×10^{0}	$L\times min^{-1}$	
$h_{aa}^{MUS,BLD}$	6.33×10^{0}	$L\times min^{-1}$	
$h_{ffa}^{MUS,BLD}$	5.00×10^{-3}	$L\times min^{-1}$	
$h_{glu\_GLUT1}^{BLD,X}$	3.03×10^{-1}	min^{-1}	
$h_{keto}^{BLD,LVR}$	5.00×10^{-1}	$L\times min^{-1}$	
$h_{keto}^{LVR,BLD}$	5.00×10^{-1}	$L\times min^{-1}$	

$k_{ros\_glu}^{BLD}$	5.78×10^{0}	min^{-1}	
γ_1	1.00×10^{0}	Dimensionless	
γ_2	1.00×10^{0}	Dimensionless	
γ_3	1.37×10^{2}	Dimensionless	
γ_4	2.50×10^{-1}	Dimensionless	
$\rho_{pro,aa}$	5.00×10^{0}	Dimensionless	
$\alpha_{lipo\_LPA+AMPK}$	6.65×10^{-2}	Dimensionless	
$kmax_{AMP,AMPK}$	3.00×10^{-7}	Dimensionless	
$KM_{AMP,AMPK}$	2.77×10^{-1}	Dimensionless	
α_{gfr}	1.00×10^{0}	Dimensionless	
τ_{hba1c}	3.82×10^{-6}	min^{-1}	
τ_{ins}	5.93×10^{-2}	min^{-1}	
τ_{Lp}	2.31×10^{-2}	min^{-1}	
