# Supplementary material for: A long-term mechanistic computational model of physiological factors driving the onset of type 2 diabetes in an individual
Source: PLoS One. 2018 Feb 14;13(2):e0192472. doi: 10.1371/journal.pone.0192472 (PMC5812629; doi:10.1371/journal.pone.0192472)
Supplement: S3 Table — (PDF) [file pone.0192472.s011.pdf]

S3 Table. Differential equations, expressions and variables of the muscle compartment.

S3.1 Table. Differential equations by species in muscle component.

Species	Ordinary Differential Equation
Glucose	$\frac{dC_{glu}^{MUS}}{dt} = \frac{J_{glu}^{BLD,MUS}}{V^{MUS}} + \rho_{gly,glu} \times R_{gly,glu}^{MUS} - R_{glu,gly}^{MUS} - R_{glu,ffa}^{MUS} - R_{glu+ADP,ATP}^{MUS}$
Glycogen	$\frac{dC_{gly}^{MUS}}{dt} = \frac{1}{\rho_{gly,glu}} \times R_{glu,gly}^{MUS} - R_{gly,glu}^{MUS}$
Free Fatty Acids	$\frac{dC_{ffa}^{MUS}}{dt} = \frac{\rho_{chy,ffa} \times J_{chy,ffa+glc}^{BLD,MUS} + \rho_{tg,ffa} \times J_{tg,ffa+glc}^{BLD,MUS} + J_{ffa}^{BLD,MUS} - J_{ffa}^{MUS,BLD}}{V^{MUS}} + \rho_{tg,ffa} \times R_{tg,ffa+glc}^{MUS} + \rho_{glu,ffa} \times R_{glu,ffa}^{MUS} - R_{ffa+ADP,ATP}^{MUS} - R_{ffa+glc,tg}^{MUS}$
Triglycerides	$\frac{dC_{tg}^{MUS}}{dt} = R_{ffa+glc,tg}^{MUS} - R_{tg,ffa+glc}^{MUS}$
Glycerol	$\frac{dC_{glc}^{MUS}}{dt} = \frac{J_{glc}^{BLD,MUS} + J_{chy,ffa+glc}^{BLD,MUS} + J_{tg,ffa+glc}^{BLD,MUS} - J_{glc}^{MUS,BLD}}{V^{MUS}} + R_{tg,ffa+glc}^{MUS} - R_{ffa+glc,tg}^{MUS}$
Amino Acids	$\frac{dC_{aa}^{MUS}}{dt} = \frac{J_{aa}^{BLD,MUS} - J_{aa}^{MUS,BLD}}{V^{MUS} + V^{ECF}} + \rho_{aa,pro} \times R_{pro,aa}^{MUS} - R_{aa,pro}^{MUS} - R_{aa,ketoa}^{MUS}$
Protein	$\frac{dC_{pro}^{MUS}}{dt} = \frac{1}{\rho_{aa,pro}} \times R_{aa,pro}^{MUS} - R_{pro,aa}^{MUS}$
Ketoacids	$\frac{dC_{ketoa}^{MUS}}{dt} = R_{aa,ketoa}^{MUS} - R_{ketoa+ADP,ATP}^{MUS}$
ATP	$\frac{dC_{ATP}^{BLD}}{dt} = \rho_{glu,ATP} \times R_{glu+ADP,ATP}^{MUS} + \rho_{ffa,ATP} \times R_{ffa+ADP,ATP}^{MUS} + \rho_{ketoa,ATP} \times R_{ketoa+ADP,ATP}^{MUS} - R_{ATP,ADP}^{MUS}$

S3.2 Table. Calculation of variables in differential equations in muscle component.

Variable	Equation	Ref. in Figure S2
$J_{glu}^{BLD,MUS}$	$\left(h_{glu\_GLUT1}^{BLD,MUS} \times GLUT1 + h_{glu\_GLUT4}^{BLD,MUS} \times GT\right) \times \left(C_{glu}^{BLD} - C_{glu}^{MUS}\right)$	v_1^{MUS}
$J_{ffa}^{BLD,MUS}$	$\left(h_{ffa}^{BLD,MUS} + h_{ffa\_AMPK}^{BLD,MUS} + h_{ffa\_ins}^{BLD,MUS}\right) \times C_{ffa}^{BLD}$	v_2^{MUS}
$J_{ffa}^{MUS,BLD}$	$\begin{cases} h_{ffa}^{MUS,BLD} \times \left(C_{ffa}^{MUS} - C_{ffa}^{ICF}\right), & if\ C_{ffa}^{MUS} > C_{ffa}^{ICF} \\ 0, & otherwise \end{cases}$	v_3^{MUS}

Variable	Equation	Ref. in Figure S2
$J_{glc}^{MUS,BLD}$	$h_{glc}^{MUS,BLD} \times C_{glc}^{MUS}$	v_4^{MUS}
$J_{aa}^{BLD,MUS}$	$h_{aa}^{BLD,MUS} \times C_{aa}^{BLD}$	v_5^{MUS}
$J_{aa}^{MUS,BLD}$	$h_{aa}^{MUS,BLD} \times C_{aa}^{MUS}$	v_6^{MUS}
$R_{glu,gly}^{MUS}$	$k_{glu,gly}^{MUS} \times C_{glu}^{MUS} \times \left(Cmax_{gly}^{MUS} - C_{gly}^{MUS}\right) \times IS$	v_7^{MUS}
$R_{glu,ffa}^{MUS}$	$\alpha_{glu,ffa}^{MUS} \times \frac{\left(C_{glu}^{MUS}\right)^{\beta_{glu,ffa}}}{\left(KM_{glu,ffa}\right)^{\beta_{glu,ffa}} + \left(C_{glu}^{MUS}\right)^{\beta_{glu,ffa}}}$	v_8^{MUS}
$R_{glu+ADP,ATP}^{MUS}$	$\alpha_{glu+ADP,ATP} \times \frac{C_{glu}^{MUS}}{KM_{glu+ADP,ATP}+C_{glu}^{MUS}} \times C_{ADP}^{MUS} \times C_{mito}^{MUS}$	v_9^{MUS}
$R_{gly,glu}^{MUS}$	$k_{gly,glu}^{MUS} \times C_{gly}^{MUS} \times \frac{1}{1 + \left(\frac{AAR}{AAR_{SS} \times KI_{ATP,s}}\right)^{\beta_{ATP,s}}}$	v_{10}^{MUS}
$J_{chy,ffa+glc}^{BLD,MUS}$	$k_{chy,ffa+glc\_lpa}^{MUS} \times C_{chy}^{BLD} \times (1 + \alpha_{lipo\_LPA+AMPK})$	v_{11}^{MUS}
$J_{tg,ffa+glc}^{BLD,MUS}$	$k_{tg,ffa+glc\_lpa}^{MUS} \times C_{tg}^{BLD} \times (1 + \alpha_{lipo\_LPA+AMPK})$	v_{12}^{MUS}
$R_{ffa+ADP,ATP}^{MUS}$	$\alpha_{ffa+ADP,ATP} \times \frac{C_{ffa}^{MUS}}{KM_{ffa+ADP,ATP} + C_{ffa}^{MUS}} \times C_{ADP}^{MUS} \times C_{mito}^{MUS}$	v_{13}^{MUS}
$R_{ffa+glc,tg}^{MUS}$	$k_{ffa,tg}^{MUS} \times C_{ffa}^{MUS} \times C_{glc}^{MUS}$	v_{14}^{MUS}
$R_{tg,ffa+glc}^{MUS}$	$k_{tg,ffa+glc}^{MUS} \times C_{tg}^{MUS} \times \frac{\alpha_{lipo\_PA}}{1 + \left(\frac{IS}{KI_{lipo\_ins}}\right)^{\beta_{tg,ffa+glc\_ins}}}$	v_{15}^{MUS}
$R_{aa,pro}^{MUS}$	$k_{aa,pro}^{MUS} \times C_{aa}^{MUS}$	v_{16}^{MUS}
$R_{pro,aa}^{MUS}$	$k_{pro,aa}^{MUS} \times C_{pro}^{MUS} \times \alpha_{pro,aa\_AGE}$	v_{17}^{MUS}
$R_{aa,ketoa}^{MUS}$	$k_{aa,ketoa}^{MUS} \times C_{aa}^{MUS} \times (1 + \alpha_{aa,ketoa\_PI}^{MUS} \times \frac{PI}{PI_0})$	v_{18}^{MUS}
$R_{ketoa+ADP,ATP}^{MUS}$	$\alpha_{ketoa+ADP,ATP} \times C_{ketoa}^{MUS} \times C_{ADP}^{MUS} \times C_{mito}^{MUS}$	v_{19}^{MUS}
$R_{ATP,ADP}^{MUS}$	$k_{ATP,ADP}^{MUS} \times C_{ATP}^{MUS} \times (1 + \alpha_{RMR\_FFM} \times (M_{FFM} - M_{FFM0}) + \alpha_{RMR\_FM} \times (M_{FM} - M_{FM0})) \times \Delta RMR \times RMR_{adaptation}$	v_{20}^{MUS}
$J_{glc}^{BLD,MUS}$	$h_{glc}^{BLD,MUS} \times C_{glc}^{BLD}$	v_{21}^{MUS}

S3.3 Table. Additional variable calculations in muscle component.

Variable	Equation
AAR	$\frac{C_{ATP}^{MUS}}{C_{ADP}^{MUS}}$
M_{FM}	$\left(C_{tg}^{LVR}\times V^{LVR}+C_{tg}^{MUS}\times V^{MUS}+C_{tg}^{ADI}\times V^{ADI}\right)\times MG_{tg}$
M_{gly}	$\left(C_{gly}^{LVR}\times V^{LVR}+C_{gly}^{MUS}\times V^{MUS}\right)\times MG_{gly}$
M_{pro}	$\left(C_{pro}^{LVR}\times V^{LVR}+C_{pro}^{MUS}\times V^{MUS}\right)\times MG_{pro}$
M_{FFM}	$\frac{\chi_{FM}\times M_{FM}+M_{gly}+M_{pro}+M_{bone}+M_{ecp}}{1-\chi_{FFM}}$
$h_{ffa\_AMPK}^{BLD,MUS}$	$\begin{cases} h_{ffa\_AMPK}^{BLD,MUS}\times\left(C_{AMP}^{MUS}-C_{ADP}^{MUS}\right), & if\ AMPK_{active}>AMPK_{active0}\ and\ PA>0 \\ 0, & otherwise \end{cases}$
$h_{ffa\_ins}^{BLD,MUS}$	$\begin{cases} h_{ffa\_ins}^{BLD,MUS}\times(IS-IS\_SS), & if\ IS>IS\_SS \\ 0, & otherwise \end{cases}$
$\alpha_{lipo\_LPA+AMPK}$	$\begin{cases} \alpha_{lipo\_LPA+AMPK}\times\left(C_{AMP}^{MUS}-C_{ADP}^{MUS}\right), & if\ AMPK_{active}>AMPK_{active0}\ and\ PA>0 \\ 0, & otherwise \end{cases}$
α_{lipo_PA}	$1+\alpha_{lipo\_PA0}\times\frac{(percent\_vO2max)^{\beta_{tg,ffa+glc\_PA}}}{(percent\_vO2max)^{\beta_{tg,ffa+glc\_PA}}+\left(KM_{tg,ffa+glc\_PA}\right)^{\beta_{tg,ffa+glc\_PA}}}$
ΔRMR	$1+\gamma_5\times(\Delta PA-1)$
$RMR_{adaptation}$	$\alpha_{RMR\_adaptation}\times\frac{\left(C_{lp}^{BLD}\right)^{\beta_{RMR\_adaptation}}}{\left(KM_{RMR\_adaptation}\right)^{\beta_{RMR\_adaptation}}+\left(C_{lp}^{BLD}\right)^{\beta_{RMR\_adaptation}}}$
$AMPK_{active}$	$\frac{kmax_{AMP,AMPK}\times(C_{AMP}^{MUS})^2}{\left(KM_{AMP,AMPK}\right)^2+(C_{AMP}^{MUS})^2}$
$AMPK_{active0}$	$\frac{kmax_{AMP,AMPK}\times(C_{ADP}^{MUS})^2}{\left(KM_{AMP,AMPK}\right)^2+(C_{ADP}^{MUS})^2}$

S3.4 Table. Additional variable definitions in muscle component.

Variable	Description
IS	Insulin sensitivity
AAR	Ratio of concentration of ATP to ADP
AAR_{SS}	Ratio of concentration of ATP to ADP at steady state
MTW_0	Baseline muscle to weight ratio
M_{FFM}	Fat free mass
M_{FFM0}	Baseline fat free mass
M_{FM}	Fat mass
M_{FM0}	Baseline fat mass
$KI_{ATP,s}$	ATP depletion regulated glycogenolysis inhibition scaling factor
KI_{lipo_ins}	Insulin sensitivity mediated lipolysis inhibition scaling factor
ΔRMR	Physical activity dependent relative change of resting metabolic rate
$RMR_{adaptation}$	Adaptation of RMR to weight change
ΔPA	Relative physical activity
$percent\_vO2max$	Maximal oxygen consumption percentage
$\alpha_{glu,ffa}^{MUS}$	Scaling factor of reaction from glucose to free fatty acids in muscle
$\alpha_{glu+ADP,ATP}$	Scaling factor of aerobic reaction from glucose and ADP to ATP
$\alpha_{glu+ADP,ATP_{ana}}$	Scaling factor of anaerobic reaction from glucose and ADP to ATP
$\alpha_{lipo\_LPA+AMPK}$	Scaling factor of lipolysis mediated by LPA and AMPK

Variable	Description
$\alpha_{ffa+ADP,ATP}$	Scaling factor of reaction from free fatty acids and ADP to ADP
α_{lipo_PA}	Scaling factor of physical activity mediated lipolysis of triglycerides
α_{lipo_PA0}	Baseline scaling factor of physical activity mediated lipolysis of triglycerides
$\alpha_{aa,ketoa\_PI}^{MUS}$	Scaling factor of protein intake dependent reaction from amino acids to ketoacids in muscle
$\alpha_{ketoa+ADP,ATP}$	Scaling factor of reaction from ketoacids to ADP
α_{RMR_FFM}	Scaling factor of free fat mass dependent resting metabolism rate change
α_{RMR_FM}	Scaling factor of fat mass dependent resting metabolism rate change
$\alpha_{RMR\_adaptation}$	Scaling factor of weight dependent RMR reduction
γ_5	Percentage impact of physical activity on resting metabolic rate

S3.5 Table. Parameters related to the muscle module.

Name	Value	Unit	Estimation Method
$h_{ffa\_AMPK}^{BLD,MUS}$	1.87×10^{-2}	$L\times min^{-1}$	Collectively estimated in baseline model
$h_{aa}^{MUS,BLD}$	6.33×10^{0}	$L\times min^{-1}$	
$h_{glu\_GLUT1}^{BLD,X}$	3.03×10^{-1}	min^{-1}	
$k_{pro,aa}^{MUS}$	1.52×10^{0}	min^{-1}	
$k_{glu,gly}$	3.74×10^{-4}	$min^{-1}\times mM^{-1}$	
$k_{gly,glu}$	6.32×10^{-4}	min^{-1}	

$k_{glu,ffa}$	3.71×10^{-1}	Dimensionless	
$KM_{glu+ADP,ATP}$	8.01×10^{-1}	mM	
$KM_{ffa+ADP,ATP}$	7.22×10^{-1}	mM	
$\alpha_{lipo\_LPA+AMPK}$	6.65×10^{-2}	Dimensionless	
$\alpha_{aa,ketoa\_PI}^{MUS}$	1.00×10^{1}	Dimensionless	
χ_{FM}	1.50×10^{-1}	Dimensionless	
χ_{FFM}	7.54×10^{-1}	Dimensionless	
α_{RMR_FFM}	5.77×10^{-3}	Dimensionless	
α_{RMR_FM}	1.30×10^{-2}	Dimensionless	
γ_5	2.60×10^{-1}	Dimensionless	
AAR_{SS}	2.00×10^{1}	Dimensionless	
