# Supplementary material for: A long-term mechanistic computational model of physiological factors driving the onset of type 2 diabetes in an individual
Source: PLoS One. 2018 Feb 14;13(2):e0192472. doi: 10.1371/journal.pone.0192472 (PMC5812629; doi:10.1371/journal.pone.0192472)
Supplement: S4 Table — (PDF) [file pone.0192472.s012.pdf]

S4 Table. Differential equations, expressions and variables of the liver compartment.

S4.1 Table. Differential equations by species in liver component.

Species	Ordinary Differential Equation
Glucose	$\frac{dC_{glu}^{LVR}}{dt}=\frac{J_{glu}^{BLD,LVR}-J_{glu}^{LVR,BLD}}{V^{LVR}}+\rho_{gly,glu}\times R_{gly,glu}^{LVR}+R_{glc,glu}^{LVR}+R_{ketoa,glu}^{LVR}-R_{glu,ffa}^{LVR}-R_{glu,gly}^{LVR}$
Glycogen	$\frac{dC_{gly}^{LVR}}{dt}=\frac{1}{\rho_{gly,glu}}\times R_{glu,gly}^{LVR}-R_{gly,glu}^{LVR}$
Glycerol	$\frac{dC_{glc}^{LVR}}{dt}=\frac{J_{glc}^{BLD,LVR}-J_{glc}^{LVR,BLD}}{V^{LVR}}-R_{glc,glu}^{LVR}-R_{ffa+glc,tg}^{LVR}$
Free Fatty Acids	$\frac{dC_{ffa}^{LVR}}{dt}=\frac{J_{ffa}^{BLD,LVR}}{V^{LVR}}+\rho_{glu,ffa}\times R_{glu,ffa}^{LVR}+R_{keto,ffa}^{LVR}-R_{ffa,keto}^{LVR}-\rho_{tg,ffa}\times R_{ffa+glc,tg}^{LVR}$
Triglycerides	$\frac{dC_{tg}^{LVR}}{dt}=\frac{-J_{tg}^{LVR,BLD}}{V^{LVR}}+R_{ffa+glc,tg}^{LVR}$
Keto Bodies	$\frac{dC_{keto}^{LVR}}{dt}=\frac{J_{keto}^{BLD,LVR}-J_{keto}^{LVR,BLD}}{V^{LVR}}+R_{ffa,keto}^{LVR}-R_{keto,ffa}^{LVR}$
Amino Acids	$\frac{dC_{aa}^{LVR}}{dt}=\frac{J_{aa}^{BLD,LVR}-J_{aa}^{LVR,BLD}}{V^{LVR}}+\rho_{aa,pro}\times R_{pro,aa}^{LVR}-R_{aa,pro}^{LVR}-R_{aa,ketoa}^{LVR}$
Protein	$\frac{dC_{pro}^{LVR}}{dt}=\frac{1}{\rho_{aa,pro}}\times R_{aa,pro}^{LVR}-R_{pro,aa}^{LVR}$
Ketoacids	$\frac{dC_{ketoa}^{LVR}}{dt}=R_{aa,ketoa}^{LVR}-R_{ketoa,glu}^{LVR}$

S4.2 Table. Calculation of variables in differential equations in liver component.

Variable	Equation	Ref. in Figure S3
$J_{glu}^{BLD,LVR}$	$\left(h_{glu\_GLUT1}^{BLD,LVR}\times GLUT1+h_{glu\_GLUT4}^{BLD,LVR}\times GT\right)\times C_{glu}^{BLD}$	v_1^{LVR}
$J_{glu}^{LVR,BLD}$	$h_{glu}^{LVR,BLD}\times C_{glu}^{LVR}$	v_2^{LVR}
$J_{ffa}^{BLD,LVR}$	$h_{ffa}^{BLD,LVR}\times C_{ffa}^{BLD}$	v_3^{LVR}
$J_{tg}^{LVR,BLD}$	$h_{tg}^{LVR,BLD}\times C_{tg}^{LVR}$	v_4^{LVR}
$J_{glc}^{BLD,LVR}$	$h_{glc}^{BLD,LVR}\times C_{glc}^{BLD}$	v_5^{LVR}

Variable	Equation	Ref. in Figure S3
$J_{glc}^{LVR,BLD}$	$h_{glc}^{LVR,BLD} \times C_{glc}^{LVR}$	v_6^{LVR}
$J_{keto}^{BLD,LVR}$	$h_{keto}^{BLD,LVR} \times C_{keto}^{BLD}$	v_7^{LVR}
$J_{keto}^{LVR,BLD}$	$h_{keto}^{LVR,BLD} \times C_{keto}^{LVR}$	v_8^{LVR}
$J_{aa}^{BLD,LVR}$	$h_{aa}^{BLD,LVR} \times C_{aa}^{BLD}$	v_9^{LVR}
$J_{aa}^{LVR,BLD}$	$h_{aa}^{LVR,BLD} \times C_{aa}^{LVR}$	v_{10}^{LVR}
$R_{glu,gly}^{LVR}$	$k_{glu,gly}^{LVR} \times C_{glu}^{LVR} \times \left(Cmax_{gly}^{LVR} - C_{gly}^{LVR}\right) \times IS$	v_{11}^{LVR}
$R_{glu,ffa}^{LVR}$	$\alpha_{glu,ffa}^{LVR} \times \frac{\left(C_{glu}^{LVR}\right)^{\beta_{glu,ffa}}}{\left(KM_{glu,ffa}\right)^{\beta_{glu,ffa}} + \left(C_{glu}^{LVR}\right)^{\beta_{glu,ffa}}}$	v_{12}^{LVR}
$R_{gly,glu}^{LVR}$	$k_{gly,glu}^{LVR} \times C_{gly}^{LVR} \times \frac{1 + \alpha_{gly,glu\_PA}^{LVR}}{1 + \left(\frac{AAR}{AAR_{SS} \times KI_{ATP,s}}\right)^{\beta_{ATP,s}}}$	v_{13}^{LVR}
$R_{ffa+glc,tg}^{LVR}$	$k_{ffa,tg}^{LVR} \times C_{ffa}^{LVR} \times C_{glc}^{LVR}$	v_{14}^{LVR}
$R_{ffa,keto}^{LVR}$	$k_{ffa,keto}^{LVR} \times \frac{\left(C_{ffa}^{LVR}\right)^{\beta_{ffa,keto}}}{\left(KM_{ffa,keto}\right)^{\beta_{ffa,keto}} + \left(C_{ffa}^{LVR}\right)^{\beta_{ffa,keto}}}$	v_{15}^{LVR}
$R_{keto,ffa}^{LVR}$	$k_{keto,ffa}^{LVR} \times C_{keto}^{LVR}$	v_{16}^{LVR}
$R_{glc,glu}^{LVR}$	$k_{glc,glu}^{LVR} \times C_{glc}^{LVR} \times \frac{1}{1 + \frac{IS}{KI_{gngf\_ins}}}$	v_{17}^{LVR}
$R_{aa,pro}^{LVR}$	$k_{aa,pro}^{LVR} \times C_{aa}^{LVR}$	v_{18}^{LVR}
$R_{pro,aa}^{LVR}$	$k_{pro,aa}^{LVR} \times C_{pro}^{LVR}$	v_{19}^{LVR}
$R_{aa,ketoa}^{LVR}$	$k_{aa,ketoa}^{LVR} \times C_{aa}^{LVR} \times (1 + \alpha_{aa,ketoa\_PI}^{LVR} \times \frac{PI}{PI_0})$	v_{20}^{LVR}
$R_{ketoa,glu}^{LVR}$	$k_{ketoa,glu}^{LVR} \times C_{ketoa}^{LVR}$	v_{21}^{LVR}

S4.3 Table. Additional variable definitions in liver component.

Variable	Description
$\alpha_{glu,ffa}^{LVR}$	Scaling factor of reaction from glucose to free fatty acids in liver
$\alpha_{gly,glu\_PA}^{LVR}$	Scaling factor of physical activity dependent glycogenolysis in liver
$\alpha_{aa,ketoa\_PI}^{LVR}$	Scaling factor of protein intake dependent reaction from amino acids to ketoacids in liver
AAR	Ratio of concentration of ATP to ADP
AAR_{SS}	Ratio of concentration of ATP to ADP at steady state
$KI_{ATP,s}$	ATP depletion regulated glycogenolysis inhibition scaling factor
KI_{gngf_ins}	Insulin mediated gluconeogenesis from fructose inhabitation scaling factor

S4.4 Table. Parameters related to the liver module.

Name	Value	Unit	Estimation Method
$h_{aa}^{LVR,BLD}$	1.46×10^{0}	$L\times min^{-1}$	Collectively estimated in baseline model
$h_{keto}^{BLD,LVR}$, $h_{keto}^{LVR,BLD}$	5.00×10^{-1}	$L\times min^{-1}$	
$k_{ketoa,glu}^{LVR}$	2.88×10^{-2}	min^{-1}	
$k_{pro,aa}^{LVR}$	9.92×10^{-1}	min^{-1}	
$k_{glu,gly}$	3.74×10^{-4}	$min^{-1}\times mM^{-1}$	
$k_{gly,glu}$	6.32×10^{-4}	min^{-1}	
AAR_{SS}	2.00×10^{1}	Dimensionless	
$KI_{ATP,s}$	4.00×10^{1}	min^{-1}	
$\beta_{ffa,keto}$	6.00×10^{0}	Dimensionless	
$KM_{ffa,keto}$	1.25×10^{0}	mM	
KI_{gngf_ins}	1.47×10^{2}	min^{-1}	
