# Supplementary material for: A long-term mechanistic computational model of physiological factors driving the onset of type 2 diabetes in an individual
Source: PLoS One. 2018 Feb 14;13(2):e0192472. doi: 10.1371/journal.pone.0192472 (PMC5812629; doi:10.1371/journal.pone.0192472)
Supplement: S5 Table — (PDF) [file pone.0192472.s013.pdf]

S5 Table. Differential equations, expressions and variables of the adipose compartment.

S5.1 Table. Differential equations by species in adipose component.

Species	Ordinary Differential Equation
Free Fatty Acids	$\frac{dC_{ffa}^{ADI}}{dt} = \frac{\rho_{chy,ffa} \times J_{chy,ffa+glc}^{BLD,ADI} + \rho_{tg,ffa} \times J_{tg,ffa+glc}^{BLD,ADI} - J_{ffa}^{ADI,BLD}}{V^{ADI}} + \rho_{tg,ffa} \times R_{tg,ffa+glc}^{ADI} - \rho_{tg,ffa} \times R_{ffa+glc,tg}^{ADI}$
Triglycerides	$\frac{dC_{tg}^{ADI}}{dt} = R_{ffa+glc,tg}^{ADI} - R_{tg,ffa+glc}^{ADI}$
Glycerol	$\frac{dC_{glc}^{ADI}}{dt} = \frac{J_{glc}^{ADI} + J_{glc}^{BLD,ADI} + J_{chy,ffa+glc}^{BLD,ADI} + J_{tg,ffa+glc}^{BLD,ADI} - J_{glc}^{ADI,BLD}}{V^{ADI}} + R_{tg,ffa+glc}^{ADI} - R_{ffa+glc,tg}^{ADI}$
N_{ap}	$\frac{dN_{ap}}{dt} = R_{s,ap}^{ADI} + R_{s,ap\_tg}^{ADI} - R_{ap,s}^{ADI}$

S5.2 Table. Calculation of variables in differential equations in adipose component.

Variable	Equation	Ref. in Figure S4
$J_{ffa}^{ADI,BLD}$	$h_{ffa}^{ADI,BLD} \times C_{ffa}^{ADI}$	v_1^{ADI}
$J_{chy,ffa+glc}^{BLD,ADI}$	$k_{chy,ffa+glc\_LPA}^{ADI} \times C_{chy}^{BLD}$	v_2^{ADI}
$J_{tg,ffa+glc}^{BLD,ADI}$	$k_{tg,ffa+glc\_LPA}^{ADI} \times C_{tg}^{BLD}$	v_3^{ADI}
$R_{tg,ffa+glc}^{ADI}$	$k_{tg,ffa+glc}^{ADI} \times C_{tg}^{ADI} \times \frac{\alpha_{lipo\_PA}}{1 + \left(\frac{IS}{KI_{lipo\_ins}}\right)^{\beta_{tg,ffa+glc\_ins}}}$	v_4^{ADI}
$R_{ffa+glc,tg}^{ADI}$	$k_{ffa+glc,tg}^{ADI} \times C_{ffa}^{ADI} \times C_{glc}^{ADI}$	v_5^{ADI}
J_{glc}^{ADI}	$k_{s,glc}$	v_6^{ADI}
$J_{glc}^{BLD,ADI}$	$h_{glc}^{ADI,BLD} \times C_{glc}^{BLD}$	v_7^{ADI}
$R_{s,ap}^{ADI}$	$k_{s,ap}$	v_8^{ADI}
R_{s,ap_tg}^{ADI}	$\begin{cases} \alpha_{s,ap\_tg} \times k_{s,ap} \times \frac{(\Delta lp)^{\beta_{s,ap\_tg}}}{\left(KM_{s,ap\_tg}\right)^{\beta_{s,ap\_tg}} + (\Delta lp)^{\beta_{s,ap\_tg}}}, & if\ \Delta lp \geq 0 \\ 0, & otherwise \end{cases}$	v_9^{ADI}

Variable	Equation	Ref. in Figure S4
$R_{ap,s}^{ADI}$	$\tau_{ap} \times N_{ap}$	v_{10}^{ADI}
$J_{glc}^{ADI,BLD}$	$h_{glc}^{ADI,BLD} \times C_{glc}^{ADI}$	v_{11}^{ADI}

S5.3 Table. Additional variable calculations in adipose component.

Variable	Equation
α_{lipo_PA}	$1 + \alpha_{lipo\_PA0} \times \frac{(percent\_vO2max)^{\beta_{tg,ffa+glc\_PA}}}{(percent\_vO2max)^{\beta_{tg,ffa+glc\_PA}} + \left(KM_{tg,ffa+glc\_PA}\right)^{\beta_{tg,ffa+glc\_PA}}}$
Δlp	$\frac{M_{FM}}{N_{ap}} - M_{tg0}$
M_{FM}	$\left(C_{tg}^{LVR} \times V^{LVR} + C_{tg}^{MUS} \times V^{MUS} + C_{tg}^{ADI} \times V^{ADI}\right) \times MG_{tg}$

S5.4 Table. Additional variable descriptions in adipose component.

Variable	Description
N_{ap}	Number of adipocytes
KI_{lipo_ins}	Insulin sensitivity mediated lipolysis inhibition scaling factor
M_{FM}	Body fat mass
M_{tg0}	Baseline mass of triglycerides
α_{lipo_PA}	Scaling factor of physical activity mediated lipolysis of triglycerides
α_{lipo_PA0}	Baseline scaling factor of physical activity mediated lipolysis of triglycerides
α_{s,ap_tg}	Scaling factor of lipid content dependent on adipogenesis
$percent\_vO2max$	Maximal oxygen consumption percentage

S5.5 Table. Parameters related to the adipose module.

Name	Value	Unit	Estimation Method
$h_{glu\_GLUT1}^{BLD,X}$	3.03×10^{-1}	min^{-1}	Collectively estimated in baseline model
$k_{ffa+glc,tg}$	1.17×10^{-2}	min^{-1}	
KI_{lipo_ins}	5.56×10^{-1}	min^{-1}	
$\beta_{tg,ffa+glc\_ins}$	2.00×10^{0}	Dimensionless	
α_{s,ap_tg}	2.50×10^{1}	Dimensionless	
