# Supplementary material for: A long-term mechanistic computational model of physiological factors driving the onset of type 2 diabetes in an individual
Source: PLoS One. 2018 Feb 14;13(2):e0192472. doi: 10.1371/journal.pone.0192472 (PMC5812629; doi:10.1371/journal.pone.0192472)
Supplement: S6 Table — (PDF) [file pone.0192472.s014.pdf]

S6 Table. Differential equations, expressions and variables of the pancreas compartment.

S6.1 Table. Differential equations by species in pancreas component.

Species	Ordinary Differential Equation
Beta Cell (Mass)	$\frac{dN_{bc}}{dt} = R_{s,bc}^{PAN} - R_{bc,s}^{PAN}$
Beta Cell (Capacity Damage)	$\frac{dbcd}{dt} = R_{s,bcd}^{PAN} - R_{bcd,s}^{PAN}$
Insulin	$\frac{dC_{ins}^{BLD}}{dt} = R_{s,ins}^{BLD} - R_{ins,s}^{BLD}$

S6.2 Table. Calculation of variables in differential equations in pancreas component.

Variable	Equation	Ref. in Figure S5
$R_{s,bc}^{PAN}$	$\alpha_{s,bc} \times \frac{\left(C_{glu}^{BLD}\right)^{\beta_{s,bc}}}{\left(KM_{s,bc}\right)^{\beta_{s,bc}} + \left(C_{glu}^{BLD}\right)^{\beta_{s,bc}}}$	v_1^{PAN}
$R_{bc,s}^{PAN}$	$\alpha_{bc,s} \times \left(1 + \alpha_{bc,s\_ros} \times \Delta C_{ros}^{BLD}\right) \times N_{bc}$	v_2^{PAN}
$R_{s,bcd}^{PAN}$	$\alpha_{s,bcd\_ros} \times \Delta C_{ros}^{BLD} \times (1 - bcd)$	v_3^{PAN}
$R_{bcd,s}^{PAN}$	bcd/τ_{bcd}	v_4^{PAN}
$R_{s,ins}^{BLD}$	$\left(k_{s,ins\_bc}^{BLD} + k_{s,ins\_glu}^{BLD} \times (1 - bcd) \times R_{s,ins\_glu}^{BLD} \times R_{s,ins\_ffa}^{BLD}\right) \times N_{bc}$	v_5^{PAN}
$R_{ins,s}^{BLD}$	C_{ins}^{BLD}/τ_{ins}	v_6^{PAN}

S6.3 Table. Additional variable calculations in pancreas component.

Variable	Equation
ΔC_{ros}^{BLD}	$k_{ros\_glu}^{BLD} \times \Delta C_{glu}^{BLD} + \alpha_{ros\_inf} \times R_{ros\_inf}^{PAN} + \alpha_{ros\_ffa} \times \frac{\Delta C_{ffa}^{BLD}}{2 \times KM_{ros\_ffa} + \Delta C_{ffa}^{BLD}}$
ΔC_{glu}^{BLD}	$\max\left(C_{glu}^{BLD} - C_{glu\_SS}^{BLD}, \quad 0\right)$
ΔC_{ffa}^{BLD}	$\max\left(C_{ffa}^{BLD} - C_{ffa\_SS}^{BLD}, \quad 0\right)$

Variable	Equation
$R_{ros\_inf}^{PAN}$	$\frac{\left(C_{inf}^{BLD}\right)^{\beta_{ros\_inf}}}{\left(KM_{ros\_inf}\right)^{\beta_{ros\_inf}} + \left(C_{inf}^{BLD}\right)^{\beta_{ros\_inf}}}$
R_{s,ins_glu}^{BLD}	$\frac{\left(C_{glu}^{BLD}\right)^{\beta_{s.ins\_glu}}}{\left(KM_{s,ins\_glu}\right)^{\beta_{s.ins\_glu}} + \left(C_{glu}^{BLD}\right)^{\beta_{s.ins\_glu}}}$
R_{s,ins_ffa}^{BLD}	$1 + \alpha_{s,ins\_ffa} \times \frac{\left(C_{ffa}^{BLD}\right)^{\beta_{s,ins\_ffa}}}{\left(KM_{s,ins\_ffa}\right)^{\beta_{s,ins\_ffa}} + \left(C_{ffa}^{BLD}\right)^{\beta_{s,ins\_ffa}}}$

S6.4 Table. Additional variable descriptions in pancreas component.

Variable	Description
N_{bc}	Number of beta cells
bcd	Damage to beta cells, range between 0 and 1
$\alpha_{s,bc}$	Scaling factor of beta cell growth
$\alpha_{bc,s}$	Scaling factor of beta cell apoptosis
α_{bc,s_ros}	Scaling factor of ROS mediated beta cell apoptosis
α_{s,bcd_ros}	Scaling factor of ROS mediated beta cell damage
α_{s,ins_ffa}	Scaling factor of FFA mediated insulin production
α_{ros_inf}	Scaling factor of inflammation mediated development of ROS
α_{ros_ffa}	Scaling factor of free fatty acids mediated development of ROS

S6.5 Table. Parameters related to the pancreas module.

Name	Value	Unit	Estimation Method
$\alpha_{bc,s_{ros}}$	1 – 15	$1/[ROS\ concentration]$	Individual Calibration
$KM_{s\_ins\_glu}$	2 – 20	mM	

$\alpha_{s,bc}$	8.00×10^{-6}	Dimensionless	Collectively estimated in the baseline model
$\beta_{s,bc}$	2.00×10^{0}	Dimensionless	
$KM_{s,bc}$	2.58×10^{1}	mM	
$KM_{s,bc}$	2.58×10^{1}	mM	
$\alpha_{bc,s}$	3.77×10^{-10}	Dimensionless	
α_{s,bcd_ros}	1.20×10^{-9}	Dimensionless	
τ_{bcd}	1.00×10^{-9}	min^{-1}	
$N_{bc,SS}$	5.00×10^{2}	Dimensionless	
KM_{ros_ffa}	3.00×10^{0}	mM	
$\beta_{s.ins\_glu}$	2.00×10^{0}	Dimensionless	
