# Supplementary material for: A long-term mechanistic computational model of physiological factors driving the onset of type 2 diabetes in an individual
Source: PLoS One. 2018 Feb 14;13(2):e0192472. doi: 10.1371/journal.pone.0192472 (PMC5812629; doi:10.1371/journal.pone.0192472)
Supplement: S7 Table — (PDF) [file pone.0192472.s015.pdf]

S7 Table. Differential equations, expressions and variables of the insulin resistance compartment.

S7.1 Table. Calculations of insulin resistance component.

Variable	Equation
k_{dep}^{ISR}	$k_{dep\_ppx}^{ISR} + k_{dep\_ros}^{ISR} \times C_{ros}^{MUS} + \alpha_{dep\_ffa} \times \frac{\left(C_{ffa}^{MUS}\right)^{n_{dep\_ffa}}}{\left(k_{dep\_ffa}\right)^{n_{dep\_ffa}} + \left(C_{ffa}^{MUS}\right)^{n_{dep\_ffa}}}$
C_{ros}^{MUS}	$k_{ADP,s} \times \left(\rho_{glu,O2} \times R_{glu+ADP,ATP}^{MUS} + \rho_{ffa,O2} \times R_{ffa+ADP,ATP}^{MUS} + \rho_{pro,O2} \times R_{ketoa+ADP,ATP}^{MUS}\right)$
$InsR_{active}$	$C_{ins}^{BLD} \times \left(\frac{k_{InsR\_off}^{ISR} + k_{InsR\_active}^{ISR}}{\frac{k_{InsR\_off}^{ISR}}{k_{InsR\_on}^{ISR}} \times k_{dep}^{ISR}} - \frac{k_{InsR\_on}^{ISR}}{k_{dep}^{ISR}}\right) \times \frac{InsR_{total}}{1 + C_{ins}^{BLD} \times \left(\frac{k_{InsR\_off}^{ISR} + k_{InsR\_active}^{ISR}}{\frac{k_{InsR\_off}^{ISR}}{k_{InsR\_on}^{ISR}} \times k_{dep}^{ISR}} - \frac{k_{InsR\_on}^{ISR}}{k_{dep}^{ISR}} + \frac{k_{InsR\_on}^{ISR}}{k_{InsR\_off}^{ISR}}\right)}$
IS	$\frac{InsR_{active}}{InsR_{active\_SS}}$

S7.2 Table. Differential equations by species in insulin resistance component.

Species	Ordinary Differential Equation
Mitochondria	$\frac{dC_{mito}^{ISR}}{dt} = R_{s,mito}^{ISR} - R_{mito,deg}^{ISR}$
Cumulative Reactive Oxygen Species	$\frac{dC_{rosc}^{ISR}}{dt} = R_{ros,rosc}^{ISR}$
GSC1	$\frac{dGSC1}{dt} = R_{s,gsc1}^{ISR} + R_{glut1,gsc1}^{ISR} - R_{gsc1,deg}^{ISR} - R_{gsc1,glut1}^{ISR} - R_{gsc1,glut1\_ins}^{ISR}$
GLUT1	$\frac{dGLUT1}{dt} = R_{gsc1,glut1}^{ISR} + R_{gsc1,glut1\_ins}^{ISR} - R_{glut1,gsc1}^{ISR} - R_{gult1,deg}^{ISR}$
GSC4	$\frac{dGSC4}{dt} = R_{s,gsc4}^{ISR} + R_{glut4,gsc4}^{ISR} - R_{gsc4,glut4}^{ISR} - R_{gsc4,glut4\_ins}^{ISR} - R_{gsc4,glut4\_AMPK}^{ISR} - R_{gsc4,deg}^{ISR}$
GLUT4	$\frac{dGLUT4}{dt} = R_{gsc4,glut4}^{ISR} + R_{gsc4,glut4\_ins}^{ISR} + R_{gsc4,glut4\_AMPK}^{ISR} - R_{glut4,gsc4}^{ISR} - R_{glut4,deg}^{ISR}$

S7.3 Table. Calculation of variables in differential equations in insulin resistance component.

Variable	Equation	Ref. in Figure S6
$R_{s,gsc1}^{ISR}$	$k_{s,gsc1}$	v_1^{ISR}
$R_{gsc1,deg}^{ISR}$	$k_{gsc1,deg} * GSC1$	v_2^{ISR}
$R_{gsc1,glut1}^{ISR}$	$k_{gsc1,glut1} \times GSC1$	v_3^{ISR}
$R_{gsc1,glut1\_ins}^{ISR}$	$k_{gsc1,glut1\_ins} \times GSC1 \times InsR_{active}$	v_4^{ISR}
$R_{gult1,deg}^{ISR}$	$k_{glut1,deg} * GLUT1$	v_5^{ISR}
$R_{glut1,gsc1}^{ISR}$	$k_{glut1,gsc1} \times GLUT1$	v_6^{ISR}
$R_{s,gsc4}^{ISR}$	$k_{s,gsc4}$	v_7^{ISR}
$R_{gsc4,deg}^{ISR}$	$k_{gsc4,deg} * GSC4$	v_8^{ISR}
$R_{gsc4,glut4}^{ISR}$	$k_{gsc4,glut4} \times GSC4$	v_9^{ISR}
$R_{gsc4,glut4\_ins}^{ISR}$	$k_{gsc4,glut4\_ins} \times GSC4 \times InsR_{active}$	v_{10}^{ISR}
$R_{gsc4,glut4\_AMPK}^{ISR}$	$k_{gsc4,glut4\_AMPK} \times GSC4 \times AMPK_{active}$	v_{11}^{ISR}
$R_{glut4,deg}^{ISR}$	$k_{glut4,deg} * GLUT4$	v_{12}^{ISR}
$R_{glut4,gsc4}^{ISR}$	$k_{glut4,gsc4} \times GLUT4$	v_{13}^{ISR}
$R_{s,mito}^{ISR}$	$k_{s,mito\_AMPK} \times AMPK_{active}$	v_{14}^{ISR}
$R_{mito,deg}^{ISR}$	$k_{mito,deg} \times C_{mito}^{MUS} \times ROS_{cum}$	v_{15}^{ISR}
$R_{ROS,rosc}^{ISR}$	$k_{ros,rosc} \times C_{ros}^{MUS}$	v_{16}^{ISR}

S7.4 Table. Additional variable calculations in insulin resistance component.

Variable	Equation
$AMPK_{active}$	$\frac{kmax_{AMP,AMPK} \times (C_{AMP}^{MUS})^2}{\left(KM_{AMP,AMPK}\right)^2 + (C_{AMP}^{MUS})^2}$
$AMPK_{active0}$	$\frac{kmax_{AMP,AMPK} \times (C_{ADP}^{MUS})^2}{\left(KM_{AMP,AMPK}\right)^2 + (C_{ADP}^{MUS})^2}$
$R_{s,gsc4\_AMPK}^{ISR}$	$\begin{cases} k_{s,gsc4\_AMPK} \times (AMPK_{active} - AMPK_{active0}) \times (GSC4_{max} - GSC4)\ , & if\ AMPK_{active} > AMPK_{active0}\ and\ PA > 0 \\ 0, & otherwise \end{cases}$

S7.5 Table. Additional variable descriptions in insulin resistance component.

Variable	Description
α_{dep_ffa}	Maximal rate of free fatty acid driven dephosphorylation (inactivation) of insulin receptor
$k_{ADP,s}$	Rate of free electron leakage from the electron transport chain during oxidative phosphorylation
$InsR_{total}$	Total insulin receptors
$InsR_{active}$	Phosphorylated (active) insulin receptors
$InsR_{active\_SS}$	Baseline phosphorylated (active) insulin receptors
$k_{InsR\_on}^{ISR}$	Insulin receptor - insulin binding rate constant (k_{on})
$k_{InsR\_off}^{ISR}$	Insulin receptor – insulin dissociation rate constant (k_{off})
ROS_{cum}	ROS accumulation over time
$GLUT1$	Cell surface GLUT1 concentration
$GLUT4$	Cell surface GLUT4 concentration

Variable	Description
$GSC1$	Intracellular form of GLUT1 in GLUT1 storage compartment
$GSC4$	Intracellular form of GLUT4 in GLUT4 storage compartment
$GSC4_{max}$	Maximal concentration of GSC4

S7.6 Table. Parameters related to the insulin resistance component.

Name	Value	Unit	Estimation Method
$\alpha_{dep_{ffa}}$	1 – 30	min^{-1}	Individual calibration
k_{dep_ffa}	2 – 48	mM	
$k_{glut1,deg}$	1.20×10^{-4}	min^{-1}	Collectively estimated in the baseline model
$k_{glut4,deg}$	2.27×10^{-5}	min^{-1}	
$k_{gsc1,deg}$	5.00×10^{-4}	min^{-1}	
$k_{gsc4,deg}$	4.33×10^{-5}	min^{-1}	
$k_{glut1,gsc1}$	4×10^{-2}	min^{-1}	
$k_{glut4,gsc4}$	1×10^{0}	min^{-1}	
$k_{gsc4,glut4\_AMPK}$	7.53×10^{-1}	$min^{-1}\times mM^{-1}$	
$k_{gsc1,glut1\_ins}$	1×10^{-2}	$min^{-1}\times mM^{-1}$	
$k_{gsc4,glut4\_ins}$	4×10^{-2}	$min^{-1}\times mM^{-1}$	
$InsR_{total}$	5.30×10^{0}	pM	
$k_{dep\_ppx}^{ISR}$	2×10^{0}	min^{-1}	
$k_{InsR\_on}^{ISR}$	6×10^{-5}	$min^{-1}\times pM^{-1}$	
$k_{InsR\_off}^{ISR}$	5×10^{-2}	min^{-1}	
$k_{InsR\_active}^{ISR}$	8×10^{1}	min^{-1}	
$k_{dep\_ros}^{ISR}$	1×10^{5}	$min^{-1}\times mM^{-1}$	
n_{dep_ffa}	2×10^{0}	Dimensionless	
