# Supplementary material for: A long-term mechanistic computational model of physiological factors driving the onset of type 2 diabetes in an individual
Source: PLoS One. 2018 Feb 14;13(2):e0192472. doi: 10.1371/journal.pone.0192472 (PMC5812629; doi:10.1371/journal.pone.0192472)
Supplement: S8 Table — (PDF) [file pone.0192472.s016.pdf]

S8 Table. Model parameters estimated using data from intracellular studies.

Name	Value	Unit	Fit	References
τ_{glut1}	1.20×10^{-4}	min^{-1}	Estimated	[1–8]
τ_{glut4}	2.27×10^{-5}	min^{-1}		
τ_{gsc1}	5.00×10^{-4}	min^{-1}		
τ_{gsc4}	4.33×10^{-5}	min^{-1}		
$k_{glut1,gsc1}$	4.00×10^{-2}	min^{-1}		
$k_{glut4,gsc4}$	1.00×10^{0}	min^{-1}		
$k_{gsc4,glut4\_AMPK}$	7.53×10^{-1}	$min^{-1}\times mM^{-1}$		
$k_{gsc1,glut1\_ins}$	1.00×10^{-2}	$min^{-1}\times mM^{-1}$		
$k_{gsc4,glut4\_ins}$	4.00×10^{-2}	$min^{-1}\times mM^{-1}$		
$k_{dep\_ppx}^{ISR}$	2.00×10^{0}	min^{-1}	Estimated	[4,9–14]
$\alpha_{dep\_ros}^{ISR}$	1.50×10^{5}	Dimensionless		
γ_2	1.00×10^{0}	Dimensionless		
γ_4	2.50×10^{-1}	Dimensionless		

References:

1. Sedaghat AR, Sherman A, Quon MJ. A mathematical model of metabolic insulin signaling pathways. AmJPhysiol EndocrinolMetab. 2002;283: E1084–E1101. doi:10.1152/ajpendo.00571.2001

2. Karylowski O, Zeigerer A, Cohen A, McGraw TE. GLUT4 is retained by an intracellular cycle of vesicle formation and fusion with endosomes. Mol Biol Cell. 2004;15: 870–82. doi:10.1091/mbc.E03-07-0517

3. Huang S, Lifshitz LM, Jones C, Bellve KD, Standley C, Fonseca S, et al. Insulin stimulates membrane fusion and GLUT4 accumulation in clathrin coats on adipocyte plasma membranes. Mol Cell Biol. 2007;27: 3456–69. doi:10.1128/MCB.01719-06

4. Belfort R, Mandarino L, Kashyap S, Wirfel K, Pratipanawatr T, Berria R, et al. Dose-Response Effect of Elevated Plasma Free Fatty Acid on Insulin Signaling. Diabetes. 2005;54: 1640–1648. doi:10.2337/diabetes.54.6.1640

5. Karnieli E, Zarnowski MJ, Hissin PJ, Simpson IA, Salans LB, Cushman SW. Insulin-stimulated translocation of glucose transport systems in the isolated rat adipose cell. Time course, reversal, insulin concentration dependency, and relationship to glucose transport activity. J Biol Chem. 1981;256: 4772–7.

6. Davies SP, Helps NR, Cohen PTW, Hardie DG. 5′-AMP inhibits dephosphorylation, as well as promoting phosphorylation, of the AMP-activated protein kinase. Studies using bacterially expressed human protein phosphatase-2Cα and native bovine protein phosphatase-2Ac. FEBS Lett. 1995;377: 421–425. doi:10.1016/0014-5793(95)01368-7

7. Russell RR, Bergeron R, Shulman GI, Young LH. Translocation of myocardial GLUT-4 and increased glucose uptake through activation of AMPK by AICAR. Am J Physiol. 1999;277: H643–H649.

8. Muoio DM, Seefeld K, Witters LA, Coleman RA. AMP-activated kinase reciprocally regulates triacylglycerol synthesis and fatty acid oxidation in liver and muscle: evidence that sn-glycerol-3-phosphate acyltransferase is a novel target. Biochem J. 1999;338: 783–791. doi:10.1042/0264-6021:3380783

9. Dresner A, Laurent D, Marcucci M, Griffin ME, Dufour S, Cline GW, et al. Effects of free fatty acids on glucose transport and IRS-1-associated phosphatidylinositol 3-kinase activity. J Clin Invest. American Society for Clinical Investigation; 1999;103: 253–9. doi:10.1172/JCI5001

10. Govers R, Coster ACF, James DE. Insulin increases cell surface GLUT4 levels by dose dependently discharging GLUT4 into a cell surface recycling pathway. Mol Cell Biol. 2004;24: 6456–66. doi:10.1128/MCB.24.14.6456-6466.2004

11. Houstis NE. Reactive oxygen species play a causal role in multiple forms of insulin resistance. Massachusetts Institute of Technology. 2007.

12. Pandey KB, Mehdi MM, Maurya PK, Rizvi SI. Plasma protein oxidation and its correlation with antioxidant potential during human aging. Dis Markers. IOS Press; 2010;29: 31–36. doi:10.3233/DMA-2010-0723

13. Tsigos C, Papanicolaou DA, Kyrou I, Defensor R, Mitsiadis CS, Chrousos GP. Dose-dependent effects of recombinant human interleukin-6 on glucose regulation. J Clin Endocrinol Metab. Endocrine Society; 1997;82: 4167–70. doi:10.1210/jcem.82.12.4422

14. Sakano N, Wang D-H, Takahashi N, Wang B, Sauriasari R, Kanbara S, et al. Oxidative stress biomarkers and lifestyles in Japanese healthy people. J Clin Biochem Nutr. The Society for Free Radical Research Japan; 2009;44: 185–195. doi:10.3164/jcbn.08-252
