# Supplementary material for: A long-term mechanistic computational model of physiological factors driving the onset of type 2 diabetes in an individual
Source: PLoS One. 2018 Feb 14;13(2):e0192472. doi: 10.1371/journal.pone.0192472 (PMC5812629; doi:10.1371/journal.pone.0192472)
Supplement: S9 Table — (PDF) [file pone.0192472.s017.pdf]

S9 Table. Model parameters estimated using data from healthy metabolic studies.

Name	Value	Unit	Fit	Reference
α_{s,bcd_ros}	1.20×10^{-9}	Dimensionless	Estimated	[1–32]
$h_{ffa\_AMPK}^{BLD,MUS}$	1.87×10^{-2}	$L\times min^{-1}$		
α_{s,ins_ffa}	5.00×10^{0}	Dimensionless		
α_{lipo_PA}	6.00×10^{0}	Dimensionless		
$\alpha_{lipo\_LPA+AMPK}$	6.65×10^{-2}	Dimensionless		
$h_{aa}^{LVR,BLD}$	1.46×10^{0}	$L\times min^{-1}$		
$h_{aa}^{MUS,BLD}$	6.33×10^{0}	$L\times min^{-1}$		
$k_{ros,rosc}$	5.00×10^{-3}	$L\times min^{-1}$		
$h_{ffa}^{MUS,BLD}$	5.00×10^{-3}	$L\times min^{-1}$		
$k_{ffa+glc,tg}$	1.17×10^{-2}	min^{-1}		
$h_{glu\_GLUT1}^{BLD,X}$	3.03×10^{-1}	min^{-1}		
$k_{glu,ffa}$	3.71×10^{-1}	Dimensionless		
$k_{glu,gly}$	3.74×10^{-4}	$min^{-1} \times mM^{-1}$		
$k_{gly,glu}$	6.32×10^{-4}	min^{-1}		
$h_{keto}^{BLD,LVR}$, $h_{keto}^{LVR,BLD}$	5.00×10^{-1}	$L\times min^{-1}$		
$k_{ketoa,glu}^{LVR}$	2.88×10^{-2}	min^{-1}		
$k_{s,mito\_AMPK}$	8.00×10^{-3}	min^{-1}		
$k_{pro,aa}^{LVR}$	9.92×10^{-1}	min^{-1}		
$k_{pro,aa}^{MUS}$	1.52×10^{0}	min^{-1}		
α_{ros_ffa}	5.01×10^{1}	Dimensionless		
$k_{ros\_glu}^{BLD}$	5.78×10^{0}	min^{-1}		
$KI_{ATP,s}$	4.00×10^{1}	min^{-1}		
KI_{gngf_ins}	1.47×10^{2}	min^{-1}		
KI_{lipo_ins}	5.56×10^{-1}	min^{-1}		
$KM_{AMP,AMPK}$	1.38×10^{2}	mM		

$KM_{ffa+ADP,ATP}$	$7.22{\times}10^{-1}$	mM		
$KM_{ffa,keto}$	$1.25{\times}10^{0}$	mM		
KM_{ros_ffa}	$3.00{\times}10^{0}$	mM		
$KM_{RMR\_adaptation}$	$4.25{\times}10^{0}$	mM		
$KM_{glu+ADP,ATP}$	$8.01{\times}10^{-1}$	mM		
$KM_{s,bc}$	$2.58{\times}10^{1}$	mM		
$KM_{glu,ffa}$	$5.73{\times}10^{-1}$	mM		
KM_{s,ins_ffa}	$4.00{\times}10^{-1}$	mM		
$KM_{AMP,AMPK}$	$2.77{\times}10^{-1}$	Dimensionless		
$KM_{RMR\_adaptation}$	$1.00{\times}10^{0}$	mM		
γ_3	$1.37{\times}10^{2}$	Dimensionless		
γ_1	$1.00{\times}10^{0}$	Dimensionless		
$\beta_{RMR\_adaptation}$	$1.60{\times}10^{1}$	Dimensionless		
$\beta_{s,bc}$	$2.00{\times}10^{0}$	Dimensionless		
$\beta_{s.ins\_glu}$	$5.00{\times}10^{0}$	Dimensionless		
$\beta_{tg,ffa+glc\_ins}$	$2.00{\times}10^{0}$	Dimensionless		
$\beta_{tg,ffa+glc\_PA}$	$3.00{\times}10^{0}$<br>[Adipose]<br>$4.00{\times}10^{0}$<br>[Muscle]	Dimensionless		
β_{dep_ffa}	$2.00{\times}10^{0}$	Dimensionless		
$\beta_{s.ins\_glu}$	$2.00{\times}10^{0}$	Dimensionless		
$kmax_{AMP,AMPK}$	$3.00{\times}10^{-7}$	Dimensionless		
C_{ffa}^{ICF}	$9.00{\times}10^{-1}$	μM	Fixed	[9,10,25]

References:

1. Krogh A, Lindhard J. The relative value of fat and carbohydrate as sources of muscular energy. Biochem J. 1920;14: 290–3663.
2. Bickerton AST, Roberts R, Fielding BA, Hodson L, Blaak EE, Wagenmakers AJM, et al. Preferential uptake of dietary fatty acids in adipose tissue and muscle in the postprandial period. Diabetes. 2007;56: 168–176. doi:10.2337/db06-0822
3. Björntorp P, Bergman H, Varnauskas E. Plasma free fatty acid turnover rate in obesity. Acta Med Scand. 1969;185: 351–356. doi:10.1016/j.ecl.2008.06.007.Obesity

4. Eaton RP, Berman M, Steinberg D. Kinetic studies of plasma free fatty acid and triglyceride metabolism in man. J Clin Invest. 1969;48: 1560–79. doi:10.1172/JCI106122
5. Hudgins LC, Hellerstein MK, Seidman CE, Neese R a, Tremaroli JD, Hirsch J. Relationship between carbohydrate-induced hypertriglyceridemia and fatty acid synthesis in lean and obese subjects. J Lipid Res. 2000;41: 595–604.
6. König M, Bulik S, Holzhütter H-G. Quantifying the Contribution of the Liver to Glucose Homeostasis: A Detailed Kinetic Model of Human Hepatic Glucose Metabolism. PLoS Comput Biol. 2012;8: e1002577. doi:10.1371/journal.pcbi.1002577
7. Livesey G, Elia M. Estimation of energy expenditure , and net fat oxidation calorimetry : evaluation of errors to the detailed composition of net carbohydrate and synthesis by indirect with special reference. Clin Nutr. 1988;47: 608–628.
8. McMENAMY RH, Lund CC, Oncley JL. Unbound amino acid concentrations in human blood plasmas. J Clin Invest. 1957;36: 1672–1679. doi:10.1172/JCI103568.UNBOUND
9. Richieri G V, Kleinfeld AM. Unbound free fatty acid levels in human serum. J Lipid Res. 1995;36: 229–240.
10. Saifer A, Goldman L. The free fatty acids bound to human serum albumin. J Lipid Res. 1960;2: 268–270.
11. Calloway DH, Spector H. Nitrogen Balance as Related to Caloric and Protein Intake in Active Young Men. Am J Clin Nutr. 1954;2: 405–412.
12. Calloway DH. Nitrogen balance of men with marginal intakes of protein and energy. J Nutr. 1975;105: 914–23.
13. Young VR, Marchini JS. Mechanisms and nutritional significance of metabolic responses to altered intakes of protein and amino acids, with reference to nutritional adaptation in humans. American Journal of Clinical Nutrition. 1990. pp. 270–289.
14. Janssen I, Heymsfield SB, Wang Z, Ross R. Skeletal muscle mass and distribution in 468 men and women aged 18-88 yr. J Appl Physiol. 2000;89: 81–88.
15. Rand W, Young V, Scrimshaw N. Change of urinary nitrogen excretion in response to low-protein diets in adults. Am J Clin Nutr. 1976;29: 639–644.
16. FAO, WHO, UNU. Energy and protein requirements: Report of a Joint FAO/WHO/UNU Expert Consultation. In: Technical Report Series (WHO). No. 724. World Health Organization [Internet]. 1985 [cited 22 Apr 2015]. Available: http://www.fao.org/docrep/003/aa040e/AA040E00.htm#TOC
17. Hellerstein MK, Neese RA, Linfoot P, Christiansen M, Turner S, Letscher A. Hepatic gluconeogenic fluxes and glycogen turnover during fasting in humans. A stable isotope study. J Clin Invest. 1997;100: 1305–1319. doi:10.1172/JCI119644
18. Dulloo AG, Jacquet J. Adaptive reduction in basal metabolic rate in response to food deprivation in humans: A role for feedback signals from fat stores. Am J Clin Nutr. 1998;68: 599–606.
19. Ahlborg G, Felig P, Hagenfeldt L, Hendler R, Wahren J. Substrate Turnover during Prolonged Exercise in Man. J Clin Invest. 1974;53: 1080–1090. doi:10.1172/JCI107645
20. Guo Z, Burguera B, Jensen MD. Kinetics of intramuscular triglyceride fatty acids in exercising humans. J Appl Physiol. 2000;89: 2057–2064.
21. Jetté M, Sidney K, Blümchen G. Metabolic equivalents (METS) in exercise testing, exercise prescription, and evaluation of functional capacity. Clin Cardiol. 1990;13: 555–565. doi:10.1002/clc.4960130809
22. Van Loon LJC, Manders RJF, Koopman R, Kaastra B, Stegen JHCH, Gijsen AP, et al.

Inhibition of adipose tissue lipolysis increases intramuscular lipid use in type 2 diabetic patients. Diabetologia. 2005;48: 2097–2107. doi:10.1007/s00125-005-1889-x
23. Wadley GD, Lee-Young RS, Canny BJ, Wasuntarawat C, Chen ZP, Hargreaves M, et al. Effect of exercise intensity and hypoxia on skeletal muscle AMPK signaling and substrate metabolism in humans. Am J Physiol Metab. 2006;290: E694–E702. doi:10.1152/ajpendo.00464.2005
24. Wahren J, Felig P, Ahlborg G, Jorfeldt L. Glucose metabolism during leg exercise in man. J Clin Invest. 1971;50: 2715–2725. doi:10.1172/JCI106772
25. Young DR, Pelligra R, Adachi RR. Serum glucose and free fatty acids in man during prolonged exercise. J Appl Physiol. 1966;21: 1047–52.
26. Belfort R, Mandarino L, Kashyap S, Wirfel K, Pratipanawatr T, Berria R, et al. Dose-Response Effect of Elevated Plasma Free Fatty Acid on Insulin Signaling. Diabetes. 2005;54: 1640–1648. doi:10.2337/diabetes.54.6.1640
27. Tura A, Ludvik B, Nolan JJ, Pacini G, Thomaseth K. Insulin and C-peptide secretion and kinetics in humans: direct and model-based measurements during OGTT. Am J Physiol Endocrinol Metab. 2001;281: E966-74.
28. Hernandez TL, Sutherland JP, Wolfe P, Allian-Sauer M, Capell WH, Talley ND, et al. Lack of suppression of circulating free fatty acids and hypercholesterolemia during weight loss on a high-fat, low-carbohydrate diet. Am J Clin Nutr. 2010;91: 578–585. doi:10.3945/ajcn.2009.27909
29. Horton TJ, Drougas H, Brachey A, Reed GW, Peters JC, Hill JO. Fat and carbohydrate overfeeding effects on energy storage3 in humans : and. Am J Clin Nutr. 1995;62: 19–29.
30. Marques-Lopes I, Ansorena D, Astiasaran I, Forga L, Martínez JA. Postprandial de novo lipogenesis and metabolic changes induced by a high-carbohydrate, low-fat meal in lean and overweight men. Am J Clin Nutr. 2001;73: 253–261.
31. Schwarz JM, Linfoot P, Dare D, Aghajanian K. Hepatic de novo lipogenesis in normoinsulinemic and hyperinsulinemic subjects consuming high-fat, low-carbohydrate and low-fat, high-carbohydrate isoenergetic diets. Am J Clin Nutr. 2003;77: 43–50.
32. Krudys KM, Dodds MG, Nissen SM, Vicini P, Kevin M. Integrated model of hepatic and peripheral glucose regulation for estimation of endogenous glucose production during the hot IVGTT. Am J Physiol Metab. 2005;288: E1038–E1046. doi:10.1152/ajpendo.00058.2004.
