# Supplementary material for: A long-term mechanistic computational model of physiological factors driving the onset of type 2 diabetes in an individual
Source: PLoS One. 2018 Feb 14;13(2):e0192472. doi: 10.1371/journal.pone.0192472 (PMC5812629; doi:10.1371/journal.pone.0192472)
Supplement: S10 Table — (PDF) [file pone.0192472.s018.pdf]

S10 Table. Model parameters estimated from diabetes related studies.

Name	Value	Unit	Fit	Reference
$\rho_{pro,aa}$	5.00×10^{0}	Dimensionless	Estimated	[1–9]
X_{Fat}	9.38×10^{-2}	Dimensionless		
$\alpha_{bc,s}$	3.77×10^{-10}	Dimensionless		
$\alpha_{s,bc}$	8.00×10^{-6}	Dimensionless		
α_{gfr}	1.00×10^{0}	Dimensionless		
α_{RMR_FFM}	5.77×10^{-3}	Dimensionless		
α_{RMR_FM}	1.30×10^{-2}	Dimensionless		
α_{s,ap_tg}	2.50×10^{1}	Dimensionless		
τ_{bcd}	1.00×10^{-9}	min^{-1}		
τ_{hba1c}	3.82×10^{-6}	min^{-1}		
τ_{ins}	5.93×10^{-2}	min^{-1}		
τ_{Lp}	2.31×10^{-2}	min^{-1}		
$\alpha_{ADP,s}$	5.00×10^{-6}	Dimensionless		
$\rho_{glu,ffa}$	3.75×10^{-1}	Dimensionless		
χ_{FM}	1.50×10^{-1}	Dimensionless		
χ_{FFM}	7.54×10^{-1}	Dimensionless		
$\alpha_{aa,ketoa\_PI}^{LVR}$	1.34×10^{0}	Dimensionless		
$\alpha_{aa,ketoa\_PI}^{MUS}$	1.00×10^{1}	Dimensionless		
$k_{InsR\_active}^{ISR}$	8.00×10^{1}	min^{-1}		
$k_{InsR\_off}^{ISR}$	5.00×10^{-2}	min^{-1}		
$k_{InsR\_on}^{ISR}$	6.00×10^{-5}	min^{-1}		
$\rho_{ketoa,ATP}$	1.00×10^{0}	Dimensionless		
$\beta_{ATP,s}$	6.00×10^{0}	Dimensionless		
$\rho_{ffa,ATP}$	6.20×10^{1}	Dimensionless		
$\beta_{ffa,keto}$	6.00×10^{0}	Dimensionless		
$\rho_{ffa,O2}$	2.30×10^{1}	Dimensionless		
$\rho_{glu,ATP}$	3.30×10^{1}	Dimensionless		

$\beta_{glu,ffa}$	4.00×10^{0}	Dimensionless		
$\rho_{glu,O2}$	6.00×10^{0}	Dimensionless		
$\rho_{aa,pro}$	4.00×10^{2}	Dimensionless		
$\rho_{pro,O2}$	6.00×10^{0}	Dimensionless		
γ_{5}	2.60×10^{-1}	Dimensionless		
$InsR_{total}$	5.30×10^{0}	Dimensionless		
$N_{bc,ss}$	5.00×10^{2}	Dimensionless		
AAR_{SS}	2.00×10^{1}	Dimensionless	Fixed	[10]
C_{lp0}^{BLD}	5.00×10^{0}	mM	Fixed	[11]
V^{ECF}	1.70×10^{1}	L	Fixed	[10]

References:

1. Knowler WC, Barrett-Connor E, Fowler SE, Hamman RF, Lachin JM, Walker EA, et al. Reduction in the incidence of type 2 diabetes with lifestyle intervention or metformin. N Engl J Med. 2002;346: 393–403. doi:10.1056/NEJMoa012512
2. Pasquet P, Brigant L, Froment A, Koppert GA, Bard D, Garine I de, et al. Massive overfeeding and energy balance in men: the Guru WalIa model. Am J Clin Nutr. 1992;56: 483–490.
3. Clark A, Jones LC, de Koning E, Hansen BC, Matthews DR. Decreased insulin secretion in type 2 diabetes: a problem of cellular mass or function? Diabetes. 2001;50 Suppl 1: S169-71.
4. Meier JJ, Bonadonna RC. Role of Reduced β-Cell Mass Versus Impaired β-Cell Function in the Pathogenesis of Type 2 Diabetes. Diabetes Care. 2013;36.
5. Dulloo AG, Jacquet J. Adaptive reduction in basal metabolic rate in response to food deprivation in humans: A role for feedback signals from fat stores. Am J Clin Nutr. 1998;68: 599–606.
6. Nelson KM, Weinsier RL, Long CL, Schutz Y. Prediction of resting energy expenditure from fat-free mass and fat mass. Am J Clin Nutr. 1992;56: 848–856.
7. Nielsen S, Hensrud DD, Romanski S, Levine J a, Burguera B, Jensen MD. Body composition and resting energy expenditure in humans: role of fat, fat-free mass and extracellular fluid. Int J Obes Relat Metab Disord. 2000;24: 1153–1157. doi:10.1038/sj.ijo.0801317
8. Sedaghat AR, Sherman A, Quon MJ. A mathematical model of metabolic insulin signaling pathways. AmJPhysiol EndocrinolMetab. 2002;283: E1084–E1101. doi:10.1152/ajpendo.00571.2001
9. De Gaetano A, Hardy T, Beck B, Abu-Raddad E, Palumbo P, Bue-Valleskey J, et al. Mathematical models of diabetes progression. Am J Physiol Endocrinol Metab. 2008;295: E1462-79. doi:10.1152/ajpendo.90444.2008
10. Hall JE, Guyton AC. Guyton and Hall Textbook of Medical Physiology. Journal of Chemical Information and Modeling. 2011. doi:10.1017/CBO9781107415324.004
11. Ruhl CE, Harris TB, Ding J, Goodpaster BH, Kanaya AM, Kritchevsky SB, et al. Body mass index and serum leptin concentration independently estimate percentage body fat in older adults. Am J Clin Nutr. 2007;85: 1121–6.
