# Supplementary material for: A long-term mechanistic computational model of physiological factors driving the onset of type 2 diabetes in an individual
Source: PLoS One. 2018 Feb 14;13(2):e0192472. doi: 10.1371/journal.pone.0192472 (PMC5812629; doi:10.1371/journal.pone.0192472)
Supplement: S11 Table — (PDF) [file pone.0192472.s019.pdf]

S11 Table. Initial conditions at age 20.

Name	Value	Unit
BMI	2.30×10^{1}	$kg\times m^{-2}$
C_{ATP}^{MUS}	2.86×10^{2}	mM
C_{ins}^{BLD}	5.00×10^{1}	pM
C_{aa}^{BLD}	3.00×10^{0}	mM
C_{aa}^{LVR}	3.78×10^{-1}	mM
C_{aa}^{MUS}	4.19×10^{-1}	mM
C_{chy}^{BLD}	9.96×10^{-1}	mM
C_{ffa}^{ADI}	1.44×10^{2}	mM
C_{ffa}^{BLD}	4.00×10^{-1}	mM
C_{ffa}^{LVR}	5.84×10^{0}	mM
C_{ffa}^{MUS}	9.00×10^{-1}	mM
C_{glc}^{ADI}	2.00×10^{-1}	μM
C_{glc}^{BLD}	6.00×10^{-2}	mM
C_{glc}^{LVR}	6.00×10^{-3}	mM
C_{glc}^{MUS}	8.00×10^{-2}	mM
C_{glu}^{BLD}	4.00×10^{0}	mM
C_{glu}^{LVR}	4.00×10^{-1}	mM
C_{glu}^{MUS}	4.00×10^{-1}	mM
C_{gly}^{LVR}	7.93×10^{1}	mM
C_{gly}^{MUS}	2.03×10^{1}	mM
C_{hba1c}^{BLD}	4.75×10^{0}	%
C_{keto}^{BLD}	1.40×10^{-2}	mM
C_{keto}^{LVR}	1.80×10^{-2}	mM
C_{ketoa}^{LVR}	7.55×10^{-2}	mM
C_{ketoa}^{MUS}	8.39×10^{-2}	mM
C_{pro}^{LVR}	4.72×10^{0}	mM
C_{pro}^{MUS}	5.24×10^{0}	mM
C_{tg}^{ADI}	9.02×10^{3}	mM
C_{tg}^{BLD}	1.50×10^{0}	mM
C_{tg}^{LVR}	2.84×10^{1}	mM
C_{tg}^{MUS}	2.25×10^{1}	mM
M_{FM0}	1.12×10^{1}	kg
$GLUT1$	6.50×10^{-1}	Dimensionless
$GLUT4$	1.30×10^{-1}	Dimensionless
$GSC1$	3.50×10^{-1}	Dimensionless
$GSC4$	8.70×10^{-1}	Dimensionless

Glycogen	5.00×10^{-1}	kg
N_{bc}	5.00×10^{2}	Dimensionless
Protein	7.20×10^{0}	kg
bcd	0.00×10^{0}	Dimensionless
C_{mito}^{ISR}	1.00×10^{0}	Dimensionless
ROS_cum	1.00×10^{0}	$mM * min$
BW	6.95×10^{1}	kg
