# Supplementary material for: A long-term mechanistic computational model of physiological factors driving the onset of type 2 diabetes in an individual
Source: PLoS One. 2018 Feb 14;13(2):e0192472. doi: 10.1371/journal.pone.0192472 (PMC5812629; doi:10.1371/journal.pone.0192472)
Supplement: S12 Table — (PDF) [file pone.0192472.s020.pdf]

S12 Table. The placebo population median values of the standard deviation and coefficient of variation of estimated parameters.

**Parameter Number**	**Parameter abbreviation**	**Standard Deviation, $\bar{\sigma}_p$**	**Coefficient of Variation, $\overline{cv}_p$ (%)**
1	CI_0	1.55	1.15
2	FI_0	1.51	3.08
3	CI/CI_0	0.154	10.3
4	FI/FI_0	0.146	11.1
5	CI_2/CI_0	0.223	15.8
6	FI_2/FI_0	0.219	16.7
7	$Cmax_{hba1c}^{BLD}$	3.37	8.15
8	$C_{hba1c}^{BLD}(t=0)$	0.221	4.84
9	α_{dep_ffa}	1.78	11.8
10	k_{dep_ffa}	1.76	9.54
11	α_{bc,s_ros}	1.53	17.5
12	KM_{s,ins_glu}	1.61	12.3
