# Supplementary material for: A long-term mechanistic computational model of physiological factors driving the onset of type 2 diabetes in an individual
Source: PLoS One. 2018 Feb 14;13(2):e0192472. doi: 10.1371/journal.pone.0192472 (PMC5812629; doi:10.1371/journal.pone.0192472)
Supplement: S13 Table — (PDF) [file pone.0192472.s021.pdf]

**S13 Table. The placebo population mean correlation matrix of the 12 parameters fit to individual subjects**

| | CI_b0 | FI_b0 | CI | FI | CI_2 | FI_2 | C_hba1c_max | C_hba1c_b | max_ri_inact_ffa | k_ri_inact_ffa | alpha_apoptosis_ros | km_insulin_glu |
|---|---|---|---|---|---|---|---|---|---|---|---|---|
| CI_b0 | 1 | -0.0163883 | 0.03793091 | -0.01006 | 0.03454182 | -0.0522636 | 3.83E-06 | -0.0047829 | 0.020761603 | 0.005838721 | -0.018841342 | -0.011532632 |
| FI_b0 | -0.0163883 | 1 | 0.03891499 | 0.01365506 | -0.0286541 | 0.07394052 | 4.59E-06 | 0.00013458 | 0.062805597 | 0.024594444 | 0.038842078 | 0.00983369 |
| CI | 0.03793091 | 0.03891499 | 1 | -0.0333387 | 0.00088321 | 0.01893273 | 4.61E-06 | -0.0497576 | 0.005157198 | -0.010228062 | -0.017063227 | -0.019104123 |
| FI | -0.01006 | 0.01365506 | -0.0333387 | 1 | -0.0433538 | 0.01380327 | -1.51E-06 | 0.00472555 | 0.001401766 | 0.048758551 | -0.05574075 | 0.020133369 |
| CI_2 | 0.03454182 | -0.0286541 | 0.00088321 | -0.0433538 | 1 | -0.0181187 | 8.30E-06 | -0.007527 | -0.007957882 | -0.036705742 | 0.02126518 | -0.014915813 |
| FI_2 | -0.0522636 | 0.07394052 | 0.01893273 | 0.01380327 | -0.0181187 | 1 | -3.26E-05 | 0.00807381 | -0.00314314 | 0.04365417 | 0.021427552 | 0.046663302 |
| C_hba1c_max | 3.83E-06 | 4.59E-06 | 4.61E-06 | -1.51E-06 | 8.30E-06 | -3.26E-05 | 1 | -0.1493804 | -3.98E-06 | 4.38E-05 | -4.55E-06 | 7.28E-06 |
| C_hba1c_b | -0.0047829 | 0.00013458 | -0.0497576 | 0.00472555 | -0.007527 | 0.00807381 | -0.149380393 | 1 | 0.002990566 | 0.001108864 | -0.001188215 | -0.004680016 |
| max_ri_inact_ffa | 0.0207616 | 0.0628056 | 0.0051572 | 0.00140177 | -0.0079579 | -0.0031431 | -3.98E-06 | 0.00299057 | 1 | 0.025468433 | 0.069545458 | -0.001175402 |
| k_ri_inact_ffa | 0.00583872 | 0.02459444 | -0.0102281 | 0.04875855 | -0.0367057 | 0.04365417 | 4.38E-05 | 0.00110886 | 0.025468433 | 1 | -0.075264916 | 0.033751093 |
| alpha_apoptosis_ros | -0.0188413 | 0.03884208 | -0.0170632 | -0.0557408 | 0.02126518 | 0.02142755 | -4.55E-06 | -0.0011882 | 0.069545458 | -0.075264916 | 1 | -0.026466024 |
| km_insulin_glu | -0.0115326 | 0.00983369 | -0.0191041 | 0.02013337 | -0.0149158 | 0.0466633 | 7.28E-06 | -0.00468 | -0.001175402 | 0.033751093 | -0.026466024 | 1 |
