# Supplementary material for: A long-term mechanistic computational model of physiological factors driving the onset of type 2 diabetes in an individual
Source: PLoS One. 2018 Feb 14;13(2):e0192472. doi: 10.1371/journal.pone.0192472 (PMC5812629; doi:10.1371/journal.pone.0192472)
Supplement: S1 Text — (DOCX) [file pone.0192472.s022.docx]

**S1 Text. Model Sensitivity Analysis.**

A sensitivity analysis of the model outputs to the 12 calibrated parameters (Table 3) was performed to determine the dependence of the model behavior on these parameters. For a given parameter vector $\boldsymbol{\theta}$, for an individual, the squared error, Φ, summed over all 4 model outputs (weight, *W*, blood concentration of HbA1c, $C_{hba1c}^{BLD}$, blood concentration of glucose,$C_{glu}^{BLD}$, and blood concentration of insulin, $C_{ins}^{BLD}$ ) was

$$\Phi_{j}\left( \boldsymbol{\theta} \right)=\sum_{i} {w_{j}\left( y_{ij}\left( \boldsymbol{\theta} \right) - y_{ij}^{*} \right)}^{2}$$

where $y_{i,j}^{*}$ is the measured value of variable *j* at time *i* within the first 3 years of the study, $y_{i,j}$ is the corresponding model simulation value of variable *j*, and $j\in\{W, C_{hba1c}^{BLD}, C_{glu}^{BLD}, C_{ins}^{BLD}\}$, and *w_j_* was the weight assigned to each output $(W=1.0, C_{hba1c}^{BLD}=10.0, C_{glu}^{BLD}=1.0, C_{ins}^{BLD}=1.0)$. We also define $\Phi_{j}^{0}=\Phi_{j}\left( \boldsymbol{\theta}_{\boldsymbol{0}} \right)$, where $\boldsymbol{\theta}_{\boldsymbol{0}}$ is the vector of optimized values of the 12 parameters for each subject. Further, define $\Phi_{j,k}^{'}=\Phi_{j}\left( \boldsymbol{\theta}_{\boldsymbol{k}} \right)$ where $\boldsymbol{\theta}_{\boldsymbol{k}}$ is obtained by adding a small perturbation $\epsilon$ to the *k^th^* element of $\boldsymbol{\theta}_{\mathbf{0}}$, i.e., if $\boldsymbol{\theta}_{0}=\left( \theta_{1},\ldots,\theta_{k}, \ldots, \theta_{12} \right)$, then $\boldsymbol{\theta}_{k}=\left( \theta_{1},\ldots,\theta_{k}+\epsilon, \ldots, \theta_{12} \right)$

The sensitivity of variable *j* to the *k^th^* parameter is defined as

$$S_{jk}=\frac{{|\Phi}_{j,k}^{'}-{\Phi_{j}^{0}|}}{\Phi_{j}^{0}}$$

and was evaluated for all subjects in the placebo arm of the study. This allowed for comparison of sensitivities across different outputs and parameters over the entire population of placebo subjects. The results of the sensitivity are plotted in Figure S7.

To measure the level of confidence in the estimates of the model parameters, we first calculated the Fisher information matrix (FIM). The FIM allowed us to estimate the covariance matrix, *cov*, to derive the standard deviation of the parameter estimates, the correlation matrix to examine if there were significant correlations between parameter estimates, and the coefficients of variation to to determine the relative variability of the parameter estimates. The FIM is typically defined in terms of the negative log-likelihood for subject *y* as,

$$I(\boldsymbol{\theta})=-E\left[ \frac{\partial^{2}\ln f(y|\boldsymbol{\theta})}{\partial\theta^{2}} \right]$$

Here the minimized objective function, Φ, was used, the error in the biomarker measurements were assumed to be uncorrelated and normally distributed with zero mean and constant variance, and the model was assumed to be accurate. This resulted in the following expression for the individual observed FIM, *J*, in terms of the 12x12 Hessian matrix, **H,**

$$J(\boldsymbol{\theta})=\frac{1}{\Sigma} \frac{\partial^{2}\Phi(\boldsymbol{\theta})}{\partial\theta_{j}\partial\theta_{k}} = \frac{1}{\Sigma} \mathbf{H}\boldsymbol{(}\Phi(\boldsymbol{\theta})), j=1, \ldots, 12, k=1, \ldots, 12,$$

$${\Sigma=\sigma_{e}^{2}\boldsymbol{I}, and \sigma}_{e}^{2}=\frac{{\Phi^{0}}}{n-p} .$$

Here, Σ is the covariance of the measurements, with constant variance $\sigma_{e}^{2}$, *Φ^0^* was the objective score when all the model parameters, *θk* (*k* = 1, ..., 12), were at their optimal estimates, *n* is the total number of biomarker data points, and *p* is the number of estimated model parameters. The Hessian was numerical estimated by running the model for perturbed values of *j* and *k* and numerically computing the second derivative using a centered difference scheme.

The covariance matrix of the estimated parameters, *cov*(***θ***), was estimated from the observed FIM as,

$$cov\left( \boldsymbol{\theta} \right)=\left( \boldsymbol{H}+\lambda\boldsymbol{I} \right)^{-1}$$

where the Hessian was regularized with the Tikhonov regularization parameter, λ, set to 0.001 and **I** was the identity matrix. The regularization term was included to avoid singularity problems during matrix inversion. For each parameter, for each placebo subject, we estimated the standard deviation of the optimal fit, $\sigma\left( \theta_{k} \right)$, the coefficient of variance, $cv\left( \theta_{k} \right)$, and calculated the medians of these two statistics over the placebo population (n = 331 subjects),

$$\sigma_{p}\left( \theta_{k} \right)= \sqrt{{cov(\theta\boldsymbol{)}}_{kk}}, k=1,2,\ldots,12 ,$$

$$\bar{\sigma}_{p}\left( \theta_{k} \right)= median of \sigma\left( \theta_{k} \right) over population of placebo subjects,$$

$${cv}_{p}\left( \theta_{k} \right)=\frac{\sqrt{{cov(\theta)}_{kk}}}{\theta_{k0}} , k=1,2,\ldots,12,$$

$$\bar{cv}_{p}\left( \theta_{k} \right)=median of cv\left( \theta_{k} \right) over population of placebo subjects,$$

where *θ_k0_* was *θ_k_* at its optimal value. The values of $\bar{\sigma}_{p}\left( \theta_{k} \right)$ and $\bar{cv}_{p}\left( \theta_{k} \right)$ are listed in Table S12. Among the 12 parameters estimated, the correlation matrix of the model parameters *corr*(*θ*) was obtained from the covariance matrix as,

$${corr\left( \theta\right)}_{mn}=\frac{cov(\theta_{m}, \theta_{n})}{\sqrt{var\left( \theta_{m} \right)} \sqrt{var(\theta_{n})}}=\frac{{cov\left( \theta\right)}_{mn}}{\sqrt{{cov\left( \theta\right)}_{mm}}\sqrt{{cov\left( \theta\right)}_{nn}}}$$

where $k, p=1,2,\ldots,12$, and the placebo population mean correlation matrix, $\bar{corr}\left( \theta\right),$ was generated by taking average over all the individual correlation matrices, *corr*(*θ*)

$$\bar{corr}\left( \theta\right)=average of corr\left( \theta\right) over population of placebo subjects.$$

The placebo population mean correlation matrix is listed Table S13. In general the parameters were not correlated, the correlation between parameters in $\bar{corr}\left( \theta\right)$ were all <= 0.1. The largest correlation was between C_hba1c_max and C_hba1c_b which had a value of -0.149. To explore the relationship of the dependency between these two parameters a fitness landscape plot was generated and is shown in Figure S8. The plot was calculated by perturbing all combinations of C_hba1c_max and C_hba1c_b in increments of 2% to +/-10% of the optimal values of the parameters, resulting in an 11x11 matrix of objective function values, Φ. From examination of Figure S8, the model is more sensitive to perturbation in C_hba1c_b, with a maximum change in Φ of ~25% and weaker sensitivity to C_hba1c_max, with changes in Φ of < 10%.
